# First Evaluation of an Index of Low Vagally-Mediated Heart Rate Variability as a Marker of Health Risks in Human Adults: Proof of Concept

**DOI:** 10.3390/jcm8111940

**Published:** 2019-11-11

**Authors:** Marc N. Jarczok, Julian Koenig, Arne Wittling, Joachim E. Fischer, Julian F. Thayer

**Affiliations:** 1 Department of Psychosomatic Medicine and Psychotherapy, Ulm University Medical Center, 89081 Ulm, Germany; 2 Mannheim Institute of Public Health, Social and Preventive Medicine, Mannheim Medical Faculty, Heidelberg University, 68167 Mannheim, Germany; Joachim.Fischer@medma.uni-heidelberg.de; 3 Section for Translational Psychobiology in Child and Adolescent Psychiatry, Department of Child and Adolescent Psychiatry, Centre for Psychosocial Medicine, University of Heidelberg, 69115 Heidelberg, Germany; Julian.Koenig@med.uni-heidelberg.de; 4 University Hospital of Child and Adolescent Psychiatry and Psychotherapy, University of Bern, CH-3000 Bern, Switzerland; 5 Center for Neuroscience Research NPO, 54296 Trier, Germany; arne-wittling@znf-gmbh.com; 6 Department of Psychological Science, The University of California, Irvine, Irvine, CA 92697-7085, USA; jfthayer@uci.edu

**Keywords:** heart rate variability, novel risk factor, CVD, prevention, risk stratification

## Abstract

Multiple studies have demonstrated low vagally-mediated heart rate variability (HRV) being associated with a range of risk factors for heart disease and stroke, including inflammation, hyperglycemia, hyperlipidemia, and hypertension. Yet, no cut point exists that indicates elevated risk. In the present study we sought to identify a cut point-value for HRV that is associated with elevated risk across a range of known risk factors. Methods: A total of 9550 working adults from 19 study sites took part in a health assessment that included measures of inflammation, hyperglycemia, hyperlipidemia, and hypertension and vagally-mediated HRV (Root mean square of successive differences between normal heartbeats (RMSSD)). Multiple age and sex adjusted logistic regressions were calculated per risk factor (normal versus clinical range), with RMSSD being entered in binary at different cut points ranging from 15–39 msec with a 2 msec increment. Results: For daytime RMSSD, values below 25 ± 4 indicated elevated risk (odds ratios (OR) 1.5–3.5 across risk factors). For nighttime RMSSD, values below 29 ± 4 indicated elevated risk (OR 1.2–2.0). Conclusion: These results provide the first evidence that a single value of RMSSD may be associated with elevated risk across a range of established cardiovascular risk factors and may present an easy to assess novel marker of cardiovascular risk.

## 1. Introduction

Research from the past decades has demonstrated that low vagally-mediated heart rate variability (HRV) is predictive of cardiac and all-cause mortality [1,2,3]. Furthermore, HRV is associated with a wide range of risk factors for heart disease and stroke, including elevated low-grade inflammation, hyperglycemia, hyperlipidemia, and hypertension [4,5,6,7,8,9,10]. Similarly, decreased HRV correlates with increased cardiovascular risk scores [11,12]. In the majority of other clinically health-related risk factor measures, such as blood pressure, heart rate, or glucose status, established clinical cut points are routinely applied to provide an indication of current health status and future risk. However, to date, clinicians in search of existing diagnostic reference levels (DRLs) in HRV that indicate elevated risk have had little guidance.

Some HRV cutoff values have been proposed but their generalizability is often limited because they were derived in specific subgroups, i.e., patients or children [13,14], where the chosen HRV parameter cutoff is unexplained, or based on short term recordings [14,15,16] (<15 min), ignoring apparent circadian rhythms [17,18] in HRV parameters.

Heart rate variability cutoff values may be useful in health care settings to determine at risk populations (i.e., risk stratification) prior to the development of certain clinical conditions. Decreased HRV values are known to be associated with a wide range of clinical conditions, such as hypertension, type 2 diabetes, obesity, or depression [1,4,19]. Therefore, HRV is often criticized as being non-specific. Yet, because of its non-specificity to any particular disease, it may serve as an easy to assess and cheap antecedent risk marker of exposure at an early etiology stage, for example, in the primary care setting of company physicians.

Clearly, there is a need to assess the value of novel markers of cardiovascular disease risk. Accurate assessment of cardiovascular risk is essential not only for clinical decision making, but also for primary prevention, because the benefits, risks, and costs of alternative management strategies must be weighed to choose the best prevention strategy or treatment for the individual.

Several statistical methods for the assessment of the usefulness of new biomarkers have been reviewed [20,21,22,23], and recommendations for the reporting of novel risk markers have been proposed [24]. Specifically, guidelines from the American Heart Association [22] suggest that the evaluation of novel biomarkers proceed in phases, with the first phase being proof of concept, in which it is shown that the novel biomarker differs between those with and without the relevant outcome. In addition, these guidelines give recommendations for the reporting of studies that evaluate novel biomarkers. The reporting recommendations include conducting the study in accordance with standards for observational studies, reporting levels of the standard risk factors, and reporting the odds ratio (OR) or related statistics, including confidence intervals (CI), associated with proposed risk factor. In the present study we sought to use these guidelines to identify a value for HRV for use in primary prevention that is associated with elevated cardiovascular disease risk factors across a range of established but noninvasive risk factors. Specifically, we report the results of an observational study in employees with standard risk factors using odds ratios associated with the discrimination between individuals in the normal range versus those in the clinical range, with a time domain measurement of HRV as an independent variable.

## 2. Material and Methods

### 2.1. Study Population

We utilized a large cross-sectional, secondary data set from the Mannheim Industrial Cohort Study (MICS). The data were collected as part of a voluntary health risk assessment that was offered to all employees during working hours. An agent independent from the employer conducted the health risk assessments and data collection (HealthVision Ltd., Berlingen, Switzerland). A total of 19,985 participants from 30 study sites (companies from the secondary and tertiary sectors; response rate 55% (ranging from 49%–60%)) across Germany were eligible for this study. Data were collected between 2010 and 2014.

In brief, participants were invited to take part in a “Work Health Check” and were offered a detailed individual report containing their health status as assessed by medical examination and self-reports. This sample encompassed the entire workforce between 18 and 65 years.

The Ethical Committee of the Mannheim Medical Faculty, Heidelberg University approved the secondary analysis of this data (2010-296E-MA). All participants gave written informed consent prior to examination.

### 2.2. Exclusion Criteria

Participants were excluded from all analysis based on their self-reported diagnosis by a physician: respiratory diseases (e.g., Asthma, chronic obstructive pulmonary disease (COPD)), angina pectoris, stroke, infarction, Coronary Heart Disease (CHD), depression, burnout, other chronic diseases, and cancer (12.84% of 19,985). Also, participants indicating a daily analgesic, hypnotic, or calmative intake were excluded (0.83%).

In addition, participants with incomplete psychobiological data (see Section 2.3: Measures) had to be excluded due to missing HRV data (42.56%) or other data (2.99%).

Participants in the preclinical range or in the second tertile (see Table 1) were excluded from this analysis to contrast normal versus clinical values of risk markers. The normal versus preclinical contrasting is beyond the scope of this paper.

### 2.3. Measures

Demographic, medical, and lifestyle variables were obtained from an online questionnaire. The questionnaire had to be completed prior to being able to schedule a medical examination. All participants were enrolled and examined between 10:00 and 17:00 on a typical workday (Monday to Friday) during work hours. Upon arrival, a medical examination was performed and the heart rate (HR)-recorder was attached. The next morning, between 7:00 and 9:00, a fasting blood sample was collected from all individuals. Samples were transported to a commercial laboratory (Synlab, Augsburg, Germany) within two hours of sample collection and analyzed within 24 h.

All measures were categorized according to standard clinical cutoffs (http://www.synlab.com/en/human/parameter-index/international-parameter-index/) (as far as existing) or categorized into tertiles (described in Table 1). During the course of this study the American College of Cardiology/American Heart Association guidelines for hypertension changed (ACC/AHA 2017). We used the most recent cut points in the current analyses, such that those classified in the clinical range corresponded to those with stage 2 hypertension in the new guidelines. Participants indicating the usage of medication such as anti-hypertensive, lipid lowering, or glucose lowering drugs or reporting corresponding physician diagnoses were re-assigned as cases on the respective biomarker.

#### 2.3.1. Blood withdrawal

Inflammatory markers (high sensitivity C-reactive protein (CRP) (CardioPhase hsCRP, DADE BEHRING)), white blood count (WBC)), blood glucose (fasting plasma glucose (FPG) OSR6121, Olympus; glycosylated hemoglobin (HbA_1c_) “Tina-quant, Hemoglobin A1c Gen.2”, Roche Diagnostics [25]), and blood lipids (Total cholesterol (TC), triglyceride (TRI) OSR60118, Olympus), low density lipoprotein (LDL), high density lipoprotein (HDL) OSR6187, Olympus) were determined using routine laboratory analyzers (for details see [26]).

#### 2.3.2. Blood Pressure

Using the oscillometric technique, blood pressure (BP) was recorded twice using the CRITIKON Dinamap Portable Adult/Pediatric and Neonatal Vital Signs Monitor (Model 8100). Measurements were taken from the dominant arm in the seated position after a five-minute rest period. A study physician repeated the reading using sphygmomanometry if one or more BP values exceeded 135 mmHg (systolic) or 90 mmHg (diastolic). The arithmetic mean of all two to three measurements was calculated.

#### 2.3.3. Heart Rate Monitoring

Heart rate (HR) was recorded as beat-to-beat intervals (IBI) using a t6 Suunto Memory Belt^®^ (SuuntoVantaa, Finland), sampling at a rate of 1000 Hz. The Suunto Memory Belt^®^ is a reliable measure of electrocardiography (ECG) compared to a five lead ECG [27].

IBIs were determined as the interval between two successive R-spikes. After attaching the ambulatory HR recorder, participants commenced their routine work duties, followed by after work leisure and sleep activities. Participants were asked to record their sleep routine protocol and to return the HR recorder after a minimum of 22 h of wearing or in case of any difficulties.

The root mean square of successive differences between normal heartbeats (RMSSD) was calculated from 24-h long-term HR monitoring (beat to beat) derived from 5.35-min averages recorded for day time and night time as indicators of vagal tone. This index uses what the econometrics literature calls first-differencing and acts like a high pass filter, thus removing long-term trends and slower-frequency variability from the signal. Because of the frequency characteristics of the autonomic influences on the heart, such that vagal influences cover the full frequency range and sympathetic influences are primarily restricted to the lower frequencies, RMSSD reflects primarily vagal influences [28,29]. In addition, whereas spectral-derived indices of HRV are common in the literature, the exact values obtained vary as a function of choice of algorithm, detrending methods, and other computational parameters [23,30]. RMSSD provides an index in a common metric (milliseconds) and is thus better suited for use as a biomarker in a clinical setting.

Researchers at the Center for Neuropsychological Research (University of Trier, 54296 Trier, Germany) analyzed the raw IBIs according to the Task Force Guidelines of the European Society of Cardiology and the North American Society of Pacing and Electrophysiology [30]. The ANS-Explorer Software [31] was used to calculate the RMSSD in all valid adjacent IBIs if the artifact rate of the respective 335 second (s) segment was below 5%. The artifact correction is part of the NEUROCOR^®^ precisionHRV-Algorithm (NEUROCOR ltd. Trier, Germany). The algorithm marked all RR-intervals as artifacts if they deviated from a moving average of five RR-intervals by an age-dependent amount. Non-physiological RR-intervals, e.g., due to a too fast, too slow, or too quickly changing heart rate, were also marked as artifacts. In order to detect artefacts caused by arrhythmias in persons with pronounced variability, the RR-intervals were finally assessed with a statistical outlier test. The correction of the RR-intervals marked as artifacts was carried out without any violation of the phase relationship of the signal and a change in the total time. The affected areas were not cut out, but replaced by spline interpolated RR-intervals of the same length. For larger artifact phases, the distribution of the R-times was adapted to the R-time dynamics before and after the defect. The number of replaced RR-intervals was counted (i.e., artifact rate). This led to an exclusion of 5.40% segments from total of 2,551,965 segments (prior to any other exclusion of participants, e.g., due to missing questionnaire or medical information). To avoid the case of retaining an individual HRV dataset with only a few valid 335 s intervals, the number of valid 335 s segments had to be ≥30 for night time and ≥50 for daytime. This corresponded to a minimum valid recording time of ~2.5hours night time and ~4.5hours for daytime, leading to an exclusion of 7.4% of recordings (again, prior to any other exclusion of participants, e.g., due to missing questionnaire or medical information).

### 2.4. Statistical Methods

Logistic regression models were calculated to quantify odds ratios for various risk factors as dependent variables, comparing individuals in the normal versus clinical range by HRV (independent variable) during HRV nighttime and daytime recording. For example, the odds ratio was calculated as having blood pressure in the clinically elevated (≥140/90 mmHg) versus normal range (<120/80 mmHg) with an RMSSD during night above versus below 30 msec).

We calculated 24 models per risk factor: 12 for the day time period and 12 for the night time period, in three steps. The model included age, sex, and heart rate by using the heart period (i.e., IBI) as recommended by [32], as independent variables. In addition, the model included the binary RMSSD cutoff values from daytime (or nighttime) and increased the cutoff of by two milliseconds, starting with RMSSD < 15 ms, then RMSSD < 17 ms and so on until RMSSD < 39 was reached. As we were interested in the performance of HRV as an integrative marker for psychosomatic health, we abstained from adjusting the regression models for further known influencing factors on HRV, such as work stress or physical fitness.

All linear regression models were corrected for the nested structure of employees in companies using the Stata *vce(cluster)* option [33]. Here, standard errors were adjusted for intragroup correlation on the company level, relaxing the usual requirement of the observations (i.e., employees) to be independent of each other.

We compared all odds ratios (OR) and their respective 95% confidence intervals (CI) between all models using forest plots. We used the forest plot for blood pressure as a benchmark to get a first approximation of an HRV value to be examined across the various established biomarkers. We then used that value to examine the value of HRV associated with values in the clinical range of the other biomarkers. All analyses were conducted using Stata (v15.1 SE, College Station, TX, USA: StataCorp LP).

## 3. Results

A total of 9550 participants from 19 study sites (48%) out of the eligible 19,985 participants (30 study sites) had complete data on all variables of interest (*n* = 8425 for night time HRV; 42%). The mean age was 41.7 (Standard deviation (SD) 11.0) and 18.9% were female. The overall RMSSD mean was 27.8 msec (SD 12.2) during day time and 38.9 msec (SD 19.3) during nighttime. Further sample characteristics and prevalence of clinical cutoffs are depicted in Table 1 and Table 2. The primary analysis compared the normal versus clinical population. Therefore, all participants in the preclinical range of the risk marker were excluded from analysis, resulting in varying analysis samples (see Table 2). For example, the fasting glucose models included a total of 93.42% from 9550 participants, since 6.48% were in the preclinical range of FPG (100–126 mm/dL; see Table 1 and Table 2). In turn, the HbA_1c_ models included a total of 75.90% from 9550 participants, since 24.10% were in the preclinical range of HbA_1c_ (6–6.5%; see Table 1 and Table 2).

Scatterplots containing the predicted values from linear regression models adjusted for age and sex indicated a relationship between the several biomarkers and RMSSD values. A fractional polynomial function represented the average predicted value stratified by sex and included the 95% confidence interval (CI; shaded area; see supplementary material figures Figure S1–Figure S20). In summary, the associations between RMSSD and the risk markers (continuous) showed in a curvilinear fashion with inflection points, with values towards the clinically relevant areas being associated with lower levels of RMSSD in both men and women. In addition, the unadjusted plain associations are shown in scatterplots in the supplementary material figures (Figure S21–Figure S40), stratified by sex and again with a fractional polynomial function.

### Multiple Adjusted Logistic Regression Models

**Figure 1:**
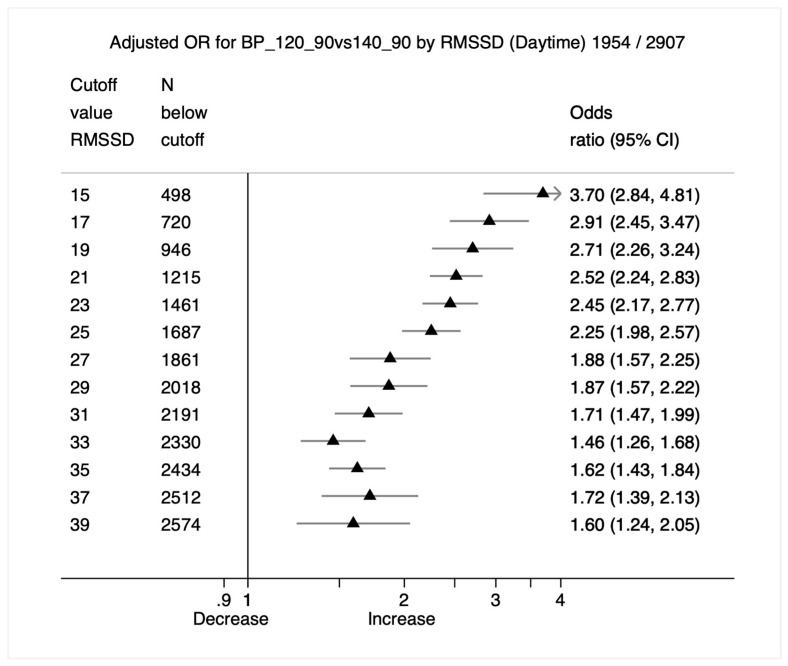
Forest plot: prediction of root mean square of successive differences between normal heartbeats (RMSSD) for hypertension.

Graphical analysis using 18 forest plots showing adjusted odds ratio and corresponding 95% confidence intervals (CI) per risk factor by the cutoff value range of RMSSD were designed to aid decision making. Here, a clear dose response relationship for both white blood count and low-grade inflammation, as indicated by CRP, could be observed. An example for blood pressure is included (see Figure 1), the other figures are enclosed in the online supplementary files (Figure S41–S57).

For daytime RMSSD a value of 25 ± 4 msec and below indicated elevated risk, as indexed by clinical cut points for CRP, WBC, FPG, HbA_1c_, LDL, HDL, TRI, and BP (OR range from 1.35 to 3.45). For nighttime RMSSD a value of 29 ± 4 and below indicated elevated risk, as indexed by the clinical cut points for CRP, WBC, FPG, HbA_1c_, LDL, and BP (OR range 1.16–2.00), but not for HDL and TRI. Overall, daytime values appeared to be stronger predictors of clinical outcomes than nighttime values.

The forest plot shows the odds ratios with according 95%CI estimates per RMSSD cutoff (msec).

An odds ratio larger than 1 indicates an increased risk for hypertension. Estimates from logistic regression models were adjusted for age, sex, and heart period (IBI). The number in the heading of the graphs indicates the total number of cases (e.g., *n* = 1954) where participants fell into the criteria of clinically relevant elevated blood pressure versus normal. A total of 2907 participants were included in the regression model. Each line represents the logistic regression model with the corresponding binary RMSSD cut off in milliseconds.

## 4. Discussion

These results provide the first evidence that a single value of RMSSD may be associated with elevated risk across a range of established cardiovascular risk factors, including inflammatory markers, blood lipids, and glucose, as well as blood pressure. Consistent with the American Heart Association guidelines for the identification of novel biomarkers, we report the results of a large observational study in which levels of standard risk factors were assessed and the odds of various values of HRV in predicting those with and without established clinical levels of a range of risk factors were evaluated [22]. Thus, the present study provides a proof of concept study (the first phase) in the progressive development of HRV as a low-cost, noninvasive biomarker for clinical use.

Consistent with prior research from our group and others, all linear regression models showed HRV to be significantly associated with each established risk factor [2,4,5,7,9]. For example, our group has shown that HRV was independently associated with fasting glucose, HbA_1c_, white blood count, and C-reactive protein (CRP), even after accounting for activity of the sympathetic nervous system as indexed by overnight urinary norepinephrine [34,35]. Relatedly, we have shown that HRV was more significantly associated with a measure of self-rated health than a range of other biomarkers, including measures of glycemic status, inflammation, lipids, and blood pressure (BP) [10]. Thus, when a clinician asks a patient about their self-rated health, this information is more closely related to their level of HRV than their level of other common biomarkers.

Whereas all linear regression models were significant, in most cases a curvilinear model provided the best fit. This suggests that there may be a critical value beyond which the risk associated with a given value of HRV increases. This is a crucial point in the quest for clinical cut-points for HRV. Therefore, we next used adjusted logistic regression models and the associated forest plots of the odds ratios at various levels of HRV to aid our decision making.

Given the shared autonomic regulation of both blood pressure and HRV, we used BP as a benchmark against which HRV was examined to evaluate other biomarkers. Examination of the forest plot for the odds ratios of different values of HRV suggested a value of HRV at which the risk of blood pressure in the hypertensive range increased (see Figure 1). Using this value, we then examined the forest plots associated with the other risk factors to search for a value, or more precisely a narrow range of values, for which the odds of having values of that risk factor in the clinical range increased.

For some risk factors, such as blood pressure and HBA_1c_, there was a clear inflection point indicating a value of HRV beyond which the odds ratios increased greatly. However, for other risk factors, such as C-reactive protein (CRP) or fibrinogen there appeared to be a linear dose response relationship between values of HRV and the odds ratios associated with the risk factor. Despite this linear relationship for some biomarkers, clinical cut points are often suggested. For example, we have shown that the association between fasting glucose and HRV is linear across the clinical cut points for glycemic status, including across the normal range [26]. This is also consistent with recommendations that for some biomarkers, lower values even within the “normal” range are associated with reduced risk [36,37].

We investigated HRV values during both the daytime and the nighttime. In general, the daytime values were stronger predictors of clinical values than nighttime measures. However, the amount of variance accounted for was similar for daytime and nighttime values of HRV and ranged from approximately 1% for total cholesterol (TC) to approximately 30% for BP.

### Limitations

There are several limitations of the present study. First, the percentage of females in the sample was less than 20%. Nonetheless, there were nearly 2000 females (18%) in the study. However, studies with a larger percentage of females would be useful, especially to evaluate if evidence appears suggesting sex-specific values for HRV as a clinical biomarker. Second, the sample was primarily Caucasian. Thus, future research is needed to ascertain if the present results hold for other ethnic and racial groups. In particular, in a recent meta-analysis we showed that despite having greater cardiovascular disease risk, African Americans on average have been shown to have greater HRV [38]. While it is been repeatedly shown that not all risk factors have the same association with risk in differing populations, the importance of identifying the boundary conditions of all risk factors cannot be emphasized enough. Third, the current study was cross-sectional in nature.

Future work is needed to establish the prospective value of a cut point for RMSSD in relation to cardiovascular disease risk. We and others have shown that low HRV often precedes elevated risk factors. For example, we have shown that low HRV predicted higher C-reactive protein (CRP) four years into the future [8]. This type of prospective association is the second phase of the evaluation of novel biomarkers as stated by the American Heart Association guidelines and should follow the proof of concept in which the biomarker is shown to differ between those with and without the outcome, as demonstrated in the present study [22]. Further studies may evaluate the optimal recording length that incorporates the most prognostic value (e.g., resting short term recordings, night or daytime or long-term (i.e., 24h) recordings).

## 5. Conclusions

This is the first study to attempt to identify a value of HRV that might be used in a clinical setting to identify persons at risk for across a range of cardiovascular disease risk factors. The present proof of concept study demonstrated that a measure of HRV can be used to discriminate between persons with and without clinical values on a broad range of established risk factors. Clearly, however, additional research is needed, consistent with the AHA guidelines, to further validate clinical cut-off values for HRV.

## Figures and Tables

**Table 1:** Classification of biomarkers into (clinical) categories.

	Variable	Condition	Normal	Preclinic	Clinic
*Glycemic Status*	FPG (mg/dL)		80–100	100–126	≥126
	HbA_1c_ (%)		4–6	6–6.5	≥6.5
*Inflammation Status*	CRP (mg/L)		<1	1–3	3–5
	WBC (μL)		<p33	≥p33–p66	≥p66
*Lipid Status*	LDL (mg/dL)		<160	≥160	
	HDL (mg/dL)	females	≥65	45 – 65	<45
	HDL (mg/dL)	males	≥55	35 – 55	<35
	TC (mg/dL)	age < 20	<170	-	≥170
	TC (mg/dL)	age 20–29	<200	-	≥200
	TC (mg/dL)	age 30–39	<220	-	≥220
	TC (mg/dL)	age 40+	<240	-	≥240
	TRIG (mg/dL)		<200	-	≥200
*Blood Pressure*	Sys BP (mmHg)	In combination with Dia BP	<120	≥120–140	≥140
	Dia BP (mmHg)	In combination with Sys BP	<90	<90	≥90

FPG: Fasting plasma glucose; HbA*_1c_*: Glycosylated hemoglobin (A1c); CRP: C-reactive protein (high sensitive); WBC: White blood count; LDL: Low-density lipoprotein; HDL: High-density lipoprotein; TC: Total cholesterol; TRIG: Triglyceride; SysBP: Systolic blood pressure; Dia BP: Diastolic blood pressure; p33 / p66: 33. percentile or 66. percentile respectively.

**Table 2:** Classification of biomarkers into (clinical) categories.

					Cutoff (%)		
	Variable	*n*	Mean	SD	Normal	Preclinical	Clinical
*Glycemic Status*	FPG (mg/dL)	9550	87.1	12	92.50%	6.48%	1.02%
	HbA_1c_ (%)	9550	5.4	0.5	74.36%	24.10%	1.54%
*Inflammation Status*	CRP (mg/L)	9550	1.6	3.4	61.91%	31.12%	6.97%
	WBC (μL)	9550	6.3	1.7	34.34%	35.69%	29.98%
*Lipid Status*	LDL (mg/dL)	9550	127.9	34	82.62%	17.38%	
	HDL (mg/dL)	9550	56.0	14.1	43.51%	51.91%	4.59%
	TC (mg/dL)	9550	209	40.2	69.49%	30.51%	
	TRIG (mg/dL)	9550	121.2	72.6	88.31%	11.69%	
*Blood Pressure*	Sys BP (mmHg)	9550	136.6	13.8	10.71%	75.24%	14.06%
	Dia BP (mmHg)	9550	78.6	11.3			
*Demographics*	Age	9550	41.7	11	-	-	-
	Male (*n*; %)	9550	7745	81%		-	-
*HRV*	RMSSD Daytime (msec)	9550	27.8	12	-	-	-
	RMSSD nighttime (msec)	8425	38.9	19	-	-	-

## Supplementary Materials

The following are available online at https://www.mdpi.com/2077-0383/8/11/1940/s1.

*Figures S1–S20*

Scatter plots showing the predicted values from simple linear regression models adjusted for age and sex (y-axis) and each RMSSD value of daytime or nighttime (x-axis). Grey x indicates measurements from females, black dots indicate measurements from male. A fractional polynomial function represents the average predicted value stratified by sex (dashed black line = males; solid grey line = women) and includes the 95% confidence interval (grey band). The red vertical line aids orientation for the 30 msec RMSSD value.

**Item Number**	**Filename**	**Description**
Figure S1	chol_predicted_day.png	Daytime
Figure S2	chol_predicted_night.png	Nighttime
Figure S3	crps_predicted_day.png	Daytime
Figure S4	crps_predicted_night.png	Nighttime
Figure S5	glucn_predicted_day.png	Daytime
Figure S6	glucn_predicted_night.png	Nighttime
Figure S7	hba1c_predicted_day.png	Daytime
Figure S8	hba1c_predicted_night.png	Nighttime
Figure S9	hdl_predicted_day.png	Daytime
Figure S10	hdl_predicted_night.png	Nighttime
Figure S11	ldllg_predicted_day.png	Daytime
Figure S12	ldllg_predicted_night.png	Nighttime
Figure S13	leuk_predicted_day.png	Daytime
Figure S14	leuk_predicted_night.png	Nighttime
Figure S15	rrdiam_predicted_day.png	Daytime
Figure S16	rrdiam_predicted_night.png	Nighttime
Figure S17	rrsysm_predicted_day.png	Daytime
Figure S18	rrsysm_predicted_night.png	Nighttime
Figure S19	trig_predicted_day.png	Daytime
Figure S20	trig_predicted_night.png	Nighttime

Scatter plots showing the actual values of each measure (y-axis) and each RMSSD value of daytime or nighttime (x-axis). Grey x indicates measurements from females, black dots indicate measurements from male. A fractional polynomial function represents the average predicted value stratified by sex (dashed black line = males; solid grey line = women) and includes the 95% confidence interval (grey band). The red vertical line aids orientation for the 30 msec RMSSD value.

*Figures S21–S40*

**Item Number**	**Filename**	**Description**
Figure S21	chol_actual_day.png	Daytime
Figure S22	chol_actual_night.png	Nighttime
Figure S23	crps_actual_day.png	Daytime
Figure S24	crps_actual_night.png	Nighttime
Figure S25	glucn_actual_day.png	Daytime
Figure S26	glucn_actual_night.png	Nighttime
Figure S27	hba1c_actual_day.png	Daytime
Figure S28	hba1c_actual_night.png	Nighttime
Figure S29	hdl_actual_day.png	Daytime
Figure S30	hdl_actual_night.png	Nighttime
Figure S31	ldllg_actual_day.png	Daytime
Figure S32	ldllg_actual_night.png	Nighttime
Figure S33	leuk_actual_day.png	Daytime
Figure S34	leuk_actual_night.png	Nighttime
Figure S35	rrdiam_actual_day.png	Daytime
Figure S36	rrdiam_actual_night.png	Nighttime
Figure S37	rrsysm_actual_day.png	Daytime
Figure S38	rrsysm_actual_night.png	Nighttime
Figure S39	trig_actual_day.png	Daytime
Figure S40	trig_actual_night.png	Nighttime

*Figures S41–S57*

Forest plots showing the odds ratios with according 95% confidence interval estimates for per RMSSD cut off (per 2 msec increment) for each risk factor. Odds ratios larger than 1 indicates an increased risk, e.g., for hypertension (HvsC_night_BP_120_90vs140_90.png). Estimates from logistic regression models adjusted for age, sex, and heart period (IBI). The number in the heading of the graphs indicate the total number of cases (e.g., in HvsC_night_BP_120_90vs140_90.png *n* = 1954 fell into the criteria of clinically relevant elevated blood pressure versus normal). A total of *n* = 2907 participants went into the regression model. Each line represents the logistic regression model with the according binary RMSSD cut off in milliseconds.

**Item Number**	**Filename**
*Figure 1*	*HvsC_day_BP_120_90vs140_90.png*
Figure S41	HvsC_day_CHOL_170plus_agedep.png
Figure S42	HvsC_day_CRPS_1vs3.png
Figure S43	HvsC_day_GLUC_100vs126.png
Figure S44	HvsC_day_HBA1C_6vs6point5.png
Figure S45	HvsC_day_HDL_55_65vs45_35.png
Figure S46	HvsC_day_LDL_160plus.png
Figure S47	HvsC_day_LEUK_33pc_vs66pc.png
Figure S48	HvsC_day_TRIG_200plus.png
Figure S49	HvsC_night_BP_120_90vs140_90.png
Figure S50	HvsC_night_CHOL_170plus_agedep.png
Figure S51	HvsC_night_CRPS_1vs3.png
Figure S52	HvsC_night_GLUC_100vs126.png
Figure S53	HvsC_night_HBA1C_6vs6point5.png
Figure S54	HvsC_night_HDL_55_65vs45_35.png
Figure S55	HvsC_night_LDL_160plus.png
Figure S56	HvsC_night_LEUK_33pc_vs66pc.png
Figure S57	HvsC_night_TRIG_200plus.png
