# Supplementary figures and images for: First Evaluation of an Index of Low Vagally-Mediated Heart Rate Variability as a Marker of Health Risks in Human Adults: Proof of Concept
Source: J Clin Med. 2019 Nov 11;8(11):1940. doi: 10.3390/jcm8111940 (PMC6912519; doi:10.3390/jcm8111940)

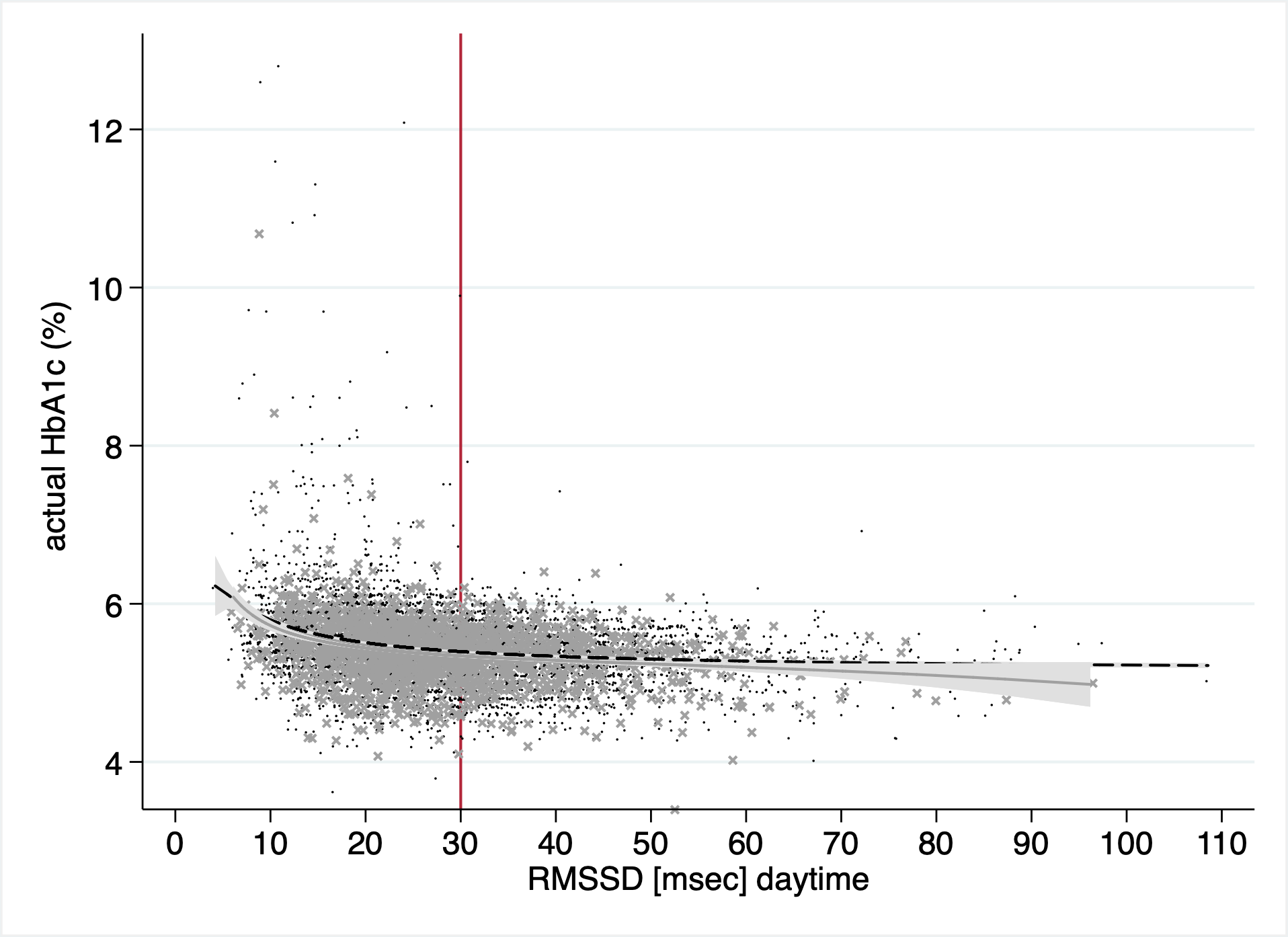

Supplement: Supplementary file 1 [file jcm-08-01940-s001.zip › supplements jcm_617360/hba1c_actual_day.png]

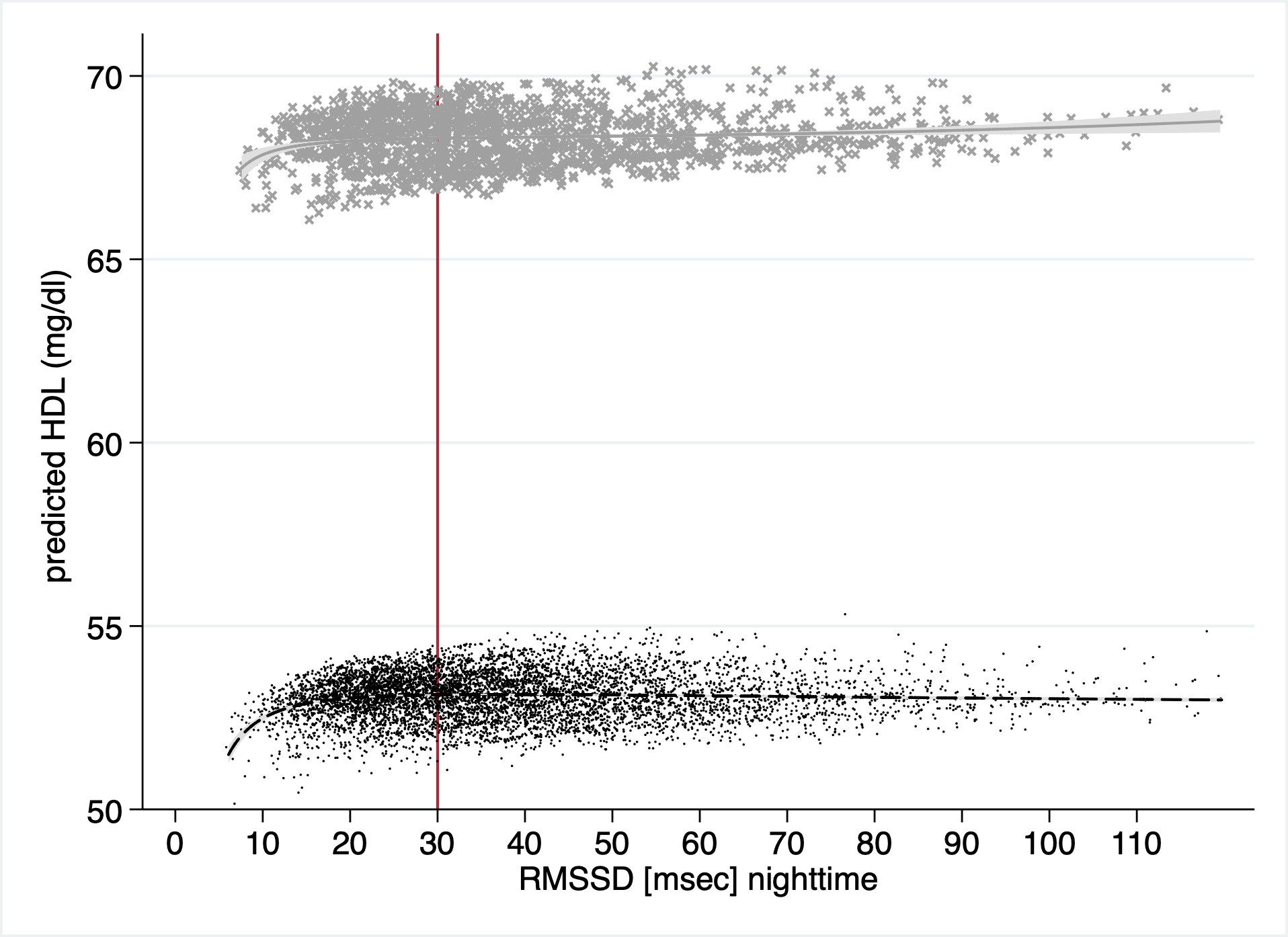

Supplement: Supplementary file 1 [file jcm-08-01940-s001.zip › supplements jcm_617360/hdl_predicted_night.png]

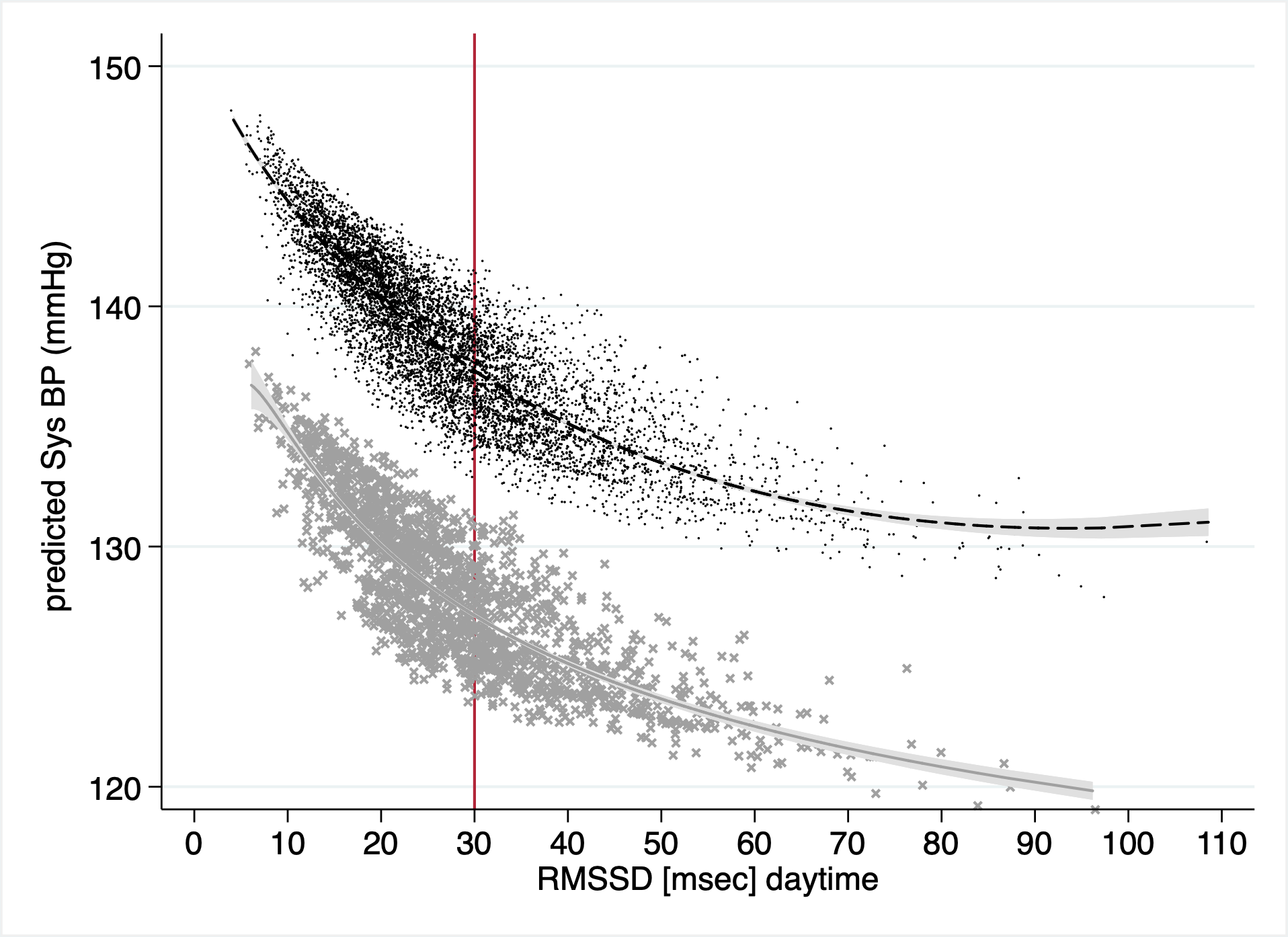

Supplement: Supplementary file 1 [file jcm-08-01940-s001.zip › supplements jcm_617360/rrsysm_predicted_day.png]

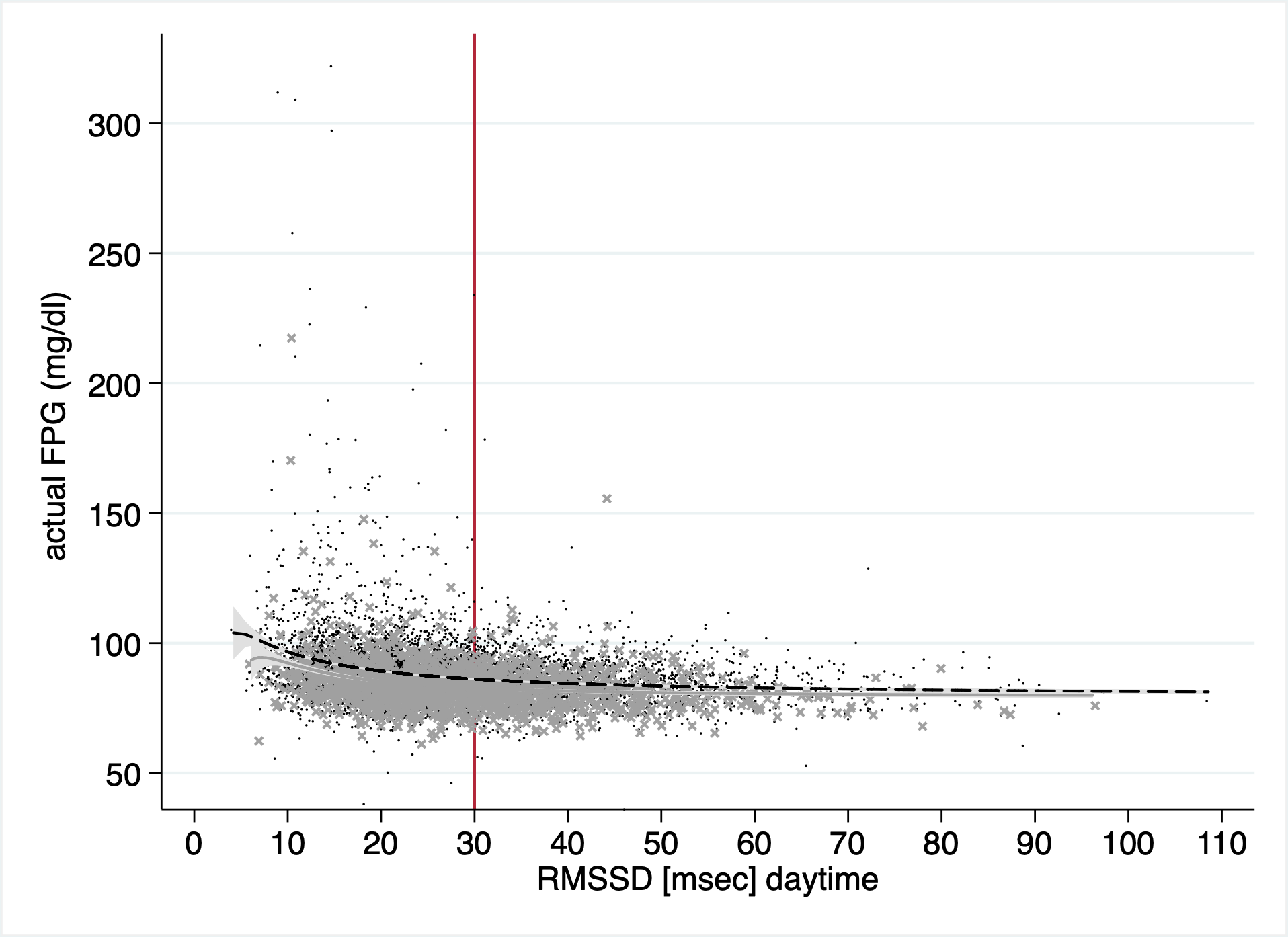

Supplement: Supplementary file 1 [file jcm-08-01940-s001.zip › supplements jcm_617360/glucn_actual_day.png]

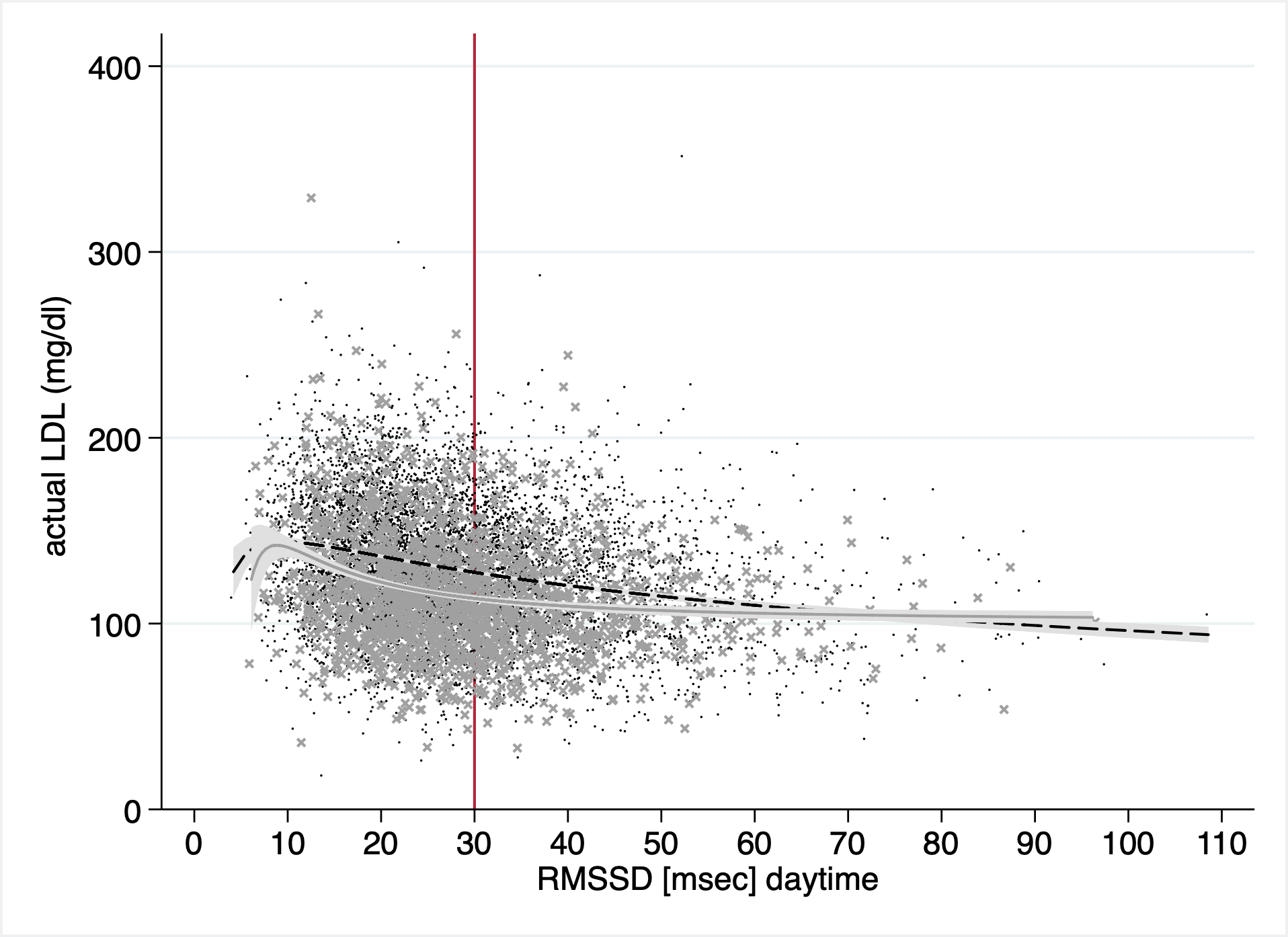

Supplement: Supplementary file 1 [file jcm-08-01940-s001.zip › supplements jcm_617360/ldllg_actual_day.png]

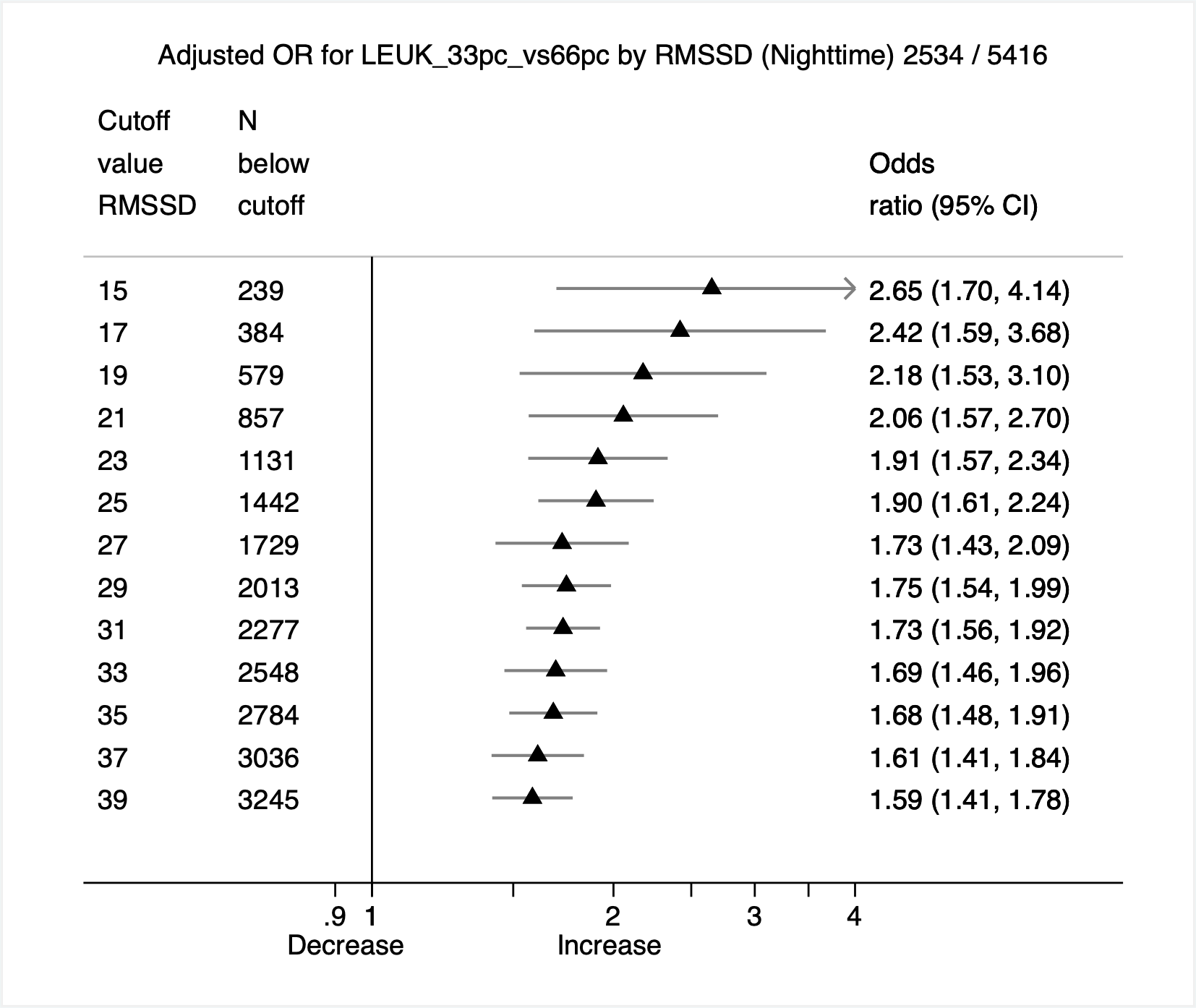

Supplement: Supplementary file 1 [file jcm-08-01940-s001.zip › supplements jcm_617360/HvsC_night_LEUK_33pc_vs66pc.png]

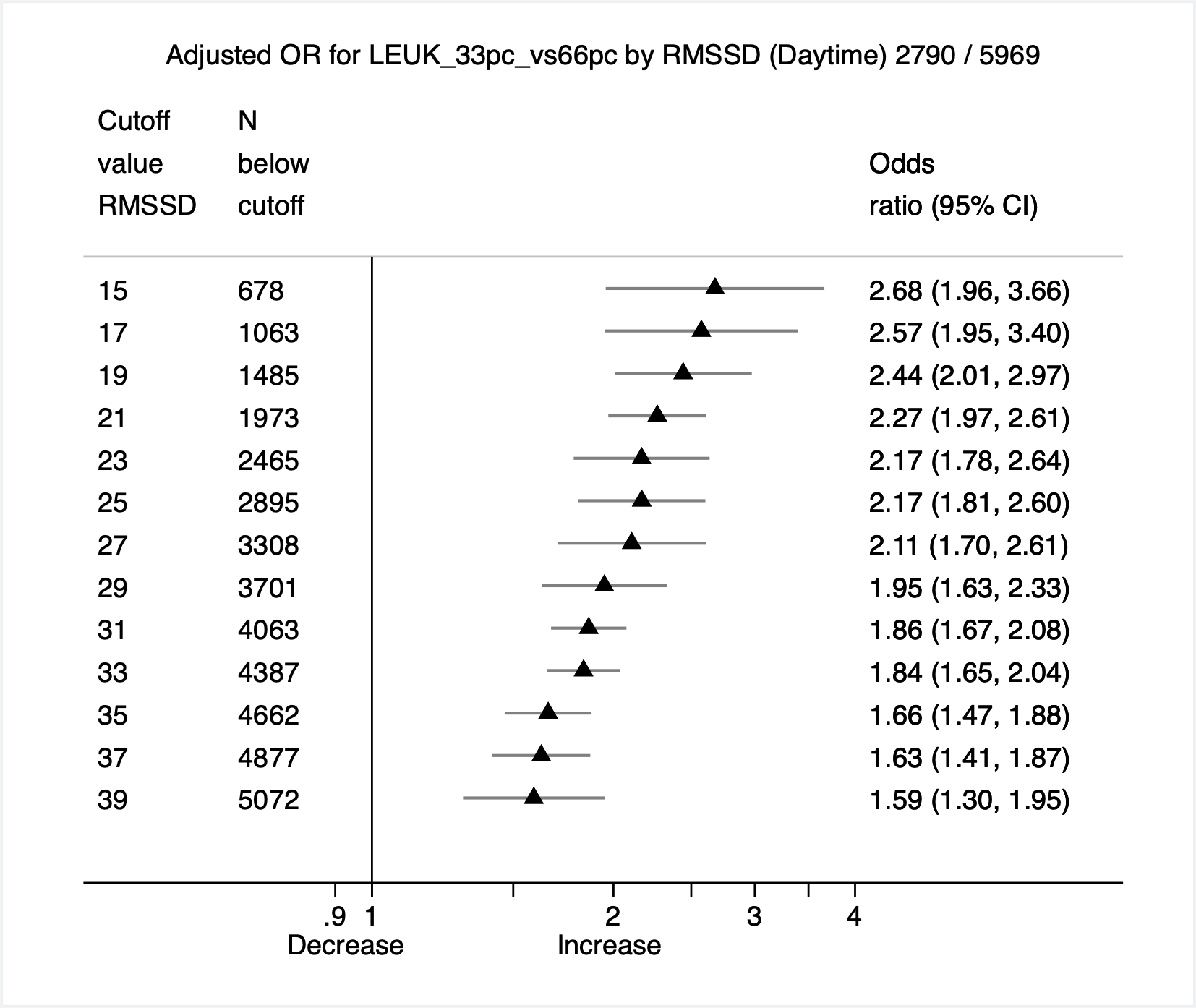

Supplement: Supplementary file 1 [file jcm-08-01940-s001.zip › supplements jcm_617360/HvsC_day_LEUK_33pc_vs66pc.png]

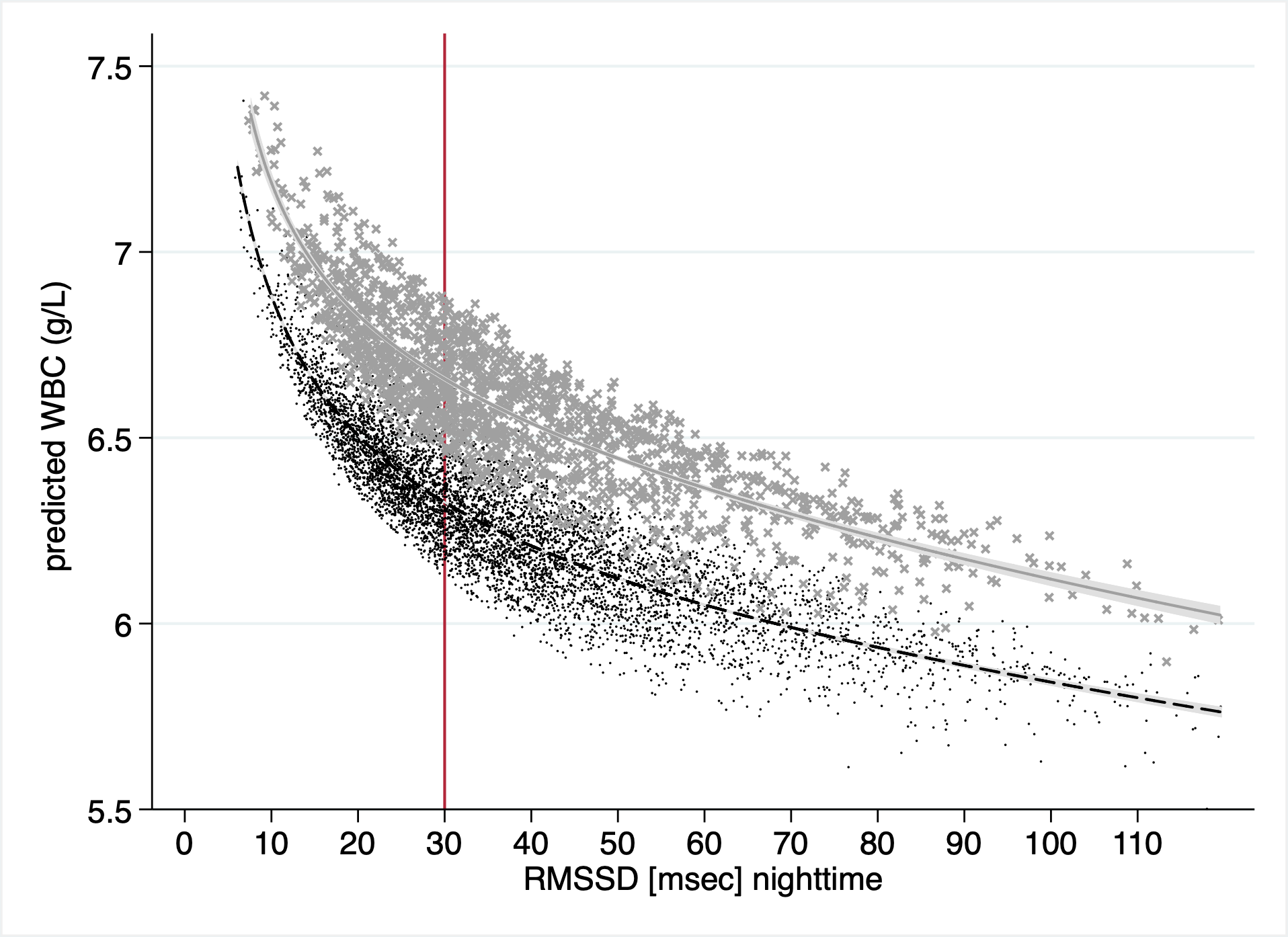

Supplement: Supplementary file 1 [file jcm-08-01940-s001.zip › supplements jcm_617360/leuk_predicted_night.png]

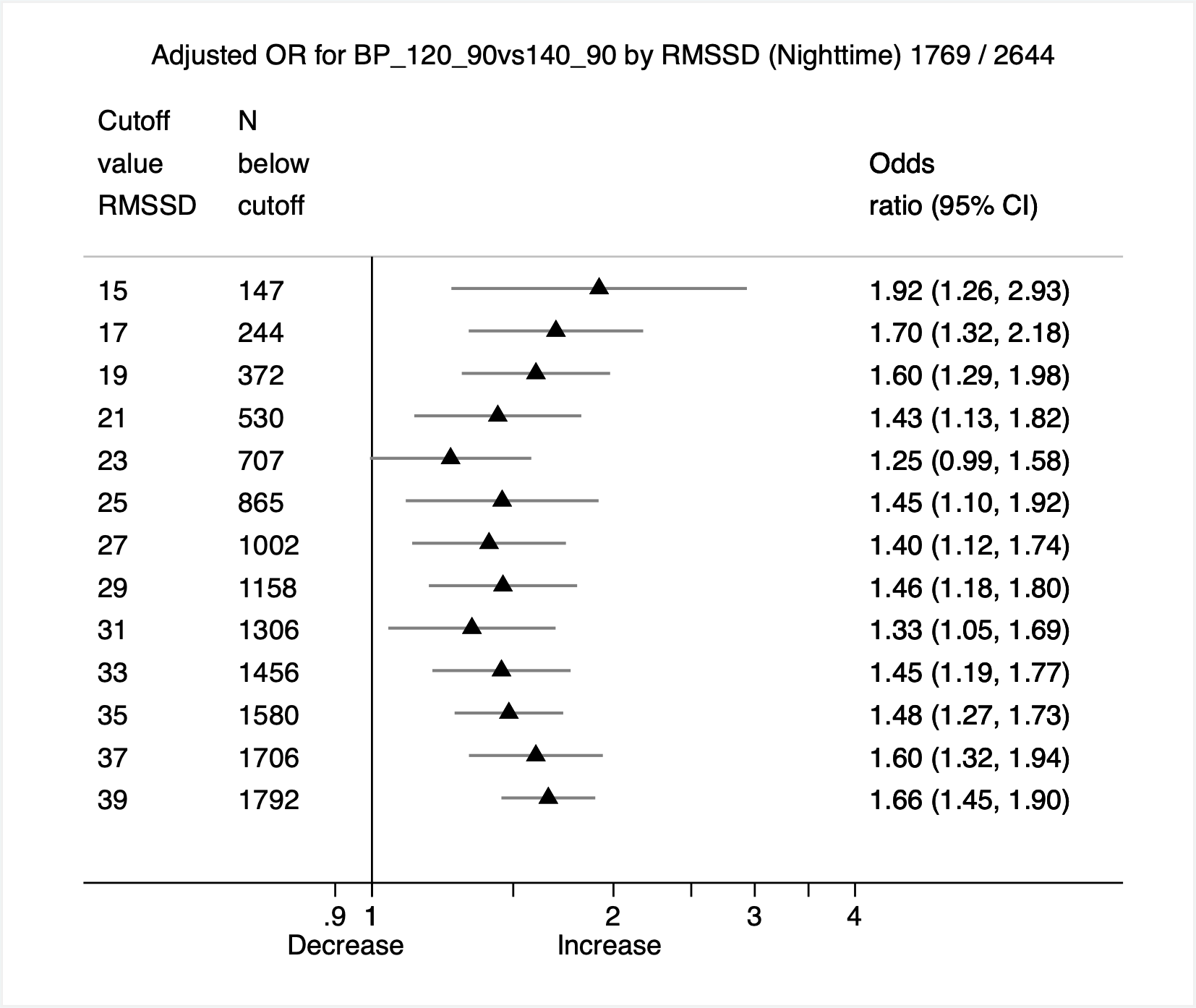

Supplement: Supplementary file 1 [file jcm-08-01940-s001.zip › supplements jcm_617360/HvsC_night_BP_120_90vs140_90.png]

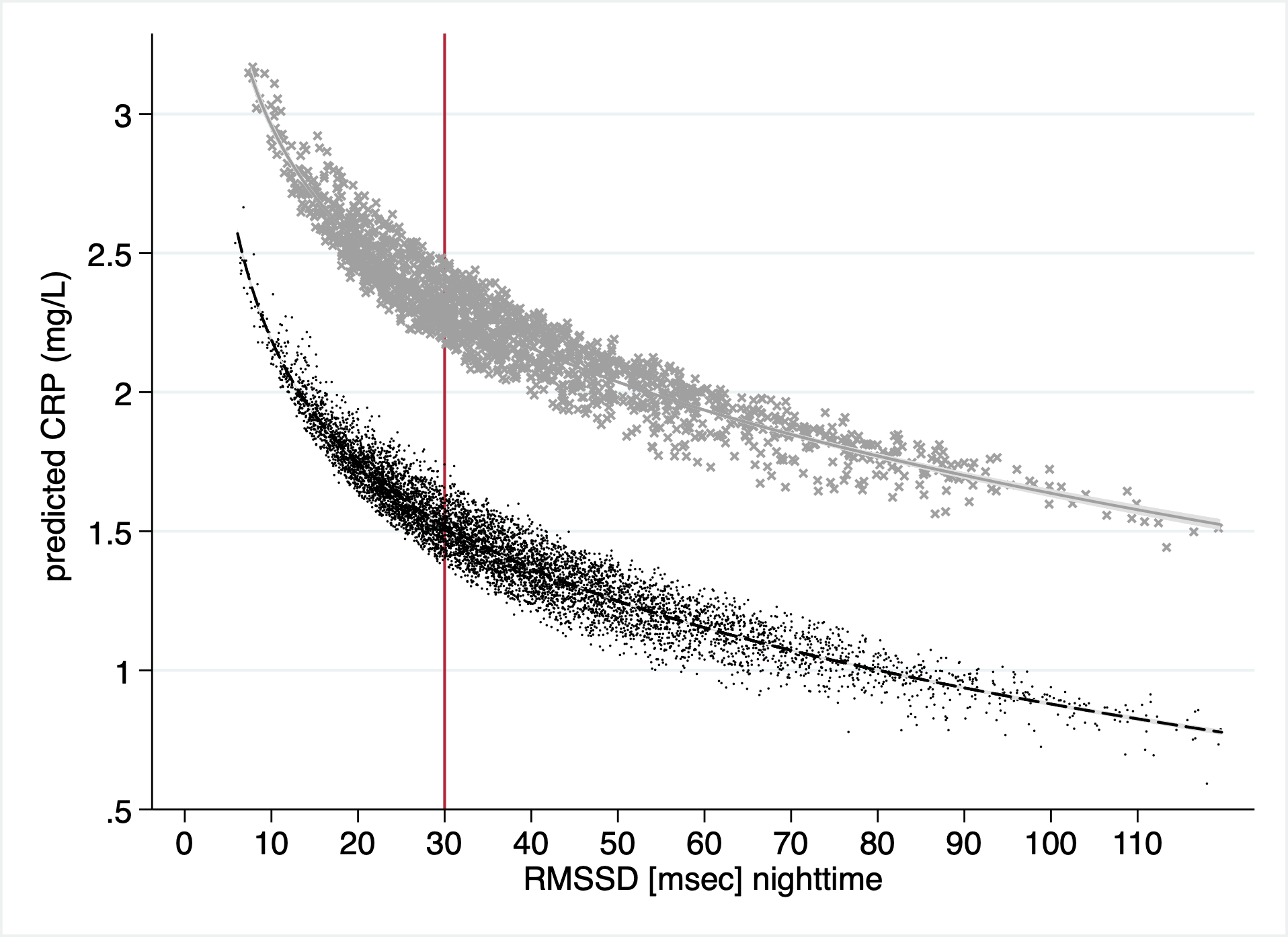

Supplement: Supplementary file 1 [file jcm-08-01940-s001.zip › supplements jcm_617360/crps_predicted_night.png]

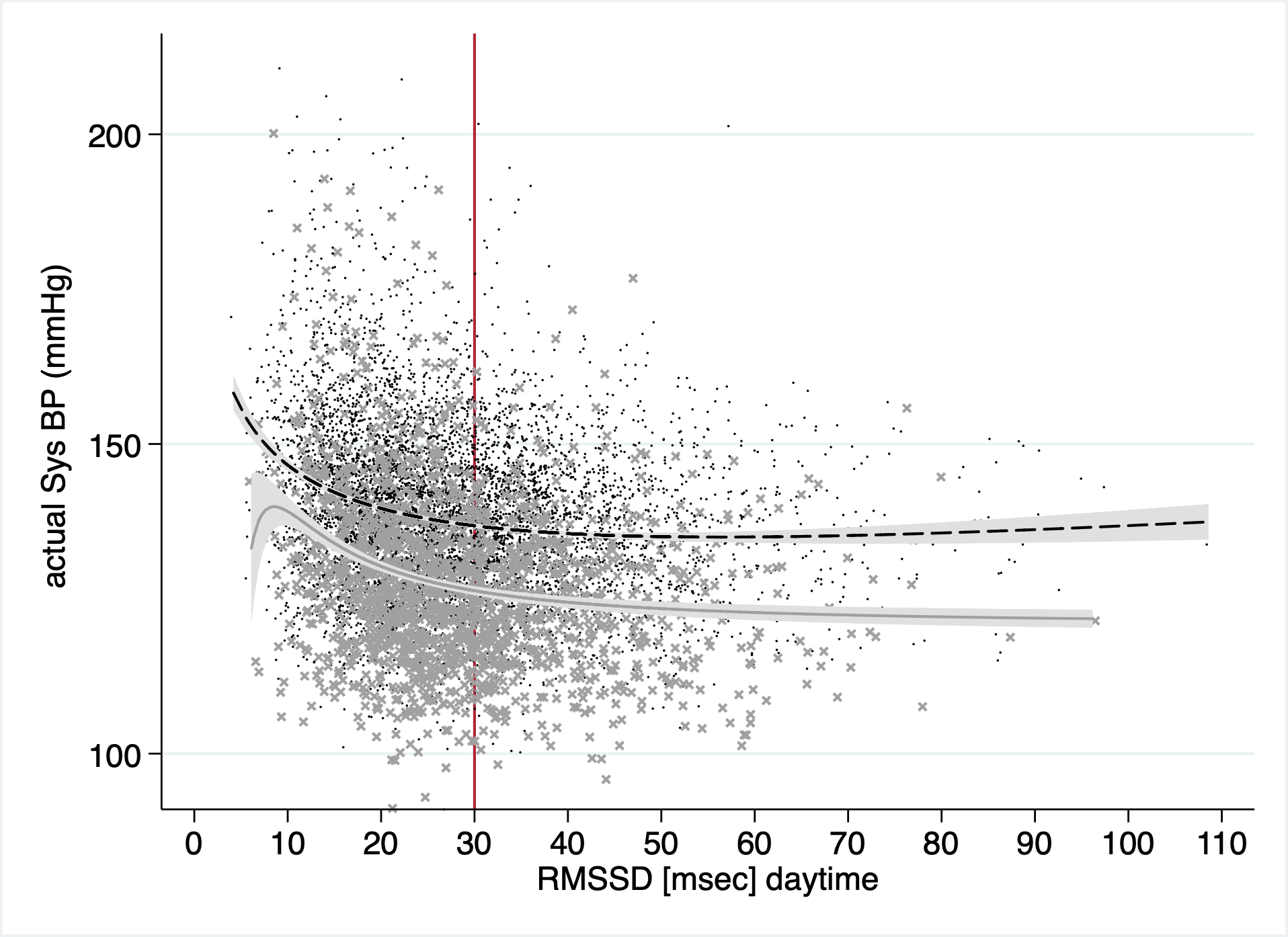

Supplement: Supplementary file 1 [file jcm-08-01940-s001.zip › supplements jcm_617360/rrsysm_actual_day.png]

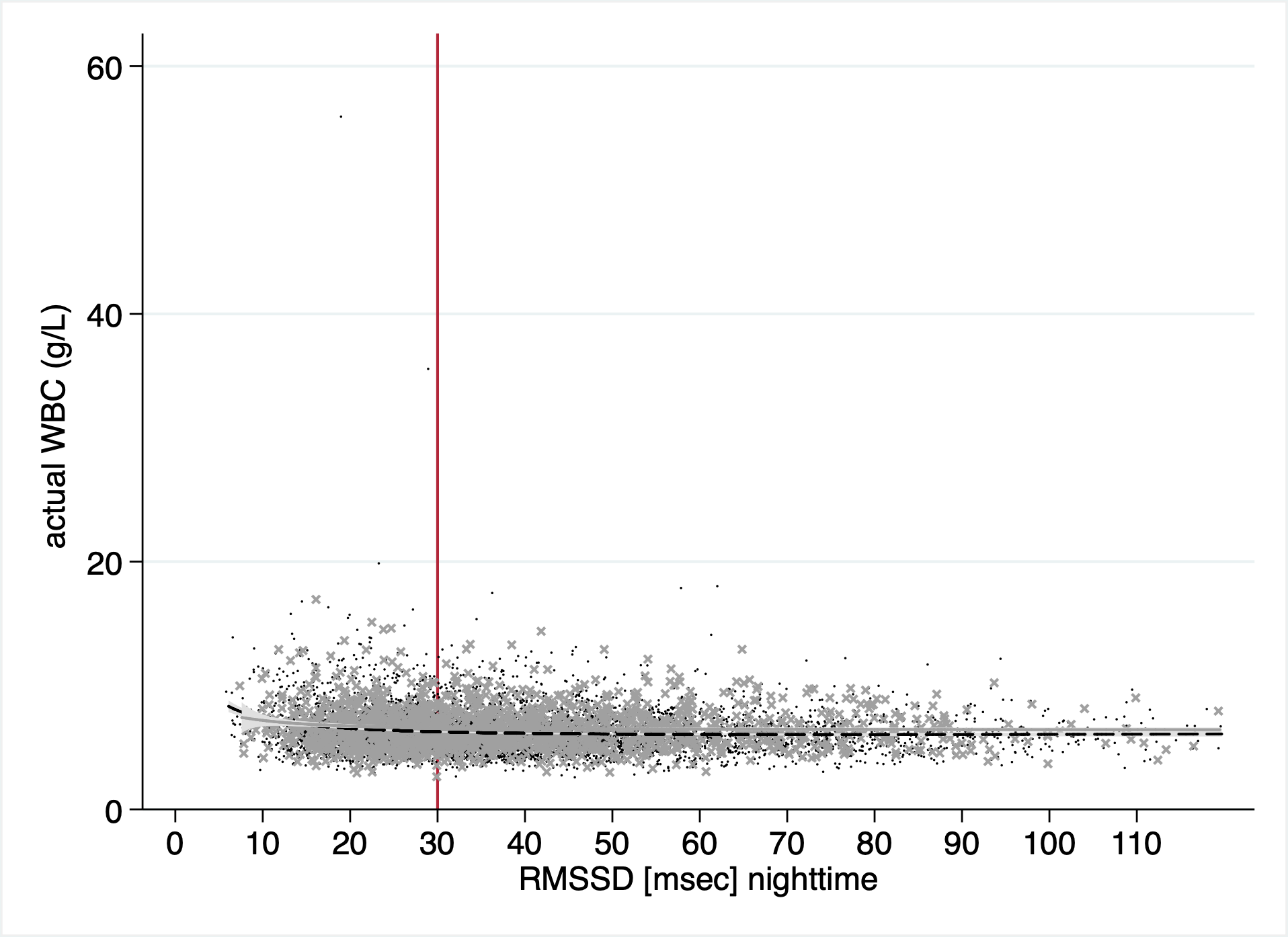

Supplement: Supplementary file 1 [file jcm-08-01940-s001.zip › supplements jcm_617360/leuk_actual_night.png]

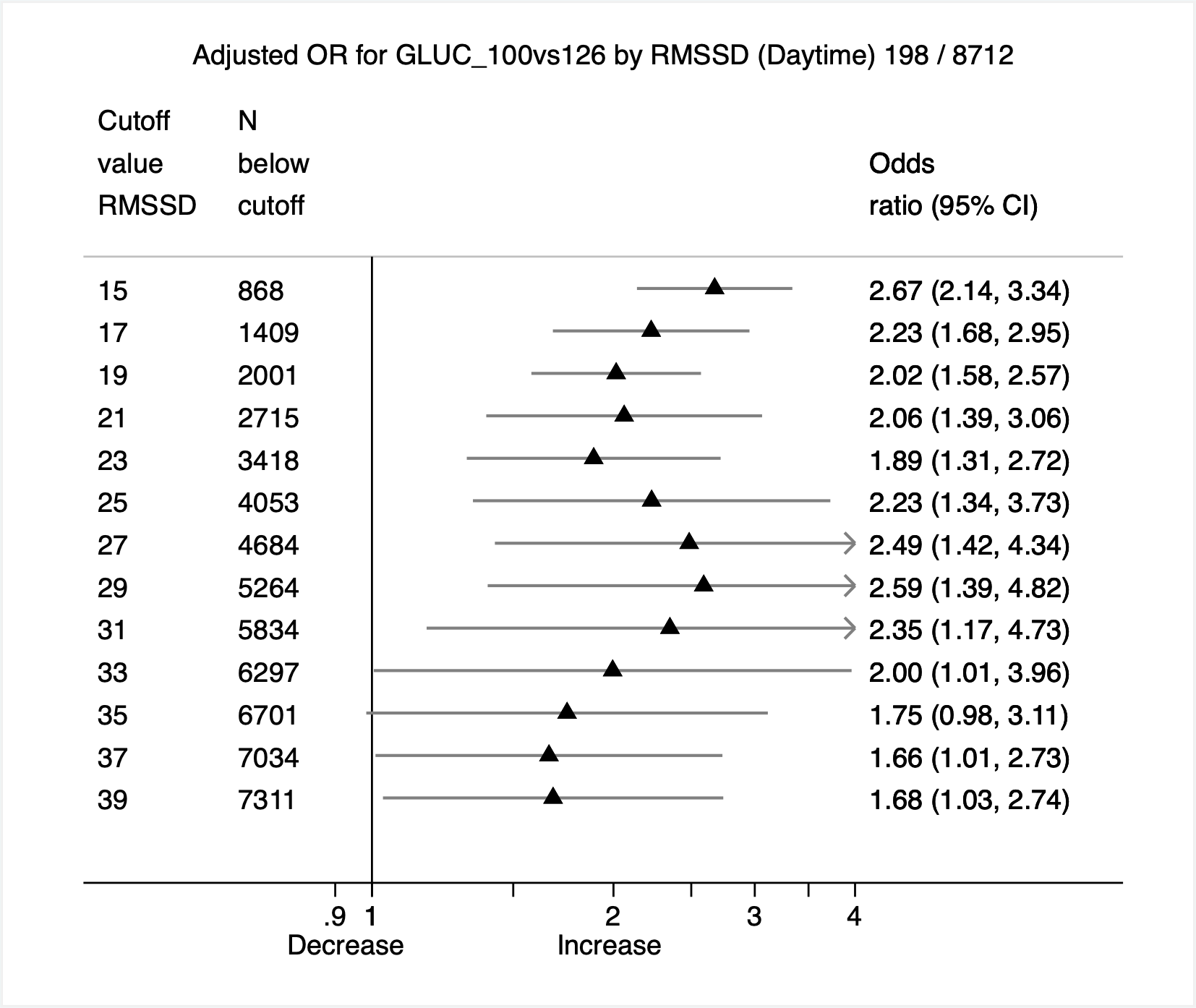

Supplement: Supplementary file 1 [file jcm-08-01940-s001.zip › supplements jcm_617360/HvsC_day_GLUC_100vs126.png]

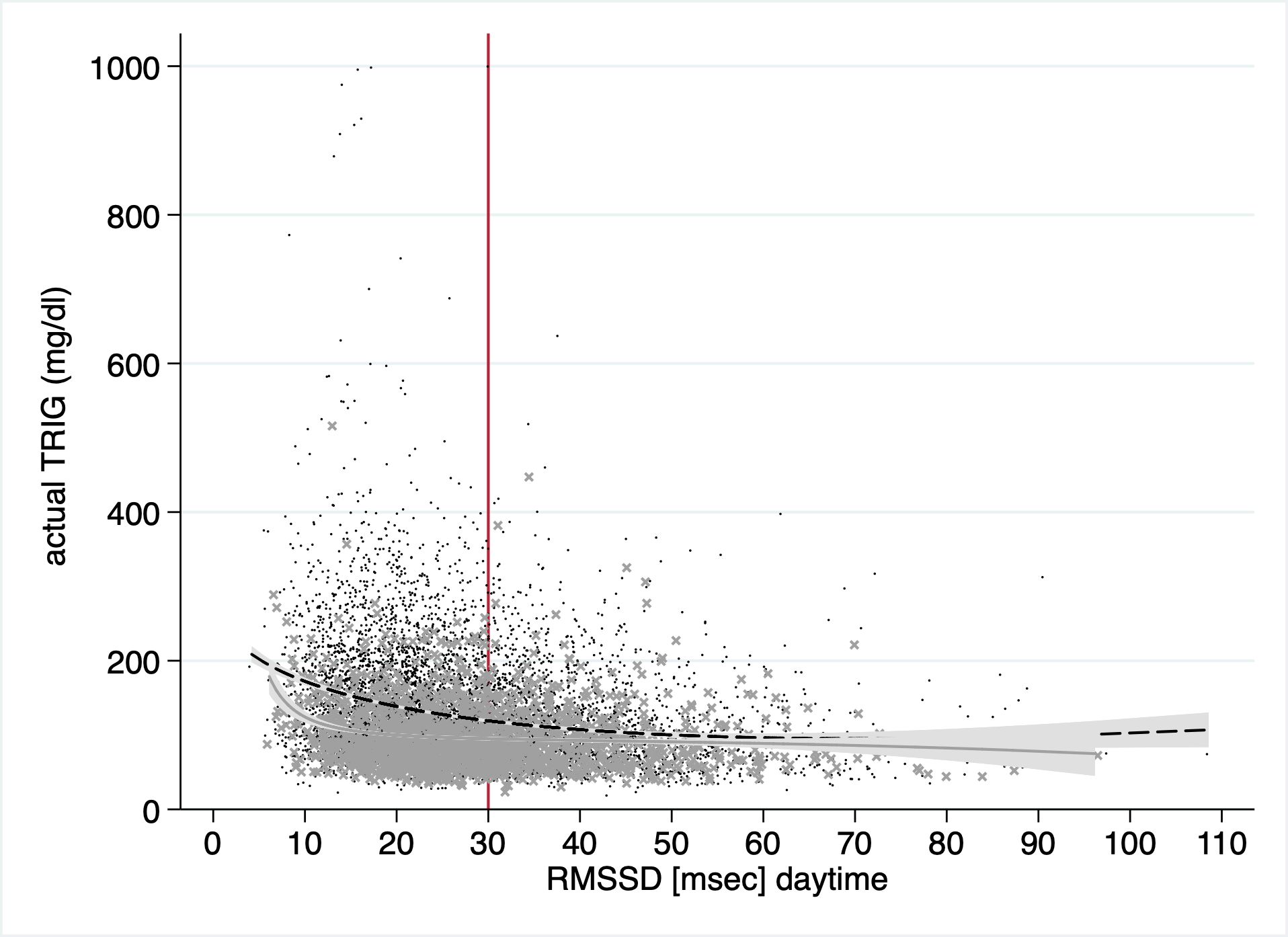

Supplement: Supplementary file 1 [file jcm-08-01940-s001.zip › supplements jcm_617360/trig_actual_day.png]

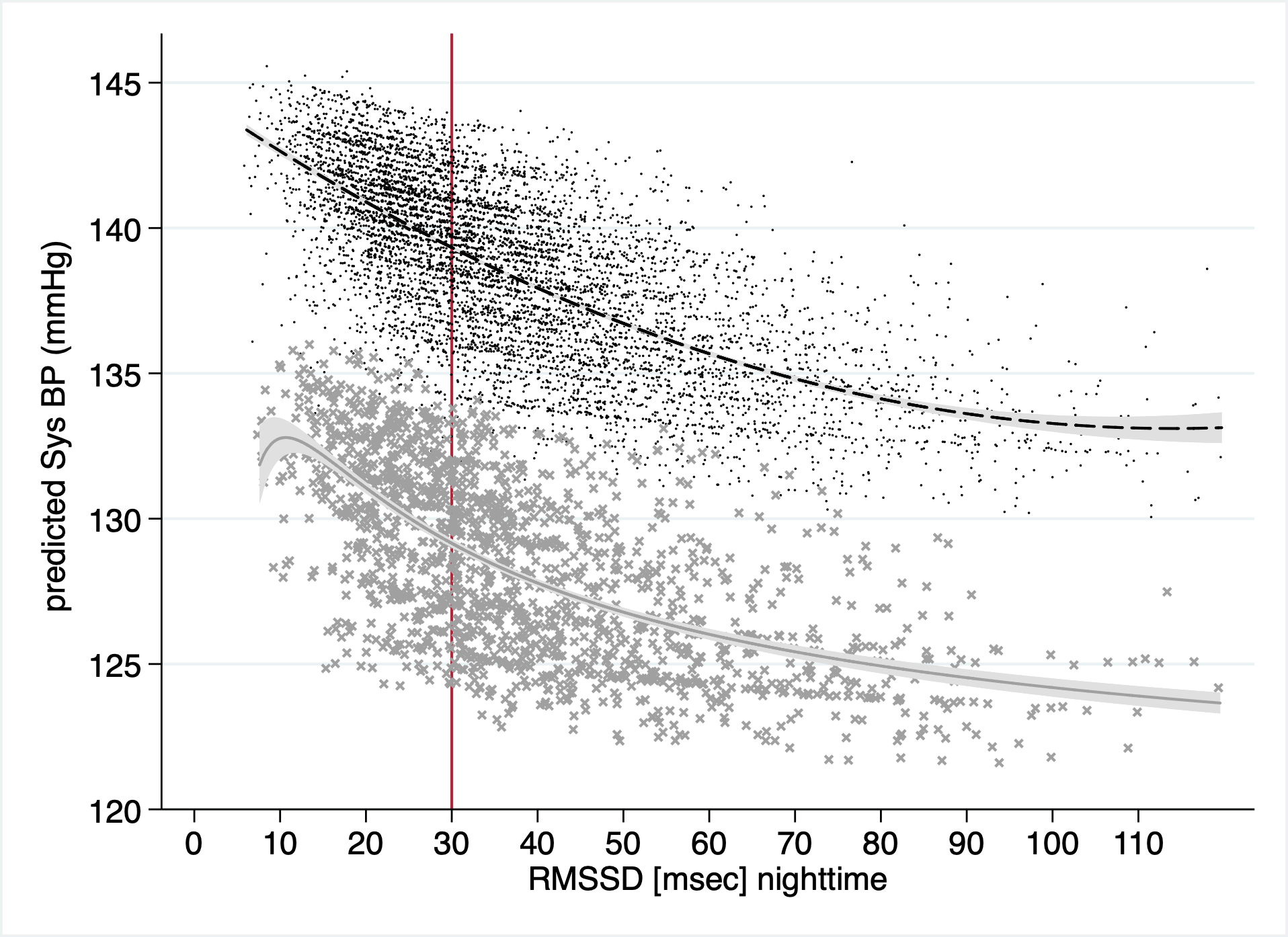

Supplement: Supplementary file 1 [file jcm-08-01940-s001.zip › supplements jcm_617360/rrsysm_predicted_night.png]

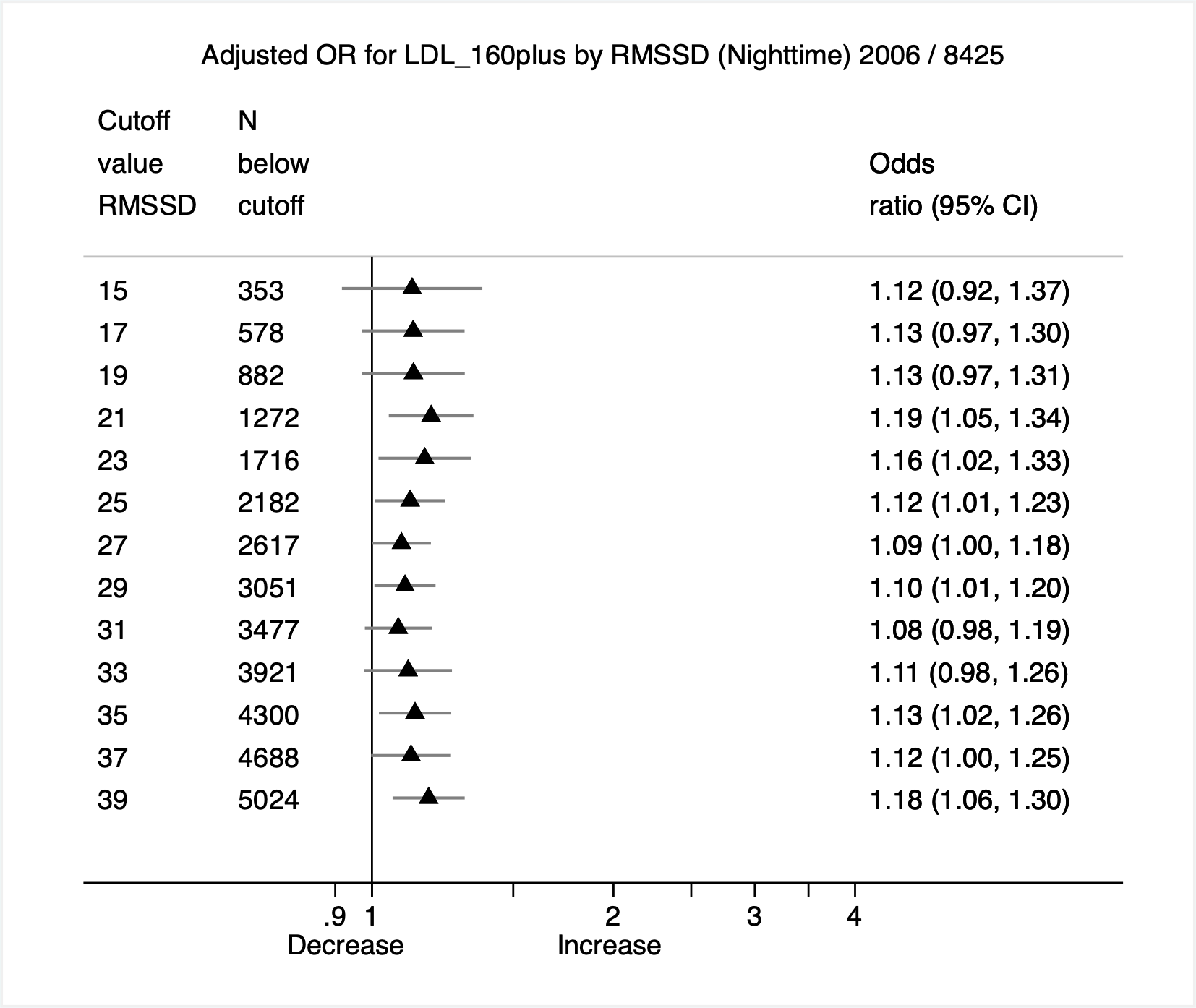

Supplement: Supplementary file 1 [file jcm-08-01940-s001.zip › supplements jcm_617360/HvsC_night_LDL_160plus.png]

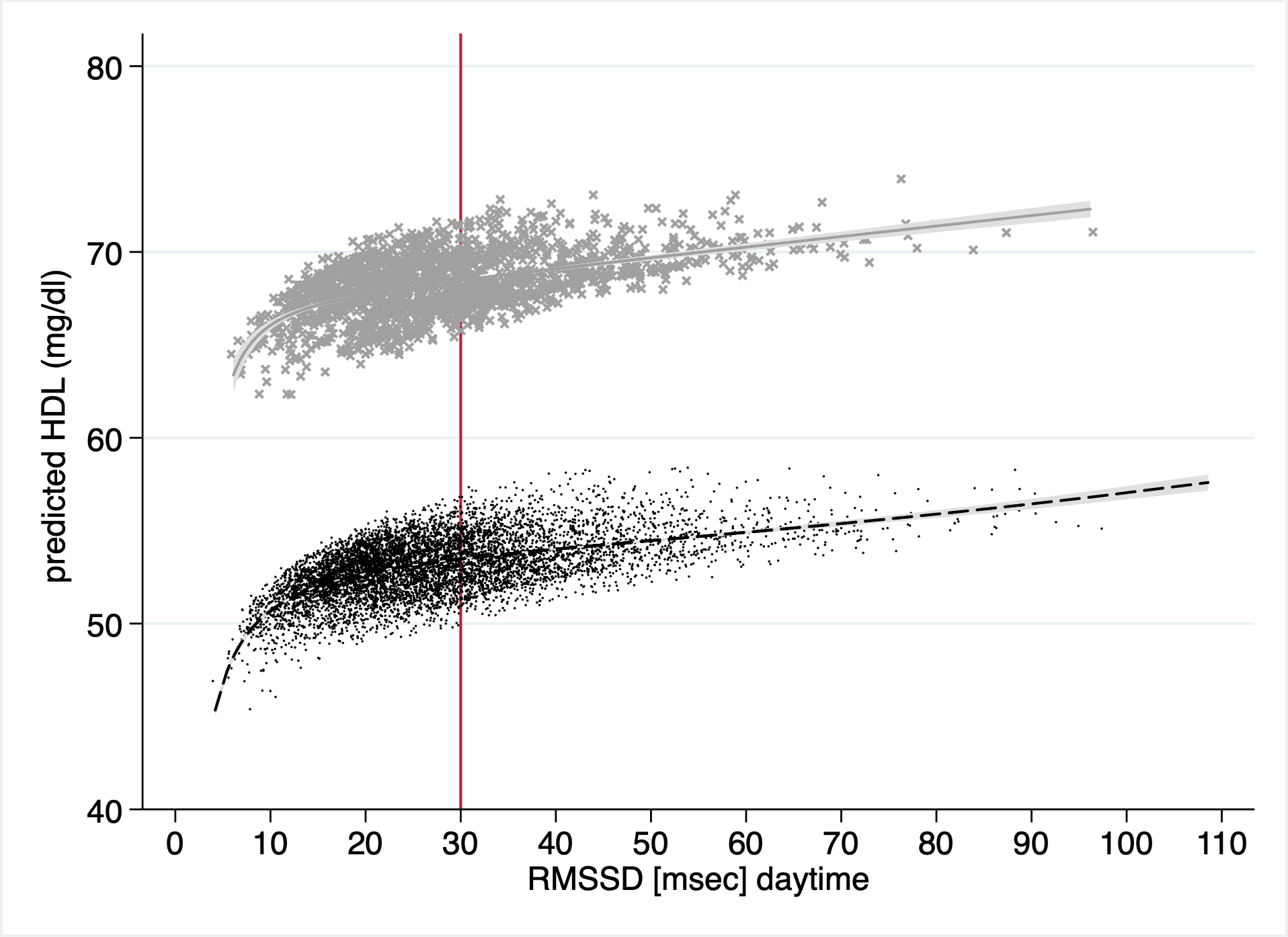

Supplement: Supplementary file 1 [file jcm-08-01940-s001.zip › supplements jcm_617360/hdl_predicted_day.png]

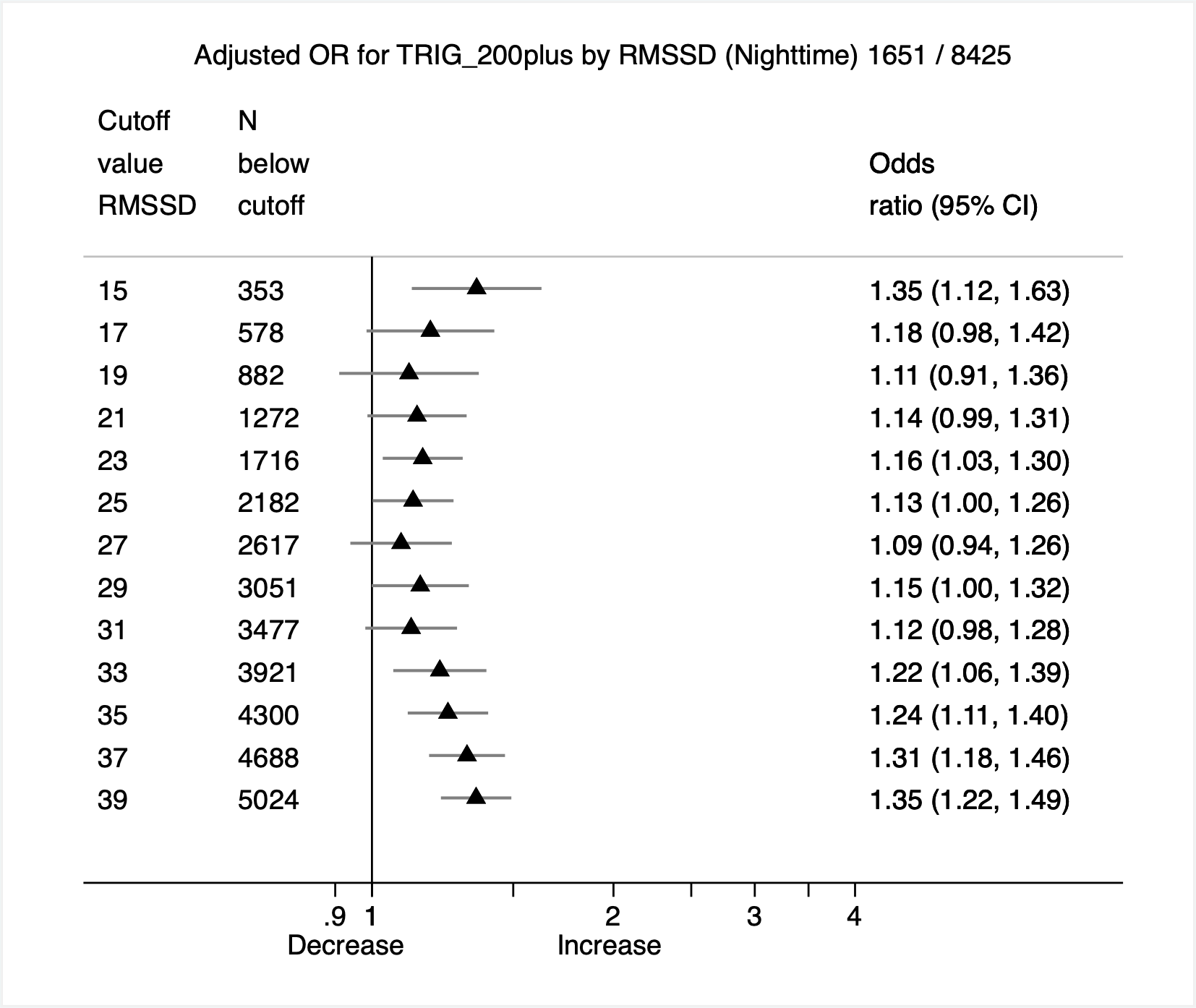

Supplement: Supplementary file 1 [file jcm-08-01940-s001.zip › supplements jcm_617360/HvsC_night_TRIG_200plus.png]

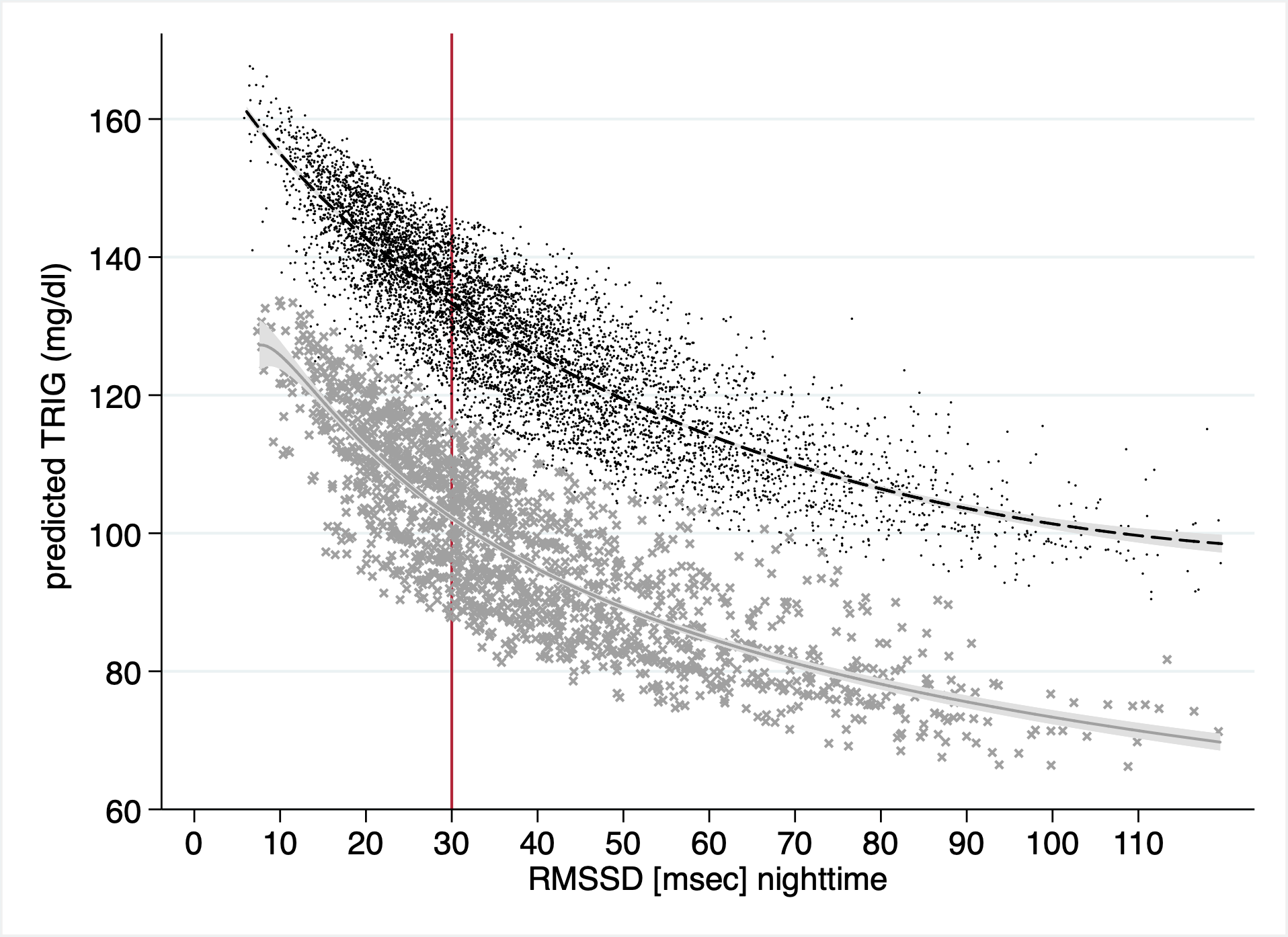

Supplement: Supplementary file 1 [file jcm-08-01940-s001.zip › supplements jcm_617360/trig_predicted_night.png]

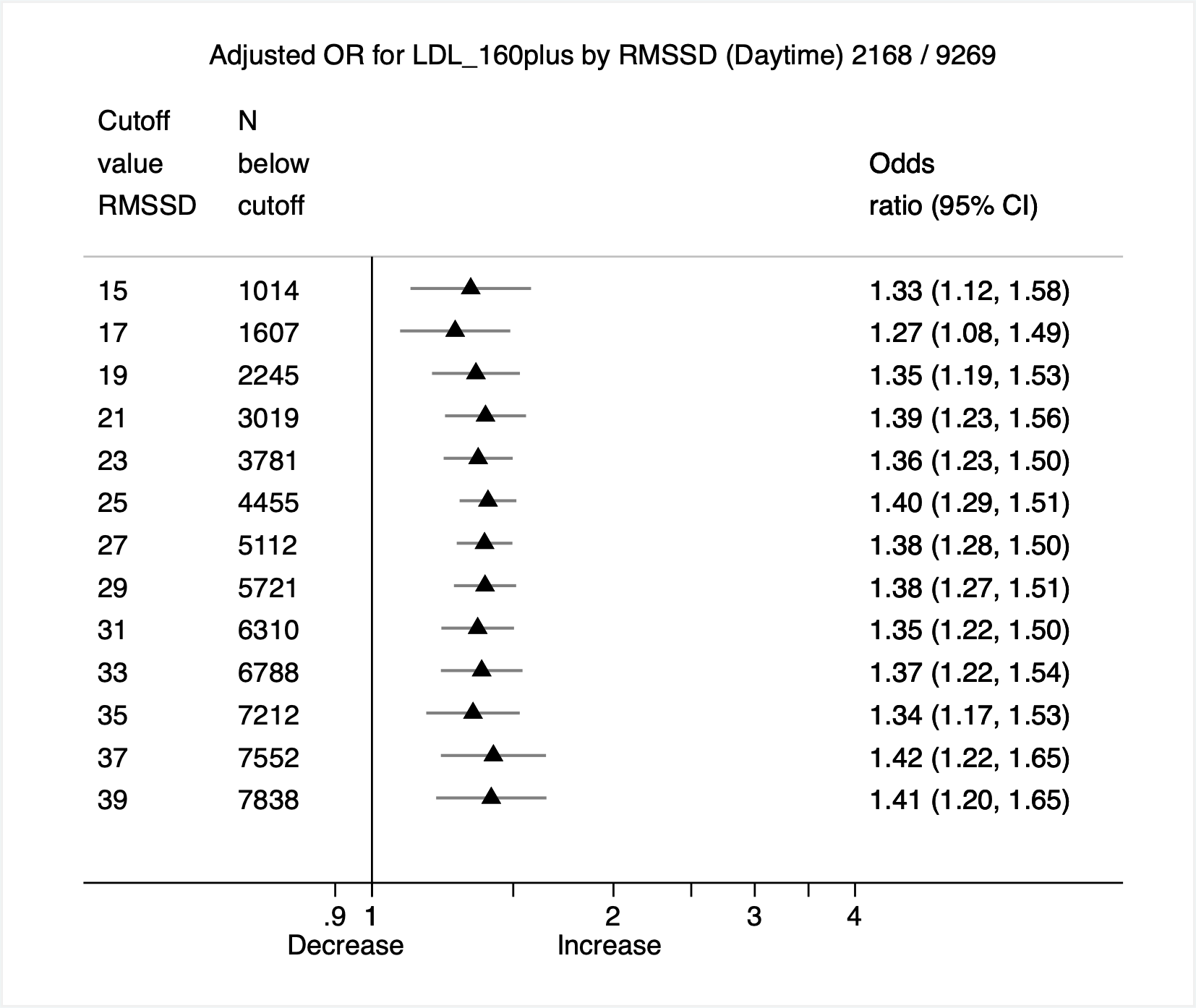

Supplement: Supplementary file 1 [file jcm-08-01940-s001.zip › supplements jcm_617360/HvsC_day_LDL_160plus.png]

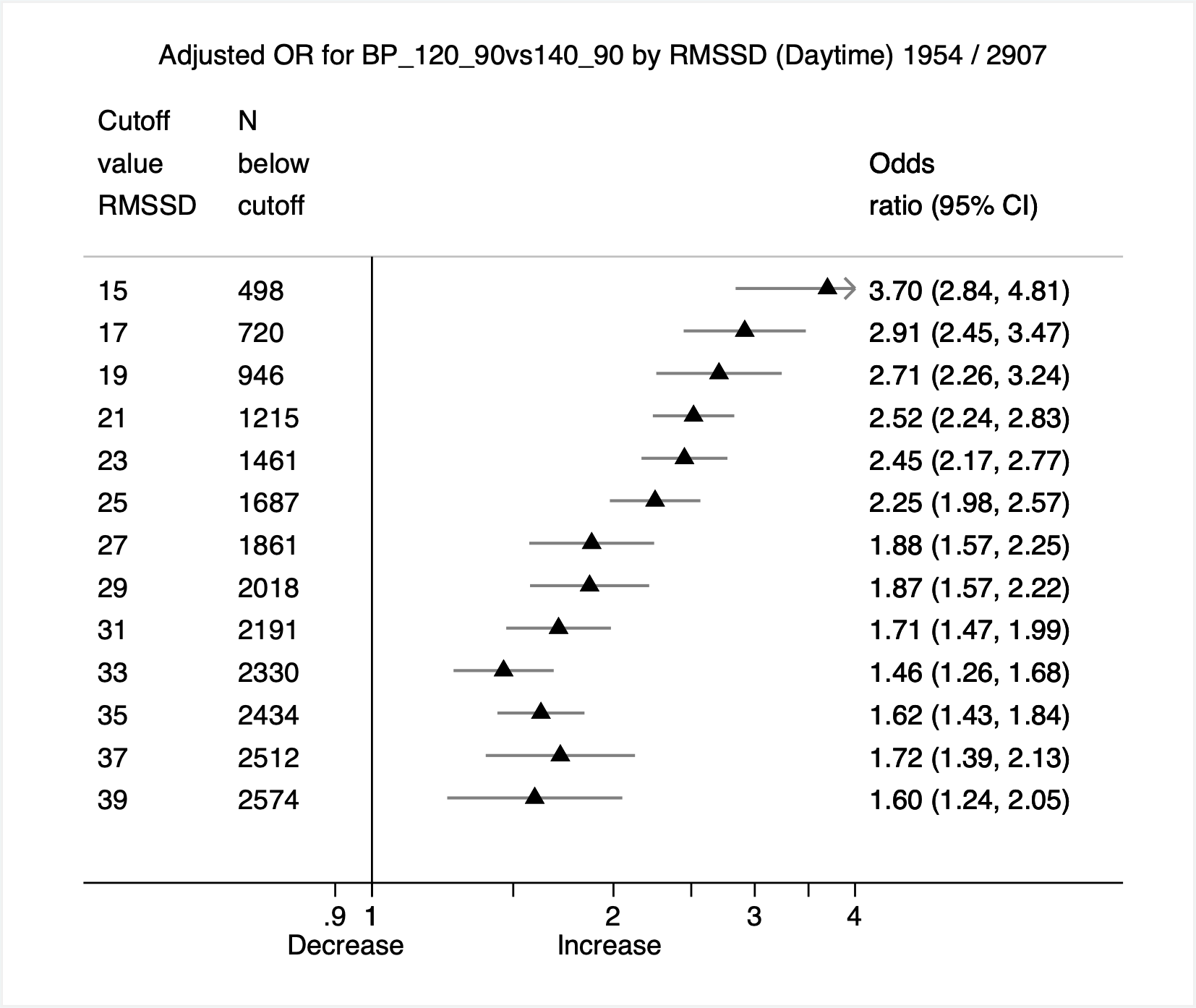

Supplement: Supplementary file 1 [file jcm-08-01940-s001.zip › supplements jcm_617360/HvsC_day_BP_120_90vs140_90.png]

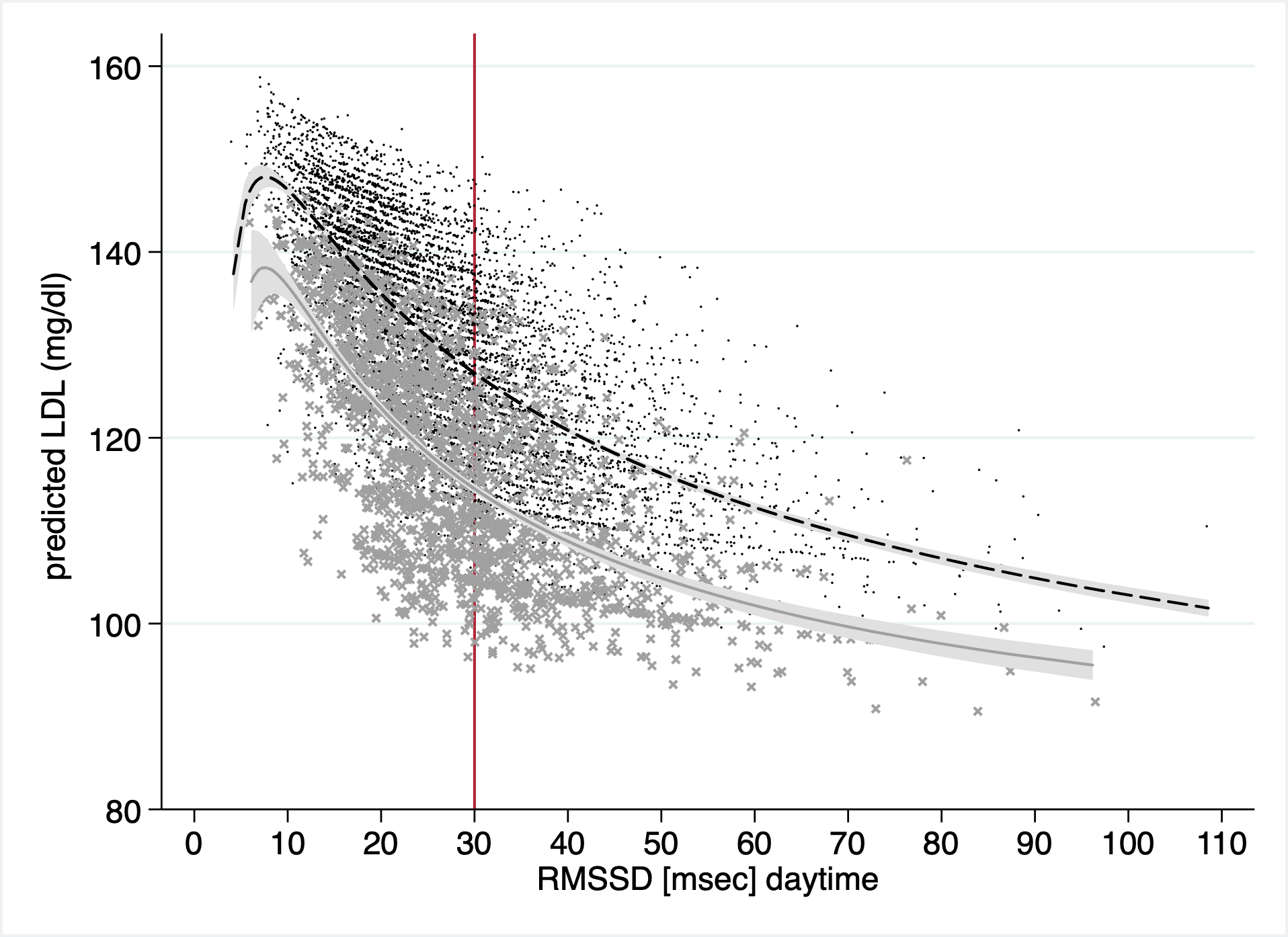

Supplement: Supplementary file 1 [file jcm-08-01940-s001.zip › supplements jcm_617360/ldllg_predicted_day.png]

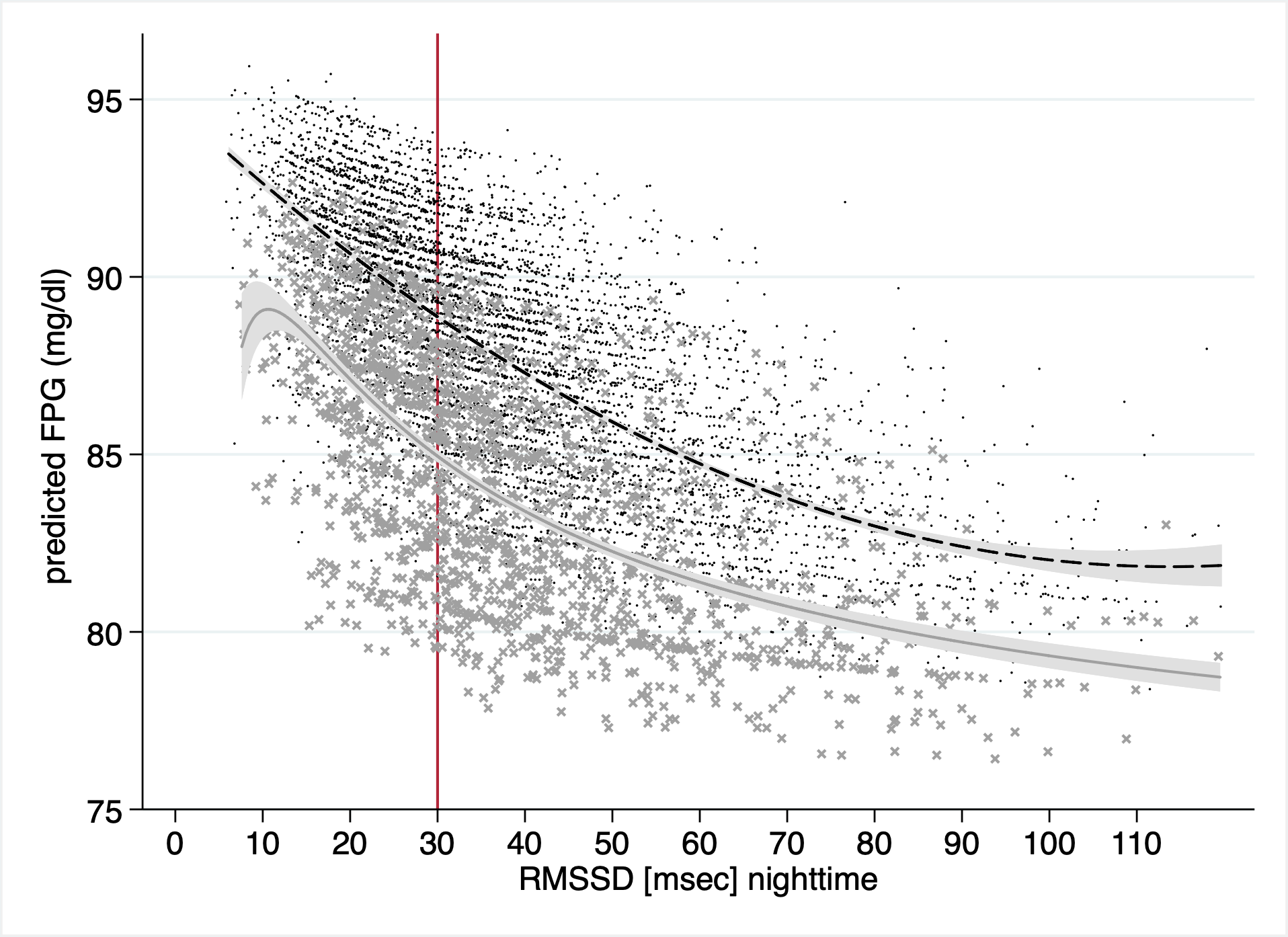

Supplement: Supplementary file 1 [file jcm-08-01940-s001.zip › supplements jcm_617360/glucn_predicted_night.png]

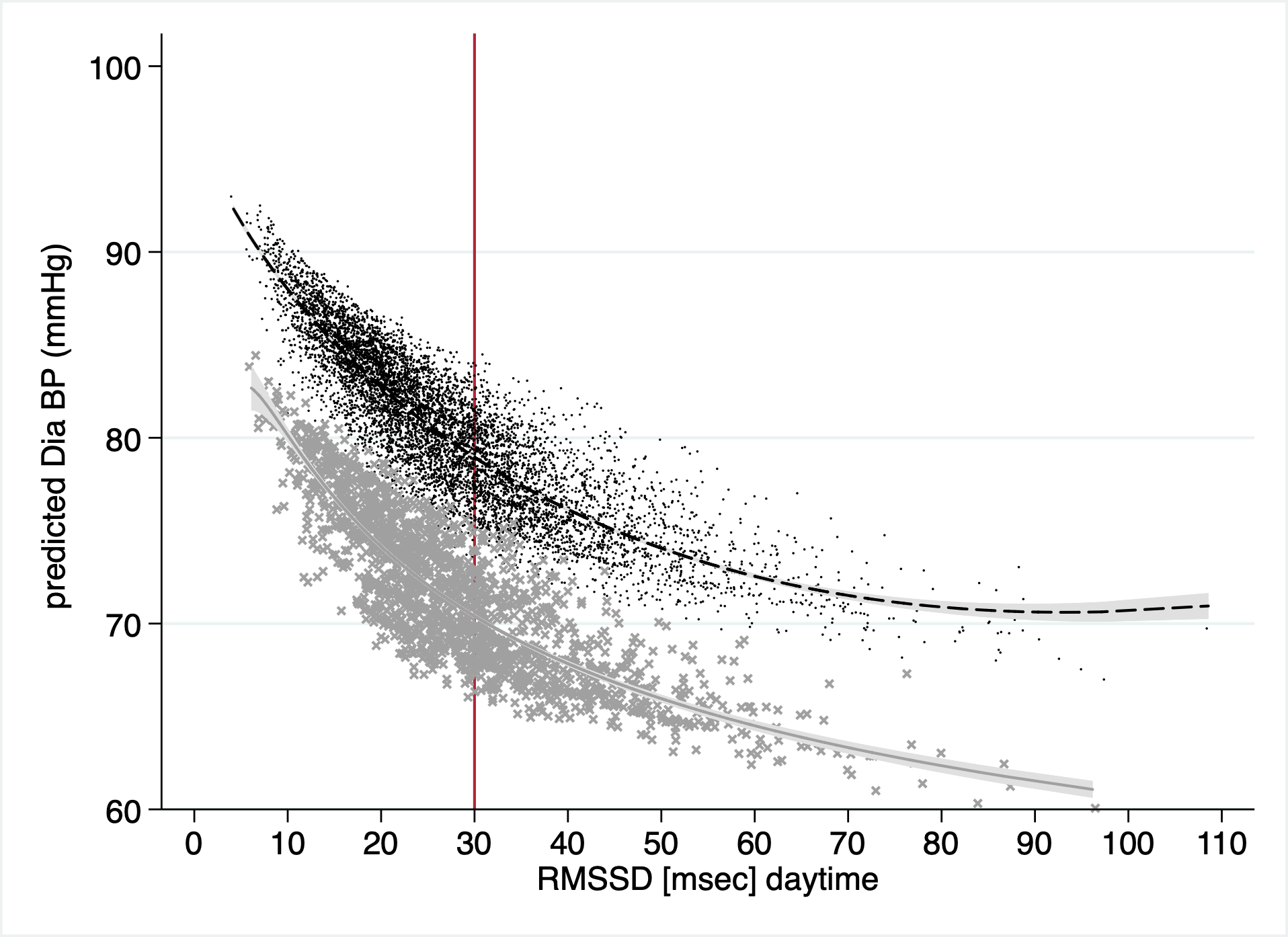

Supplement: Supplementary file 1 [file jcm-08-01940-s001.zip › supplements jcm_617360/rrdiam_predicted_day.png]

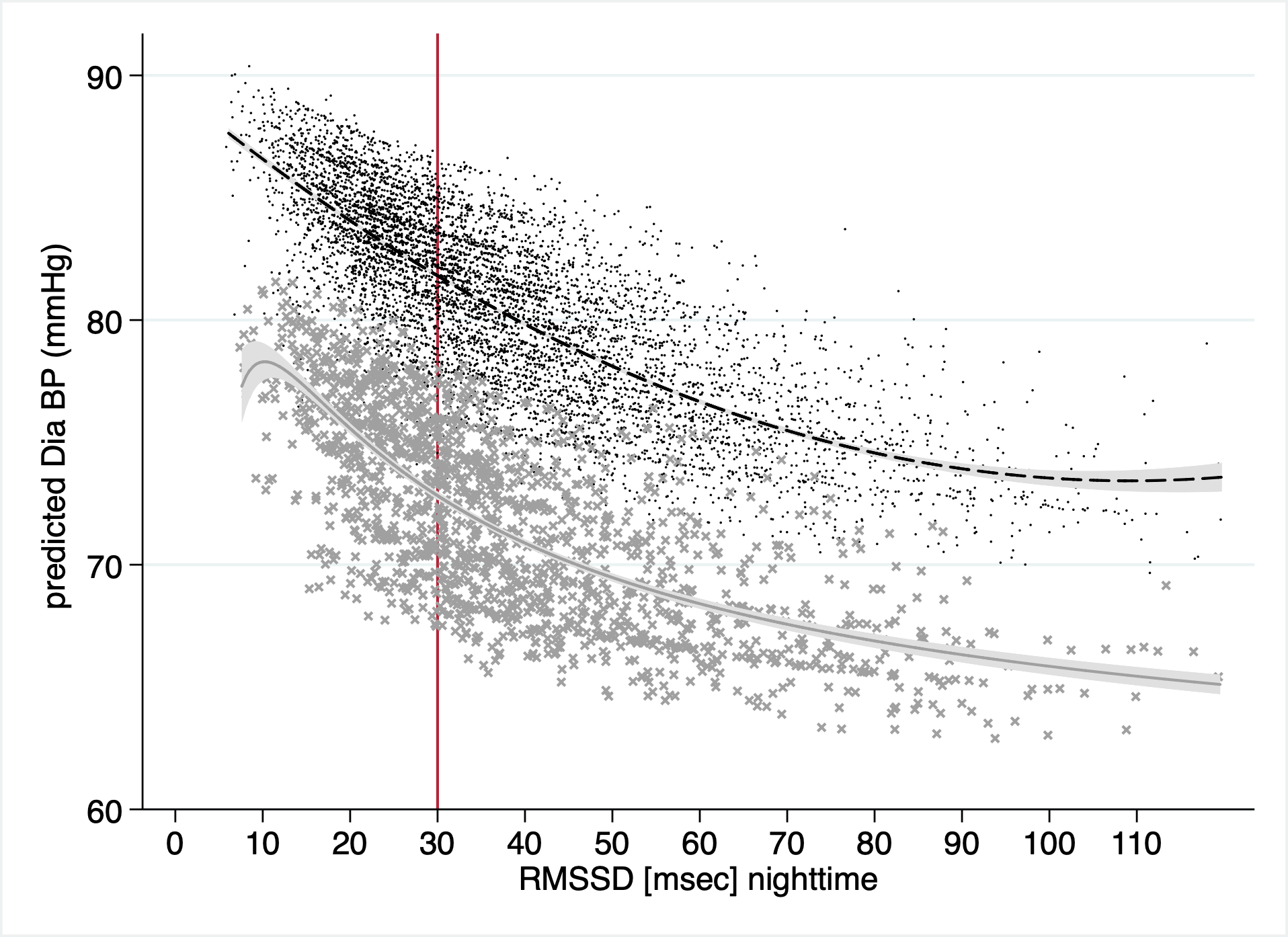

Supplement: Supplementary file 1 [file jcm-08-01940-s001.zip › supplements jcm_617360/rrdiam_predicted_night.png]

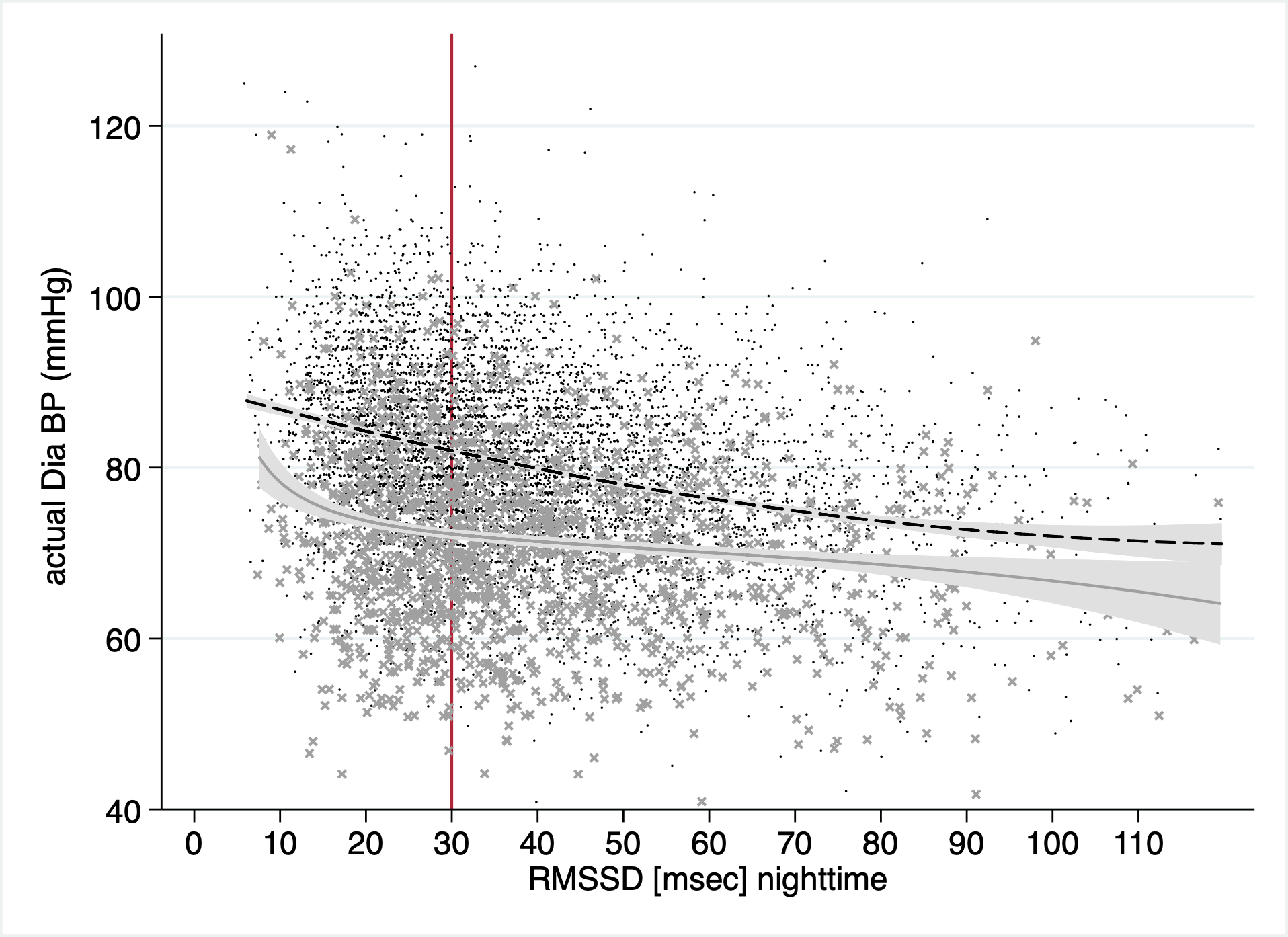

Supplement: Supplementary file 1 [file jcm-08-01940-s001.zip › supplements jcm_617360/rrdiam_actual_night.png]

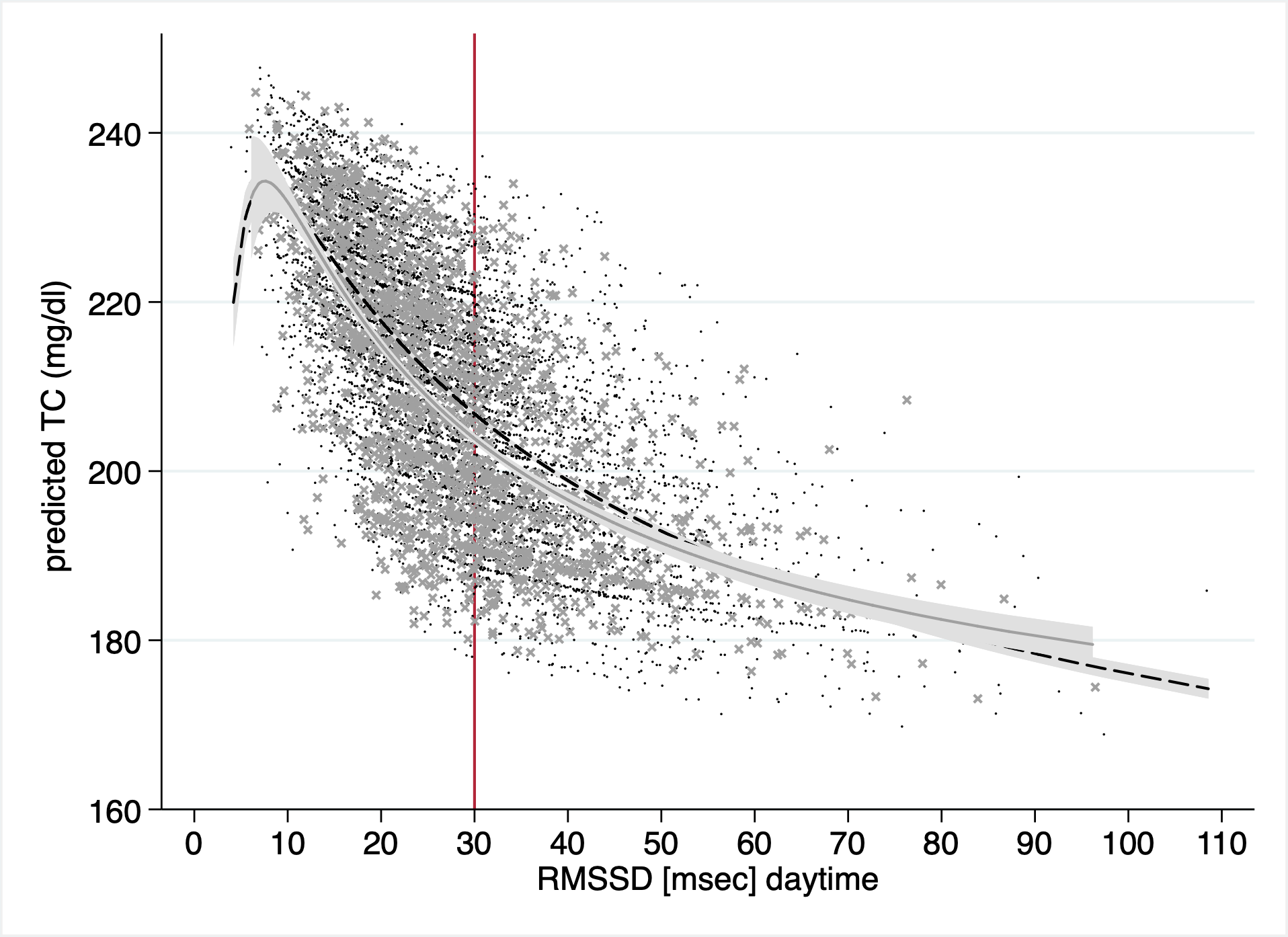

Supplement: Supplementary file 1 [file jcm-08-01940-s001.zip › supplements jcm_617360/chol_predicted_day.png]

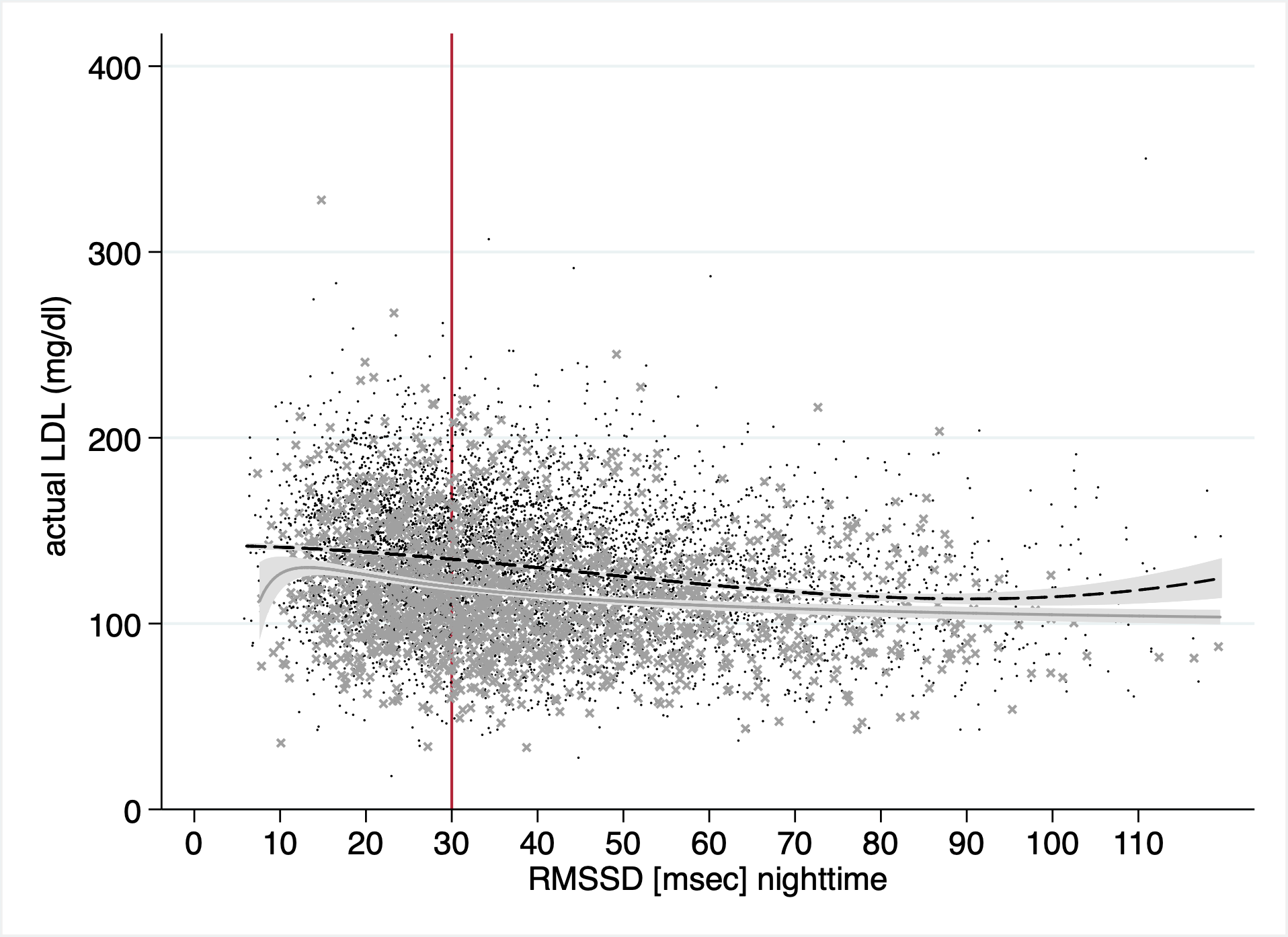

Supplement: Supplementary file 1 [file jcm-08-01940-s001.zip › supplements jcm_617360/ldllg_actual_night.png]

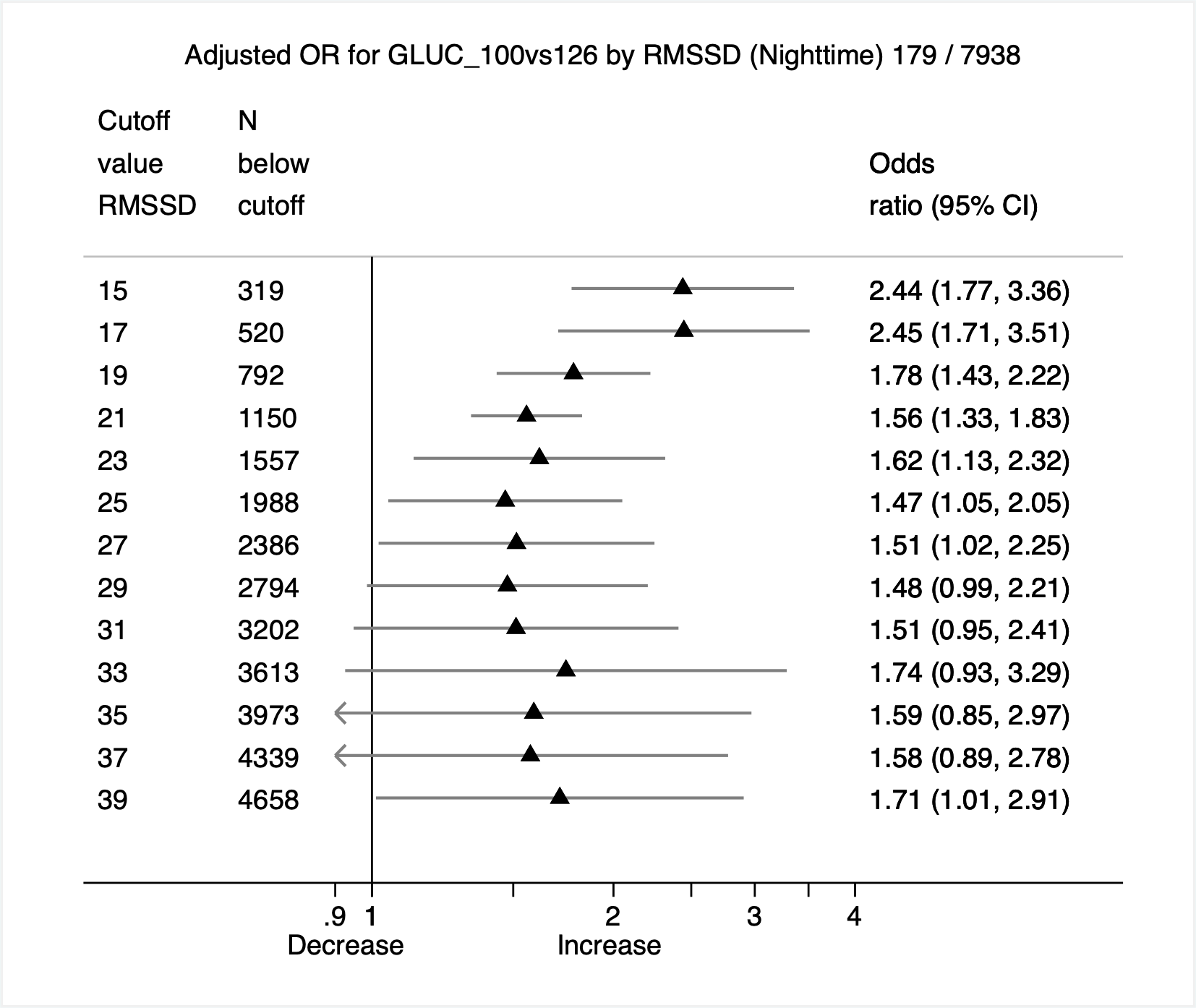

Supplement: Supplementary file 1 [file jcm-08-01940-s001.zip › supplements jcm_617360/HvsC_night_GLUC_100vs126.png]

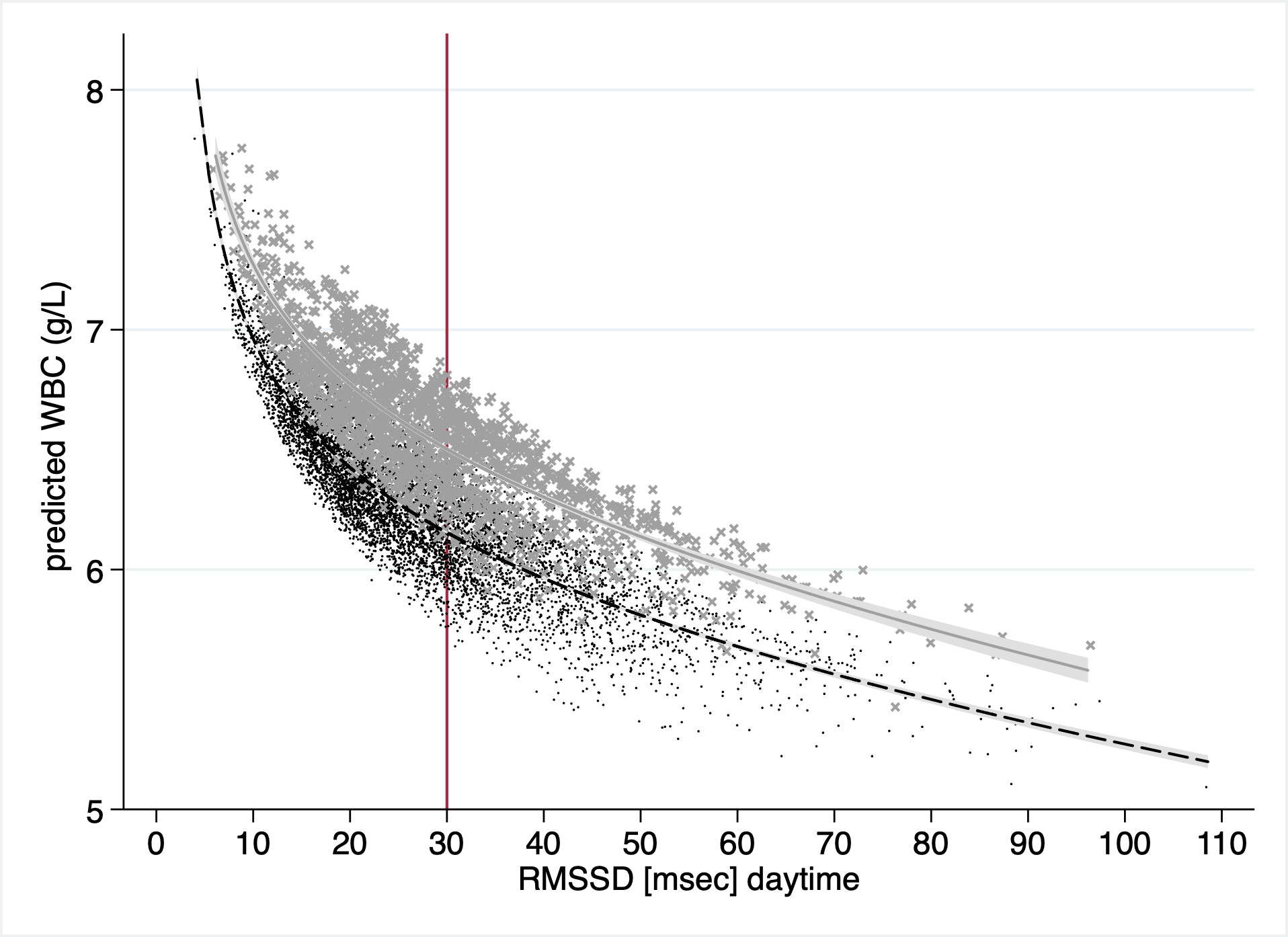

Supplement: Supplementary file 1 [file jcm-08-01940-s001.zip › supplements jcm_617360/leuk_predicted_day.png]

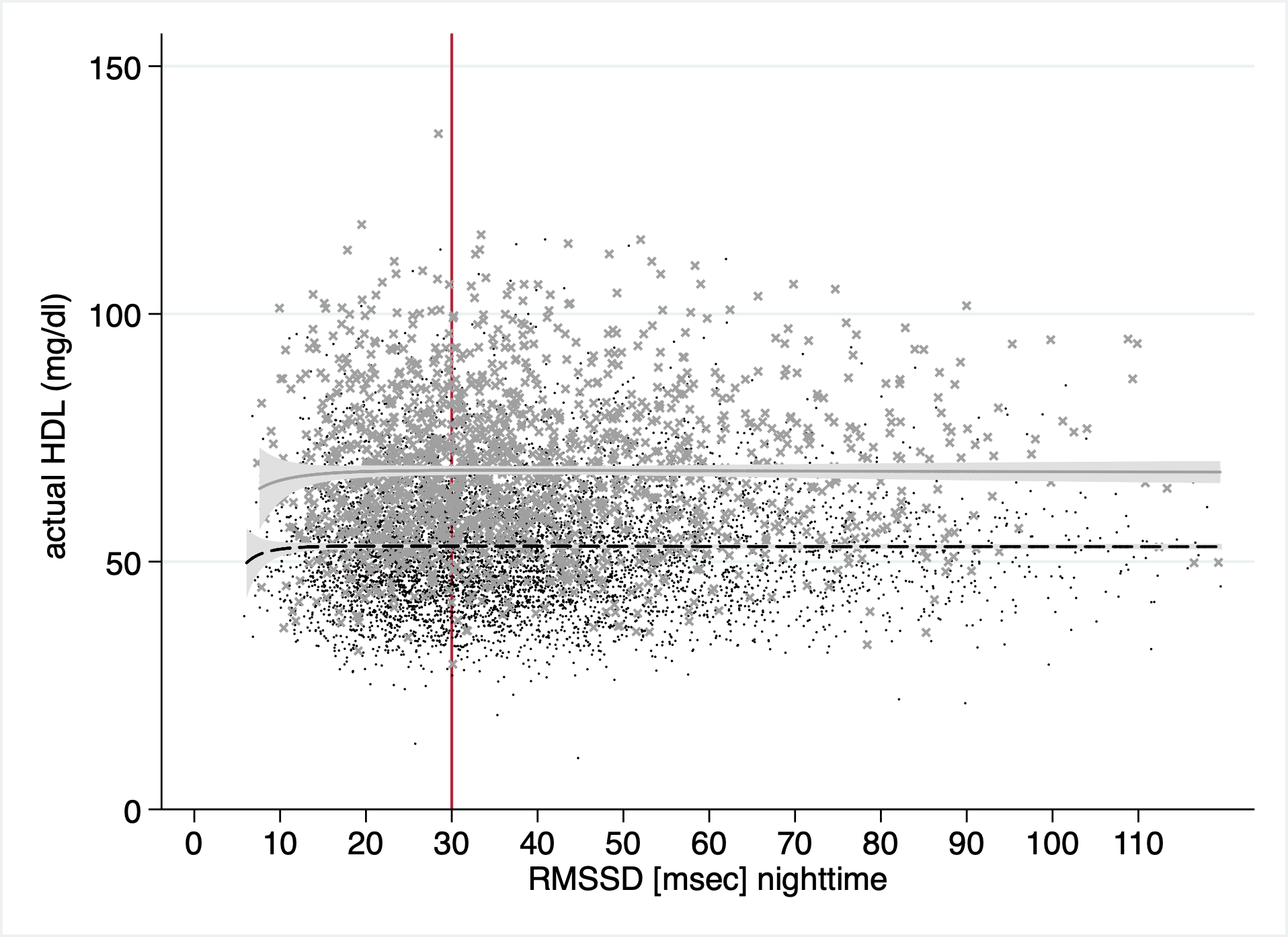

Supplement: Supplementary file 1 [file jcm-08-01940-s001.zip › supplements jcm_617360/hdl_actual_night.png]

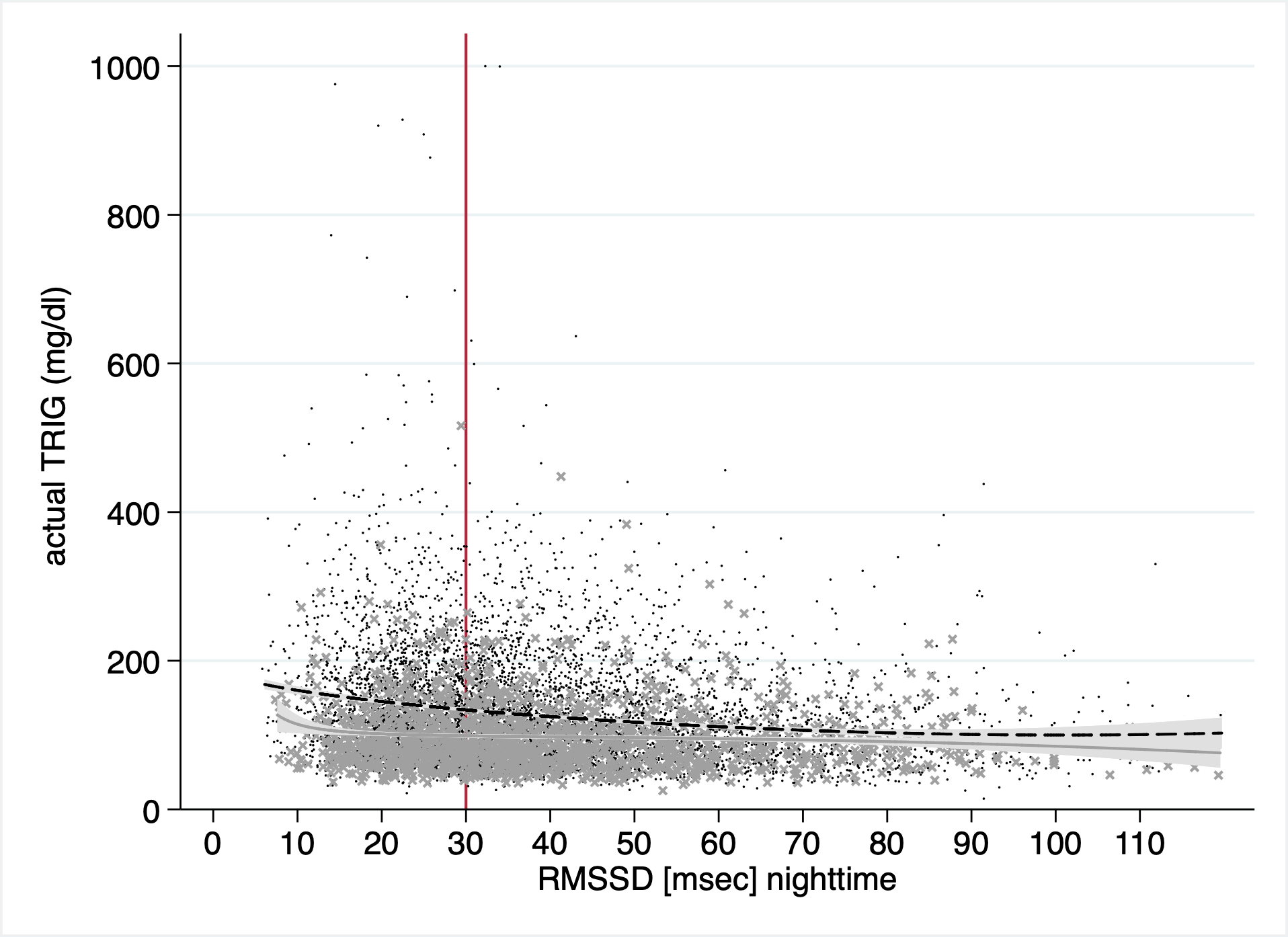

Supplement: Supplementary file 1 [file jcm-08-01940-s001.zip › supplements jcm_617360/trig_actual_night.png]

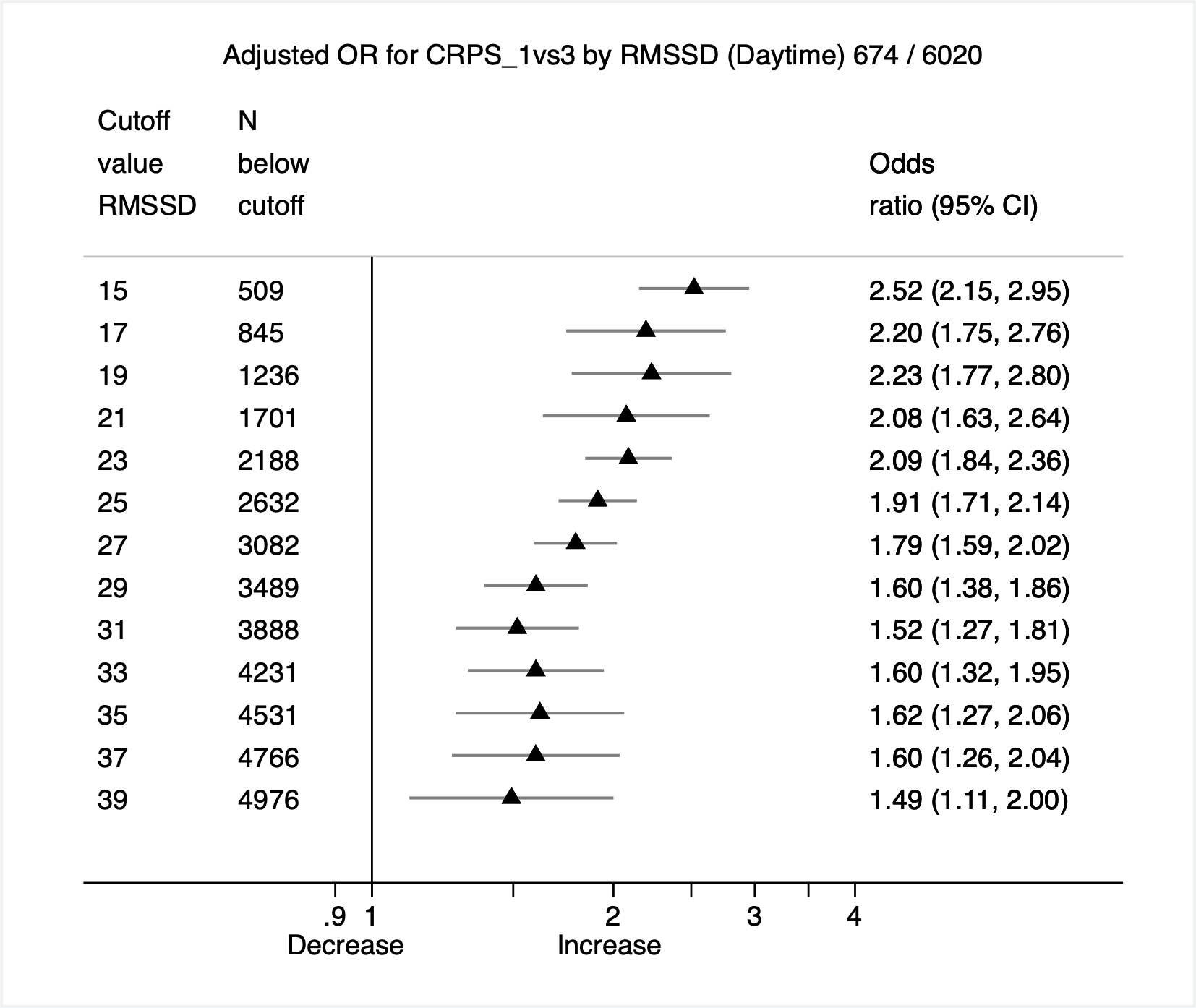

Supplement: Supplementary file 1 [file jcm-08-01940-s001.zip › supplements jcm_617360/HvsC_day_CRPS_1vs3.png]

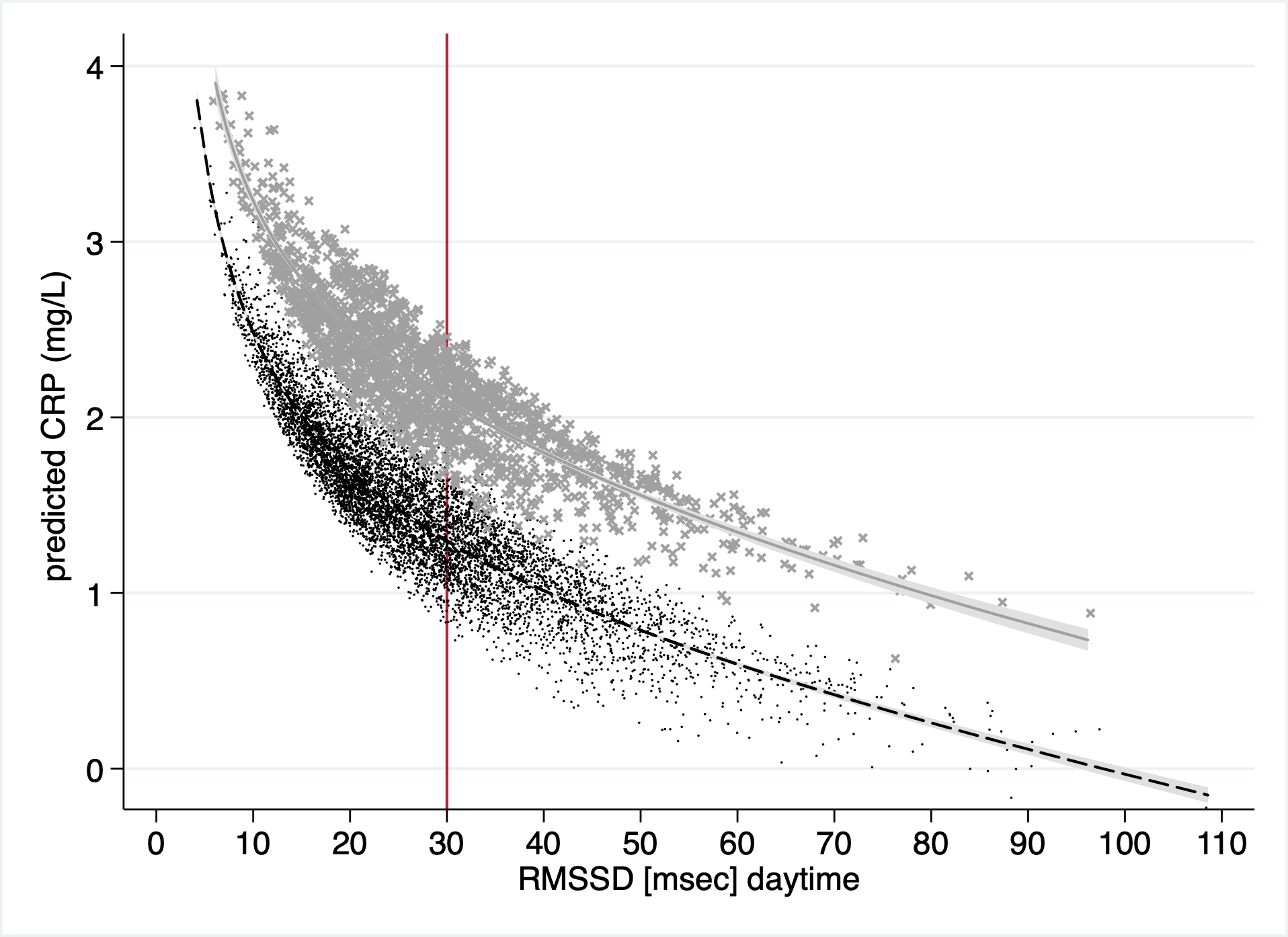

Supplement: Supplementary file 1 [file jcm-08-01940-s001.zip › supplements jcm_617360/crps_predicted_day.png]

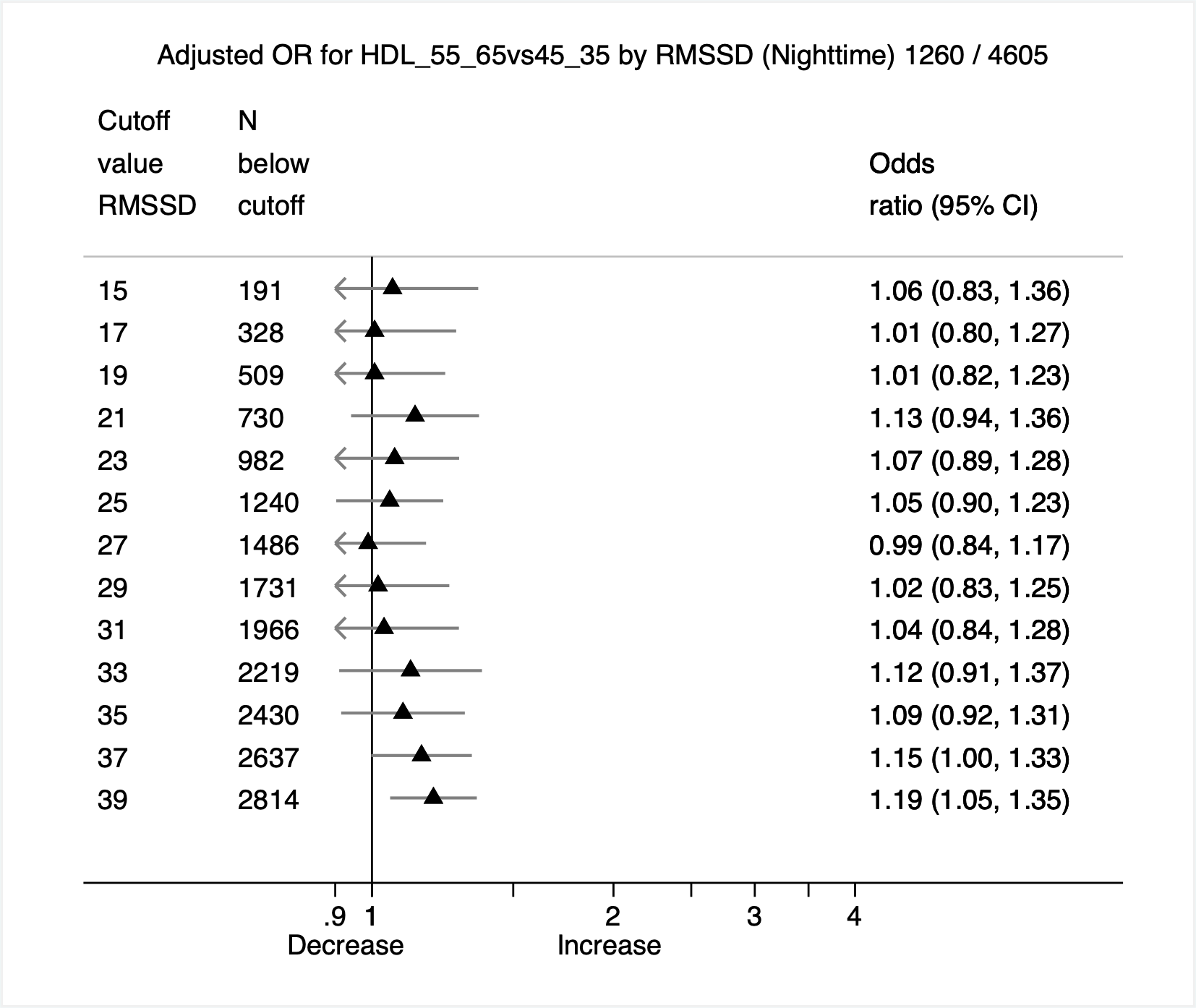

Supplement: Supplementary file 1 [file jcm-08-01940-s001.zip › supplements jcm_617360/HvsC_night_HDL_55_65vs45_35.png]

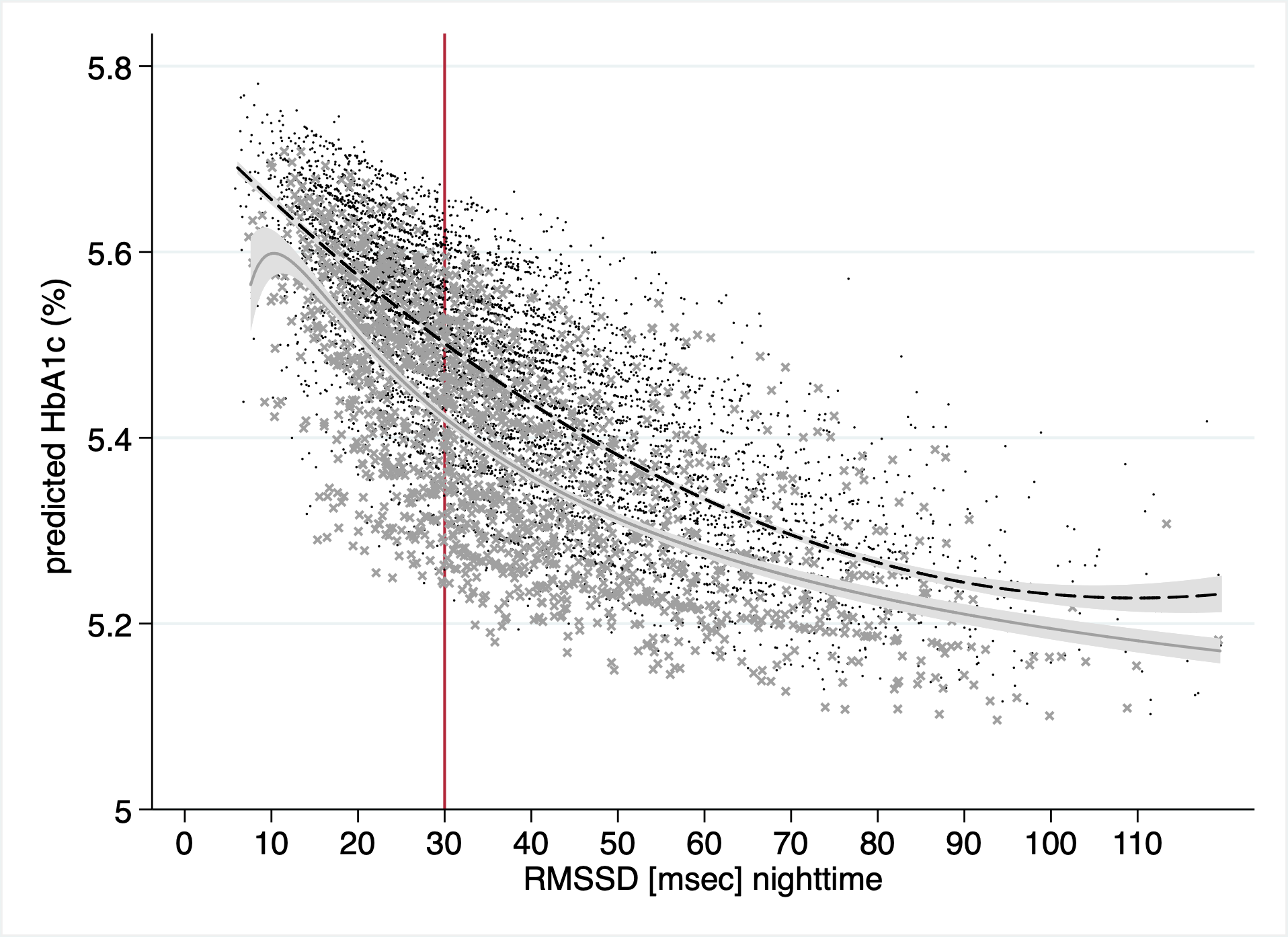

Supplement: Supplementary file 1 [file jcm-08-01940-s001.zip › supplements jcm_617360/hba1c_predicted_night.png]

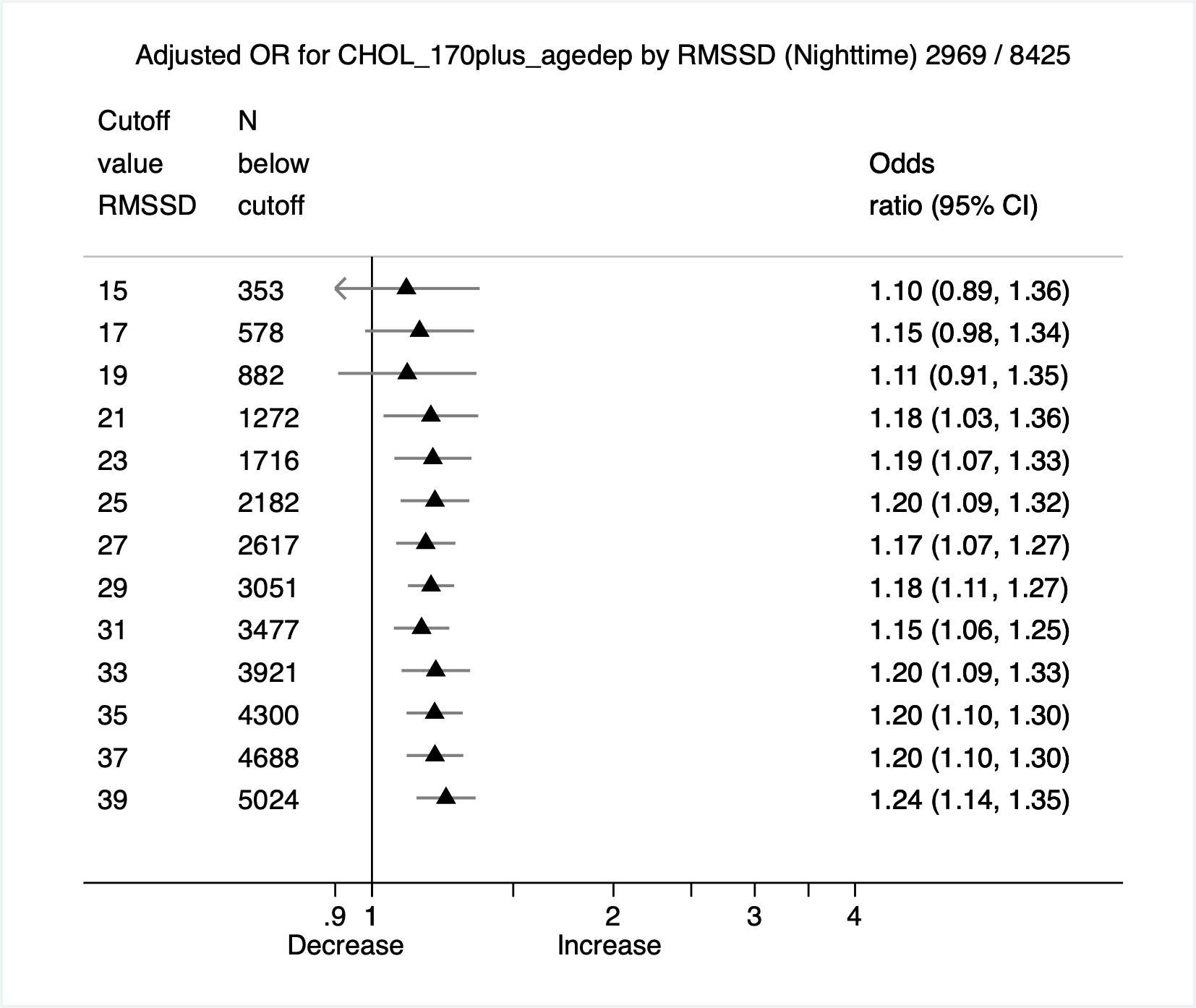

Supplement: Supplementary file 1 [file jcm-08-01940-s001.zip › supplements jcm_617360/HvsC_night_CHOL_170plus_agedep.png]

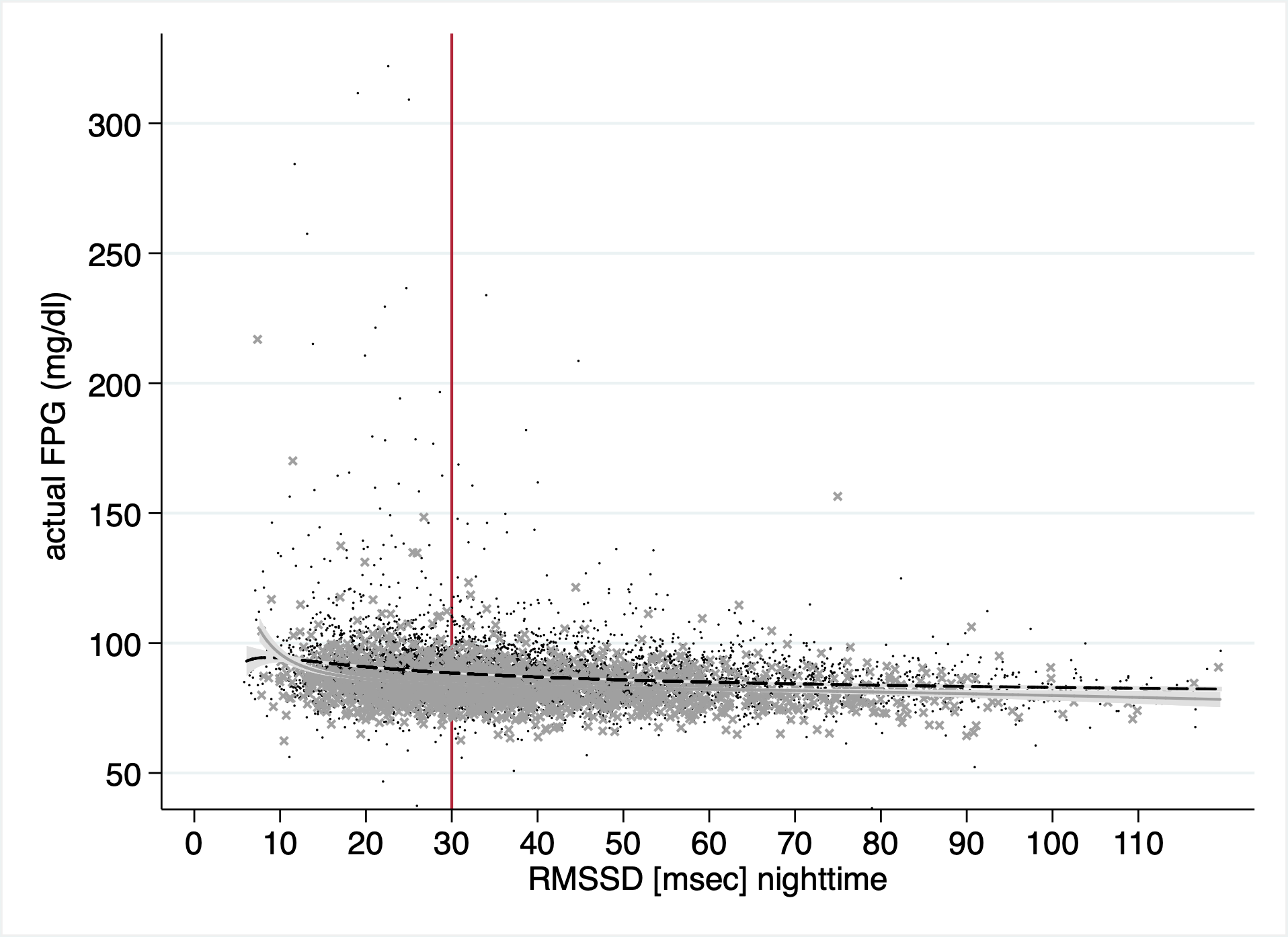

Supplement: Supplementary file 1 [file jcm-08-01940-s001.zip › supplements jcm_617360/glucn_actual_night.png]

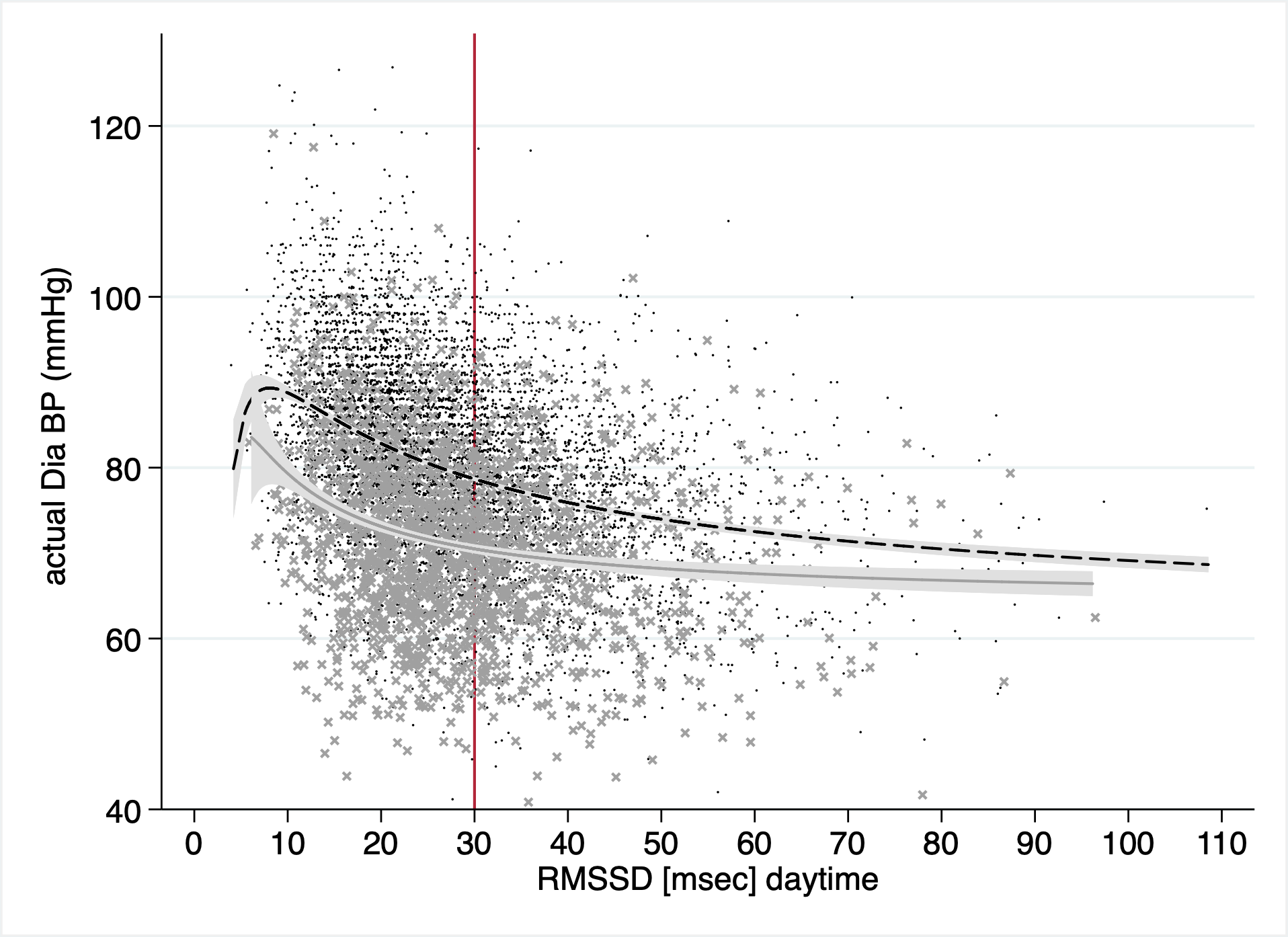

Supplement: Supplementary file 1 [file jcm-08-01940-s001.zip › supplements jcm_617360/rrdiam_actual_day.png]

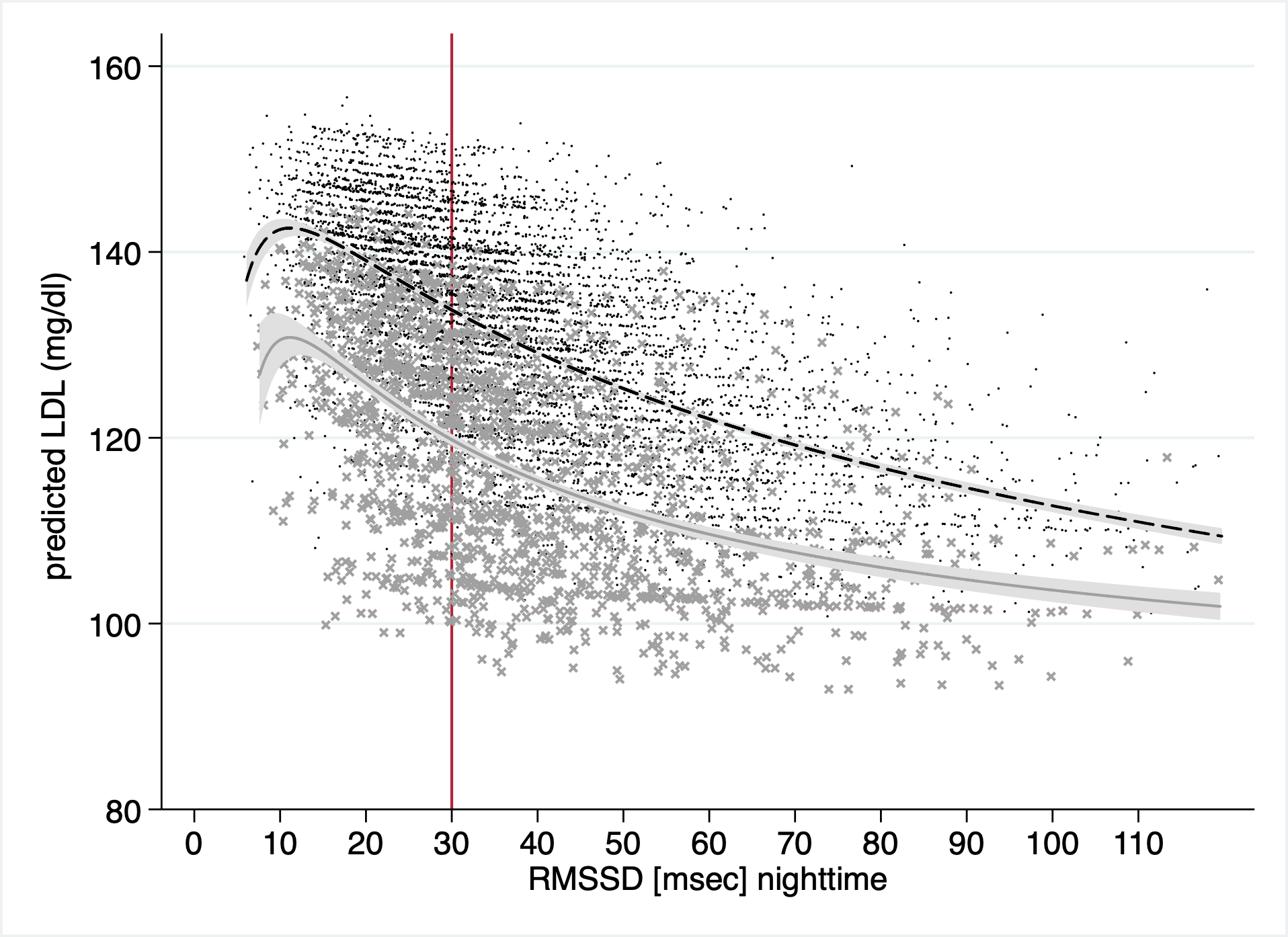

Supplement: Supplementary file 1 [file jcm-08-01940-s001.zip › supplements jcm_617360/ldllg_predicted_night.png]

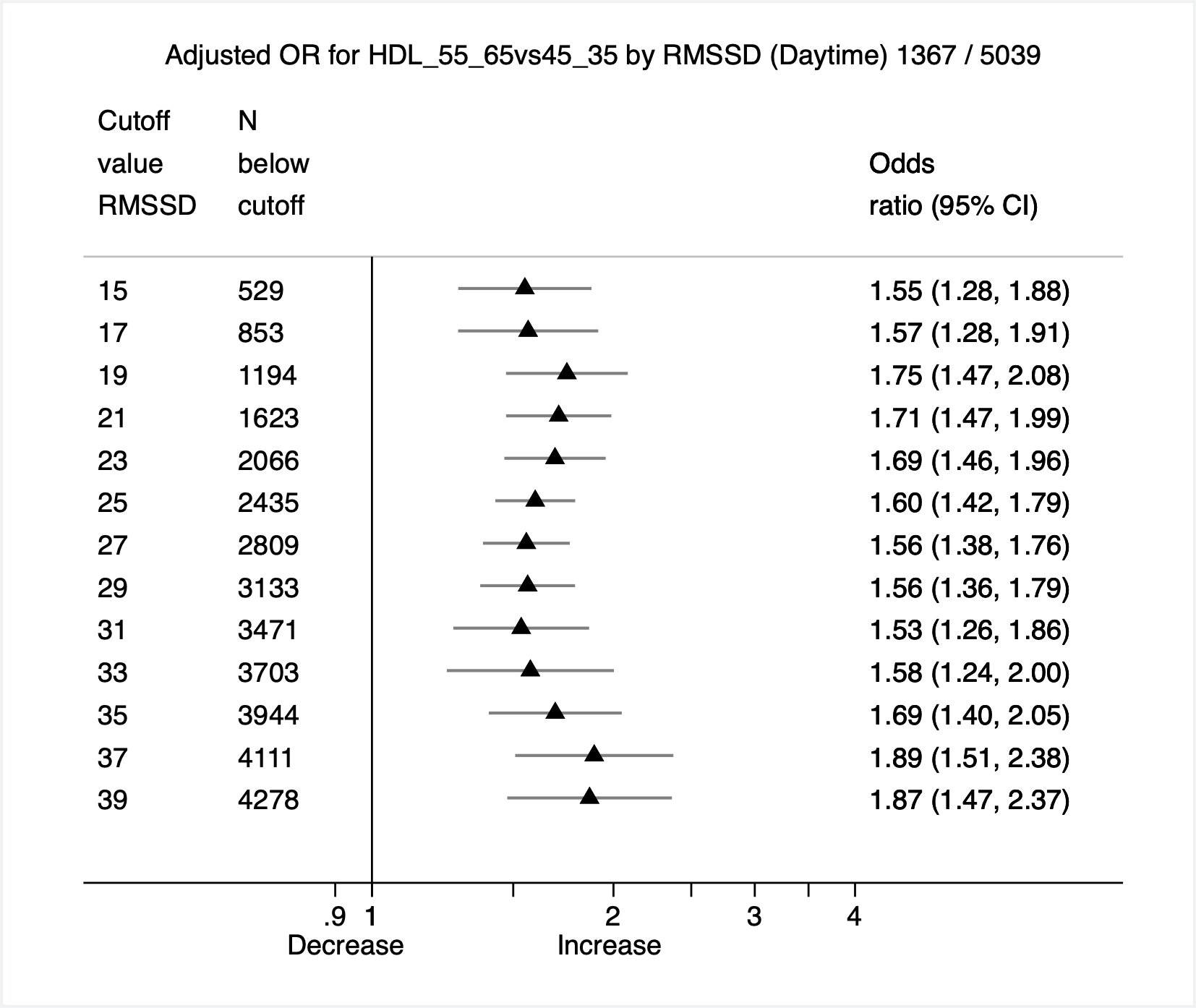

Supplement: Supplementary file 1 [file jcm-08-01940-s001.zip › supplements jcm_617360/HvsC_day_HDL_55_65vs45_35.png]

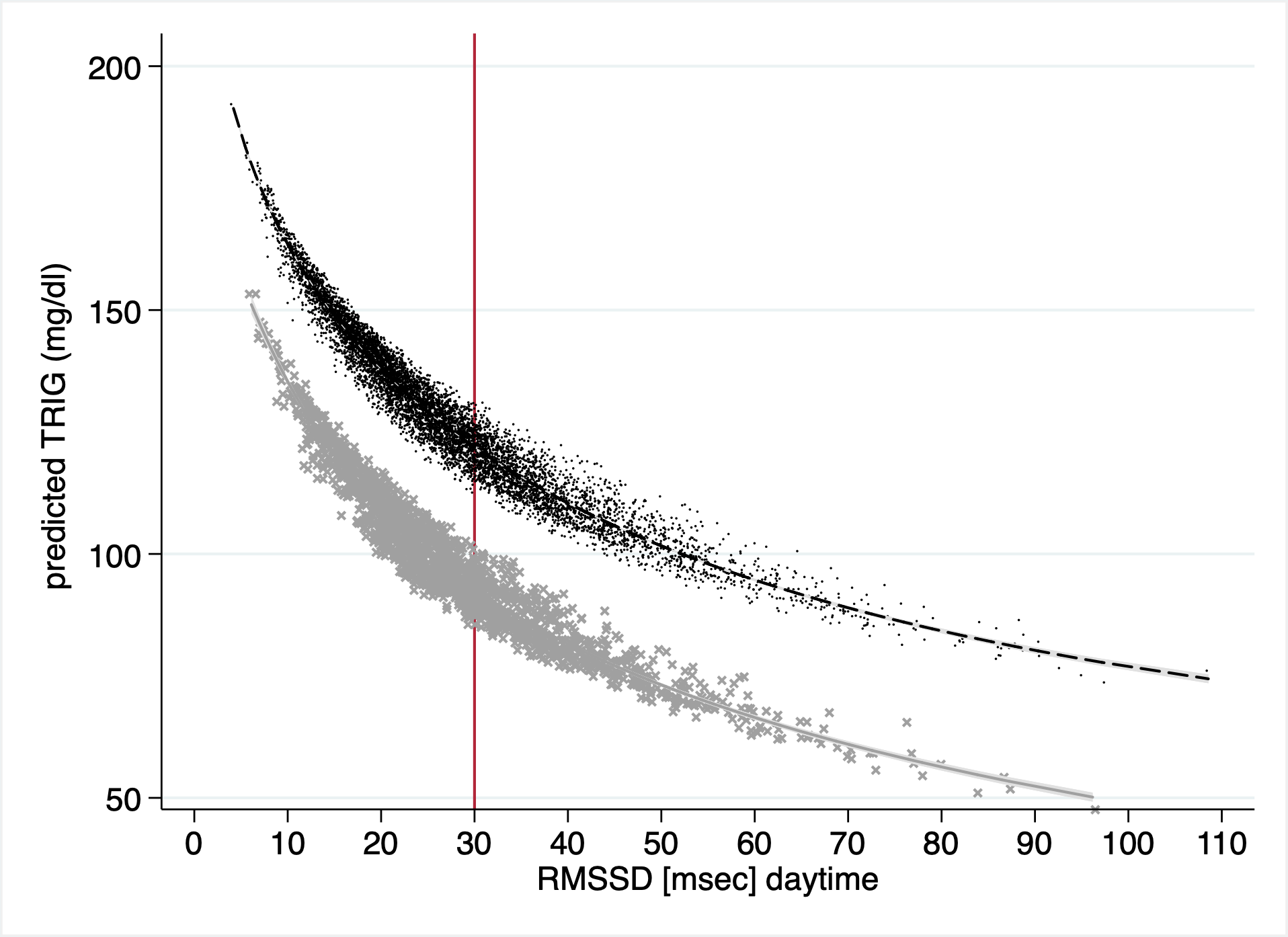

Supplement: Supplementary file 1 [file jcm-08-01940-s001.zip › supplements jcm_617360/trig_predicted_day.png]

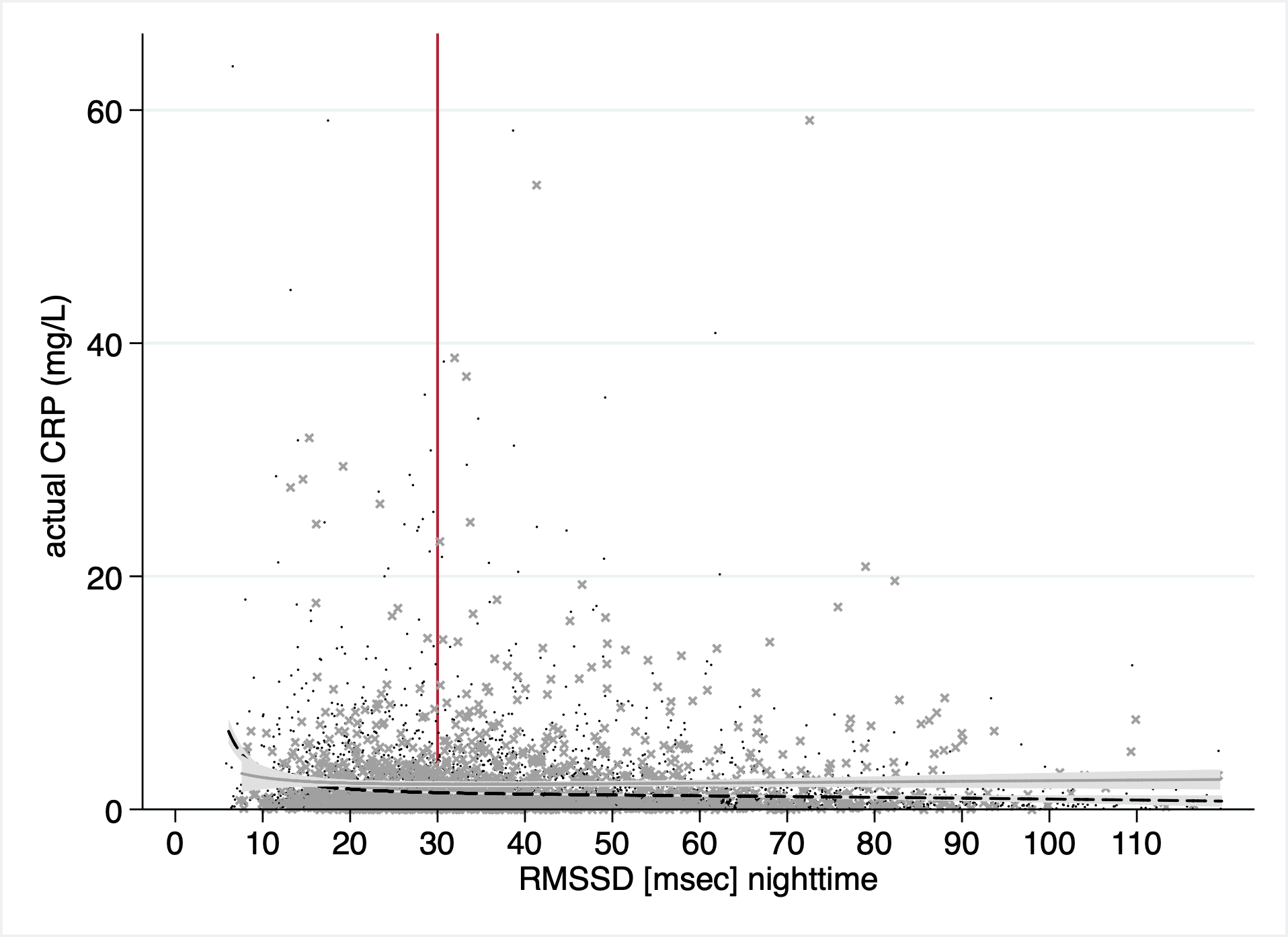

Supplement: Supplementary file 1 [file jcm-08-01940-s001.zip › supplements jcm_617360/crps_actual_night.png]

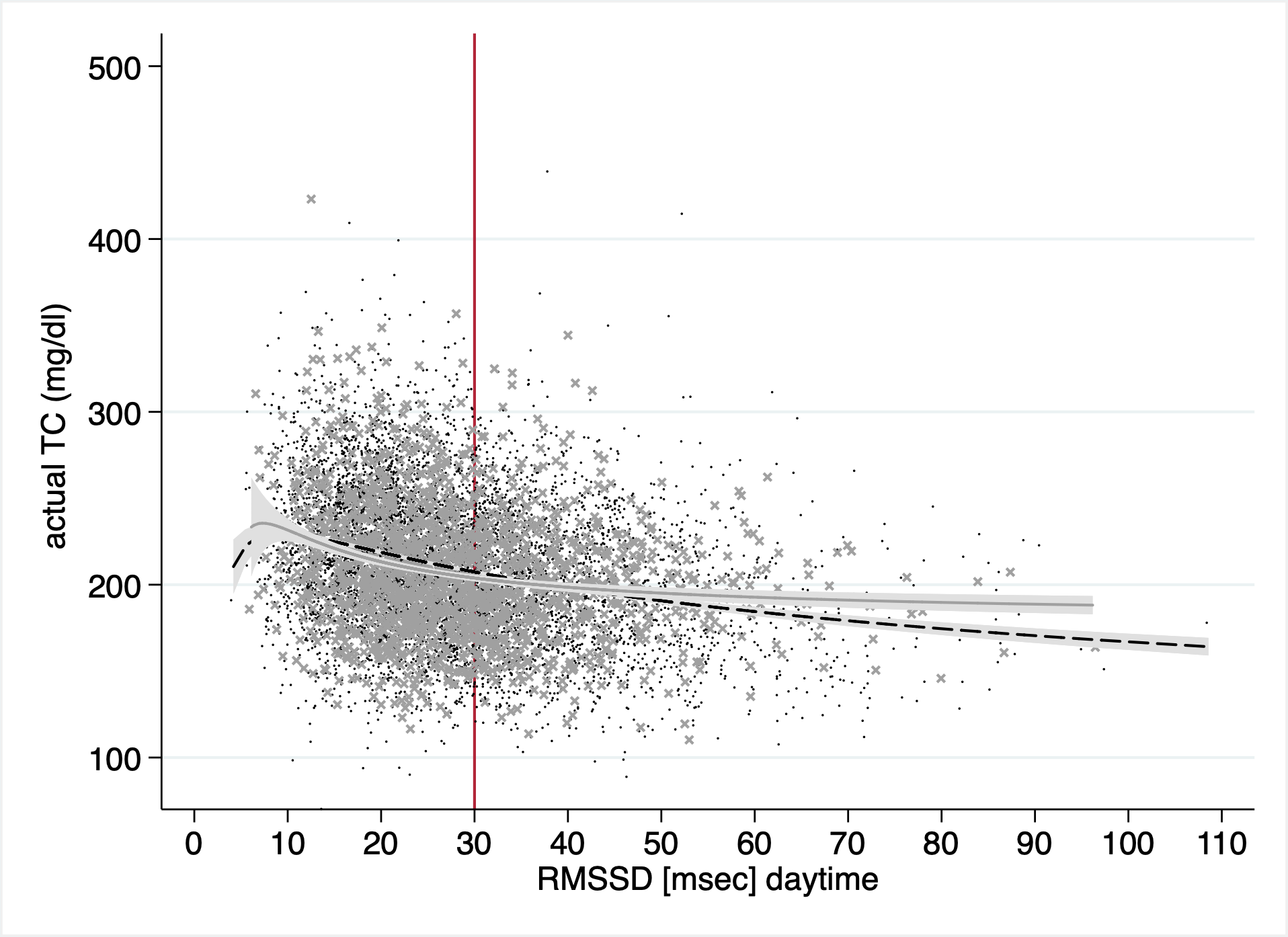

Supplement: Supplementary file 1 [file jcm-08-01940-s001.zip › supplements jcm_617360/chol_actual_day.png]

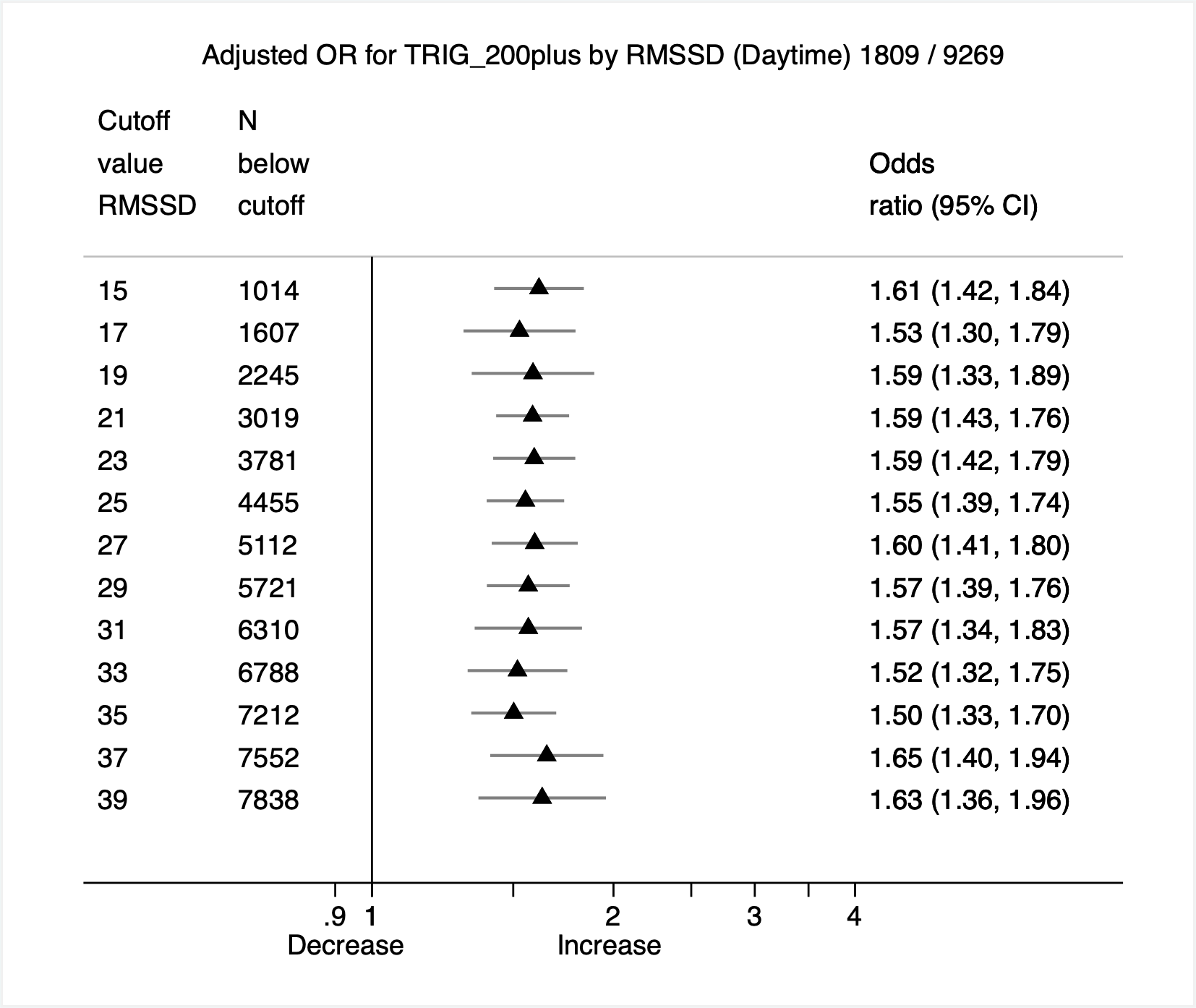

Supplement: Supplementary file 1 [file jcm-08-01940-s001.zip › supplements jcm_617360/HvsC_day_TRIG_200plus.png]

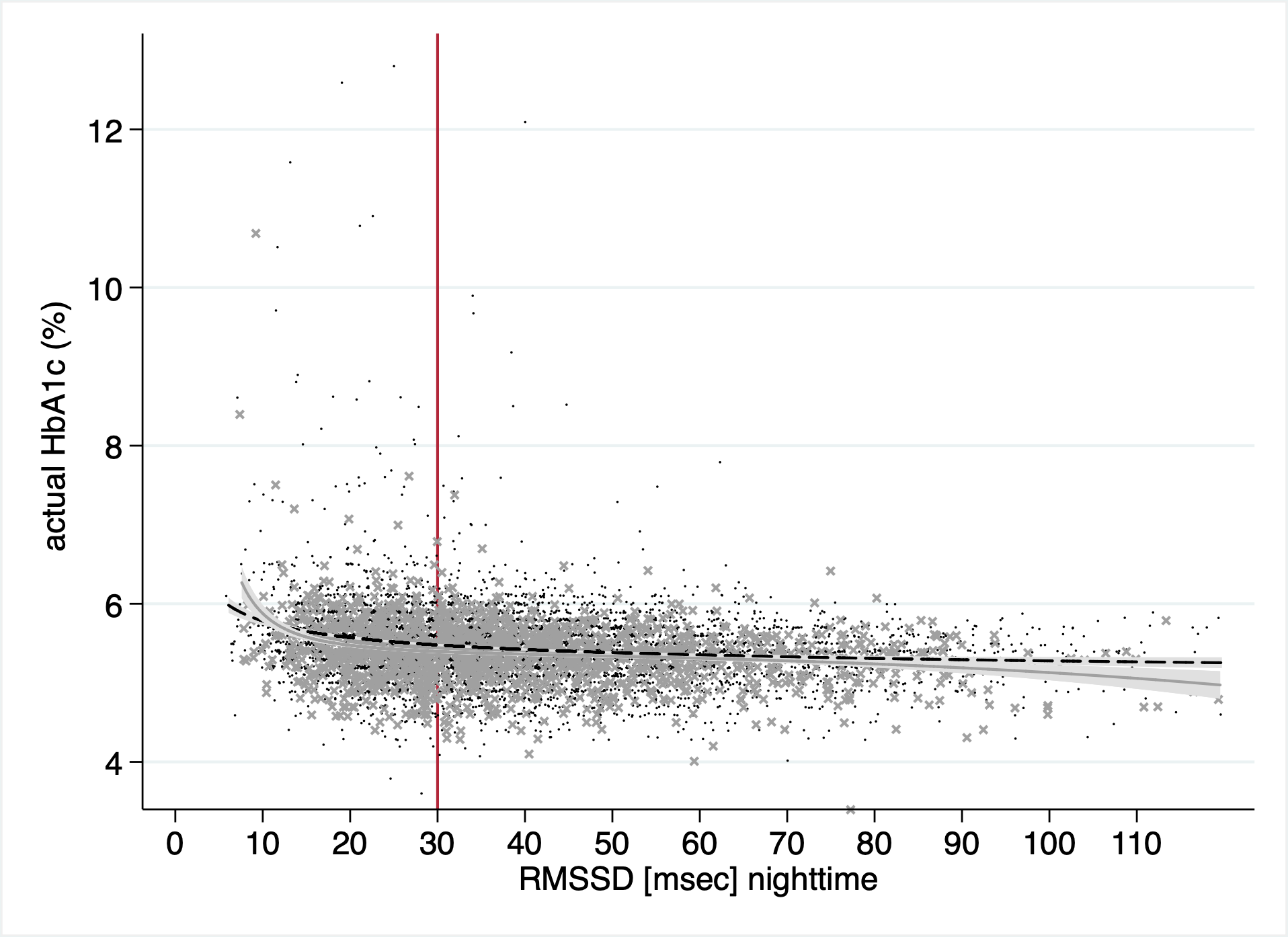

Supplement: Supplementary file 1 [file jcm-08-01940-s001.zip › supplements jcm_617360/hba1c_actual_night.png]

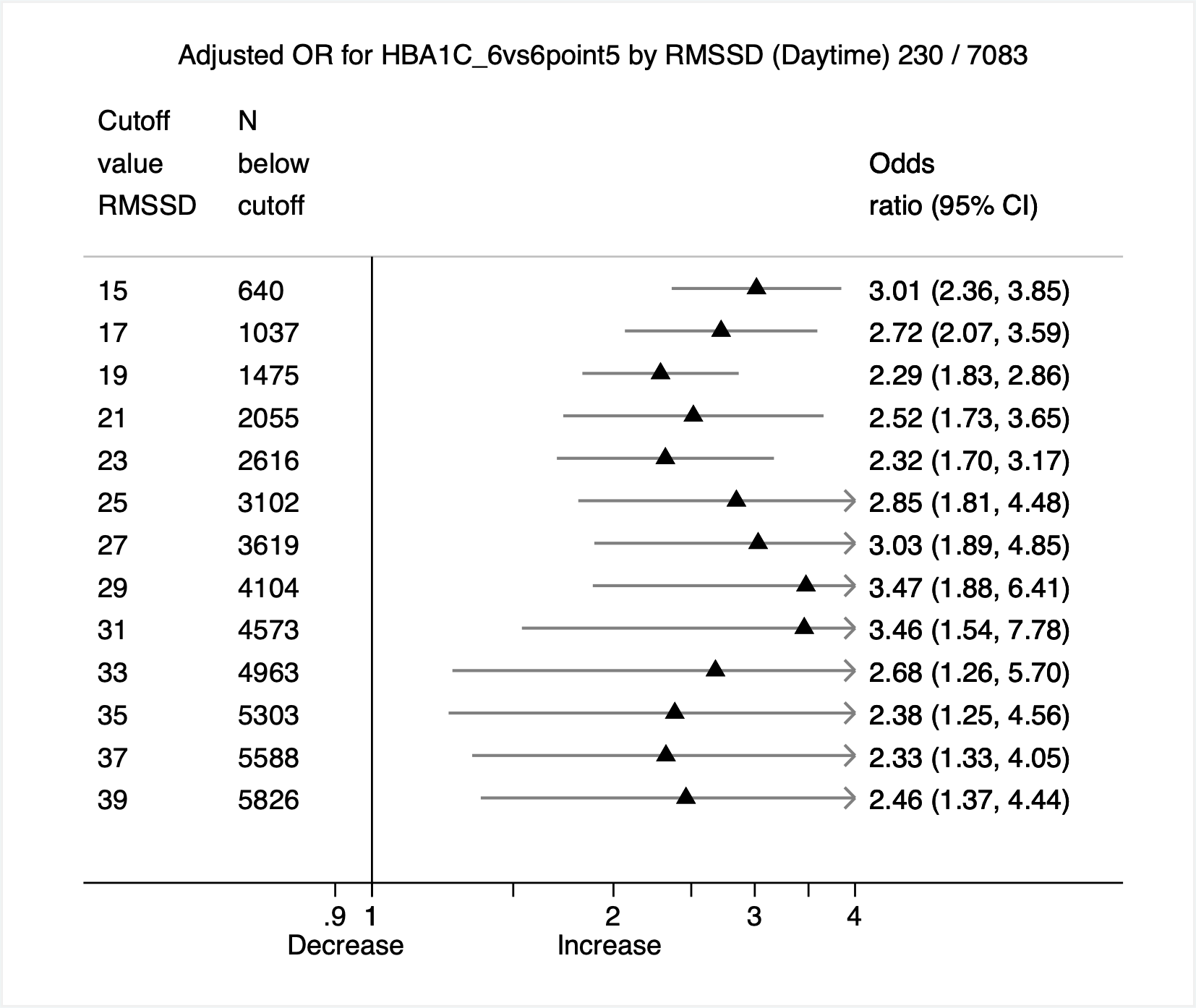

Supplement: Supplementary file 1 [file jcm-08-01940-s001.zip › supplements jcm_617360/HvsC_day_HBA1C_6vs6point5.png]

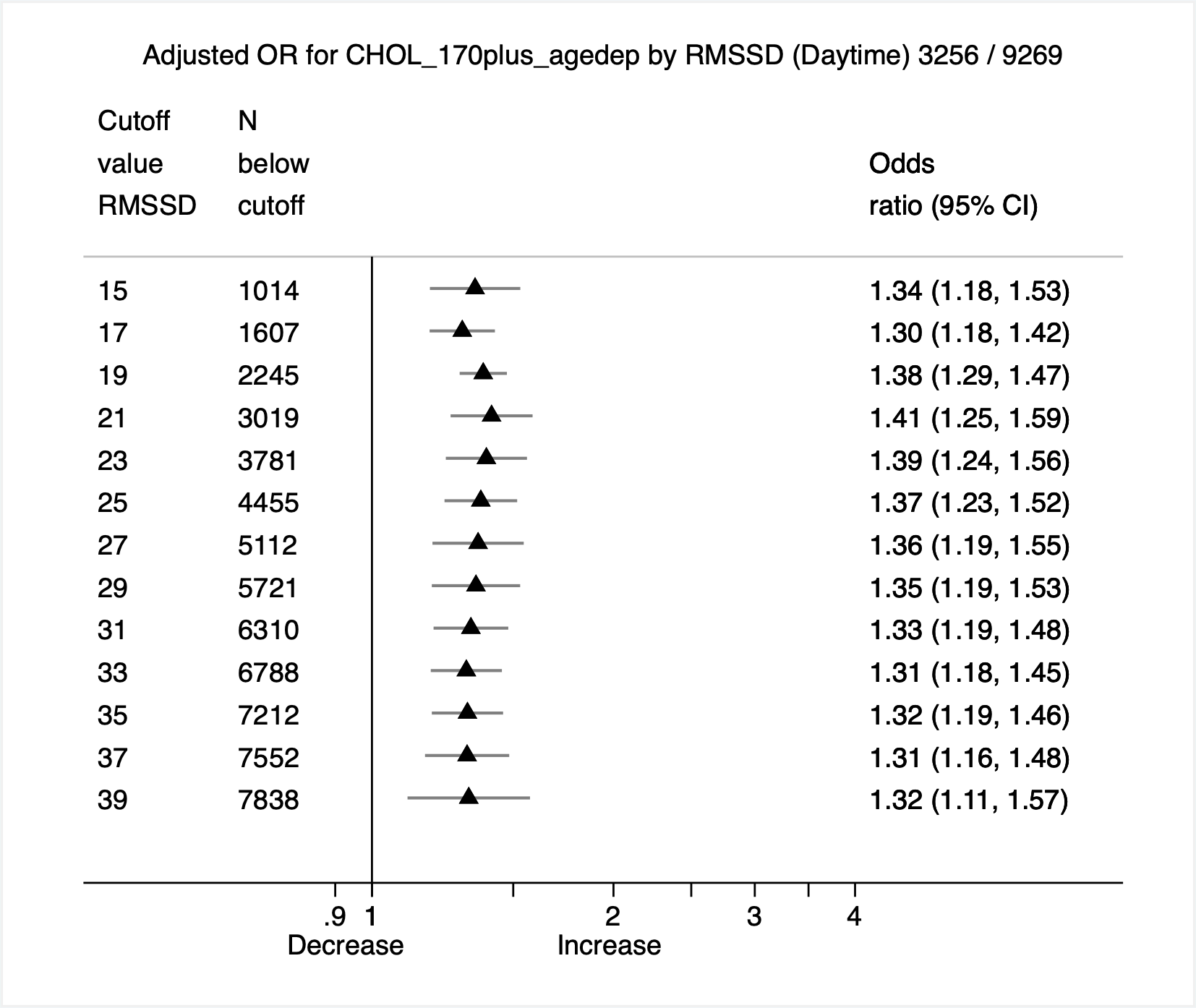

Supplement: Supplementary file 1 [file jcm-08-01940-s001.zip › supplements jcm_617360/HvsC_day_CHOL_170plus_agedep.png]

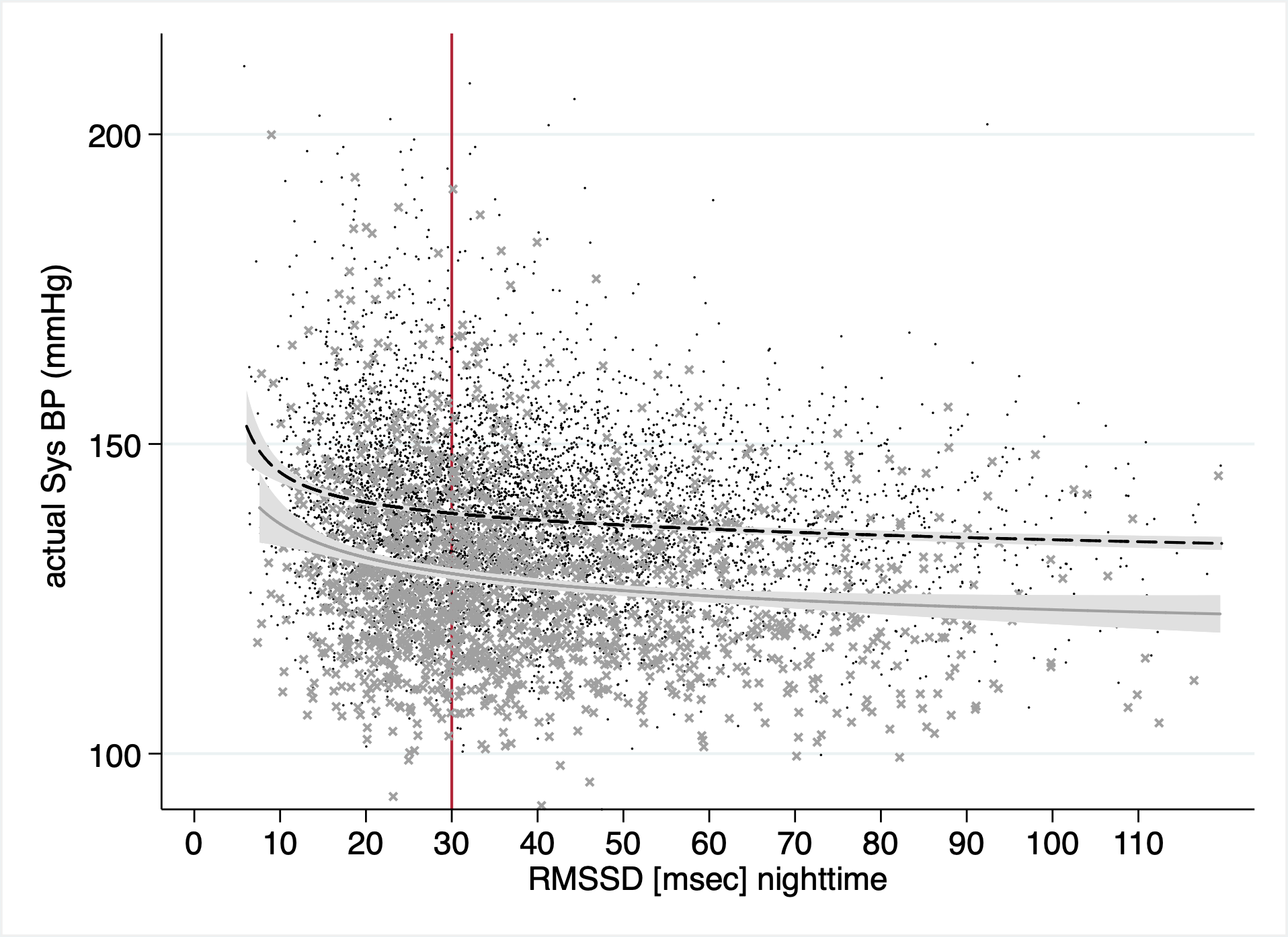

Supplement: Supplementary file 1 [file jcm-08-01940-s001.zip › supplements jcm_617360/rrsysm_actual_night.png]

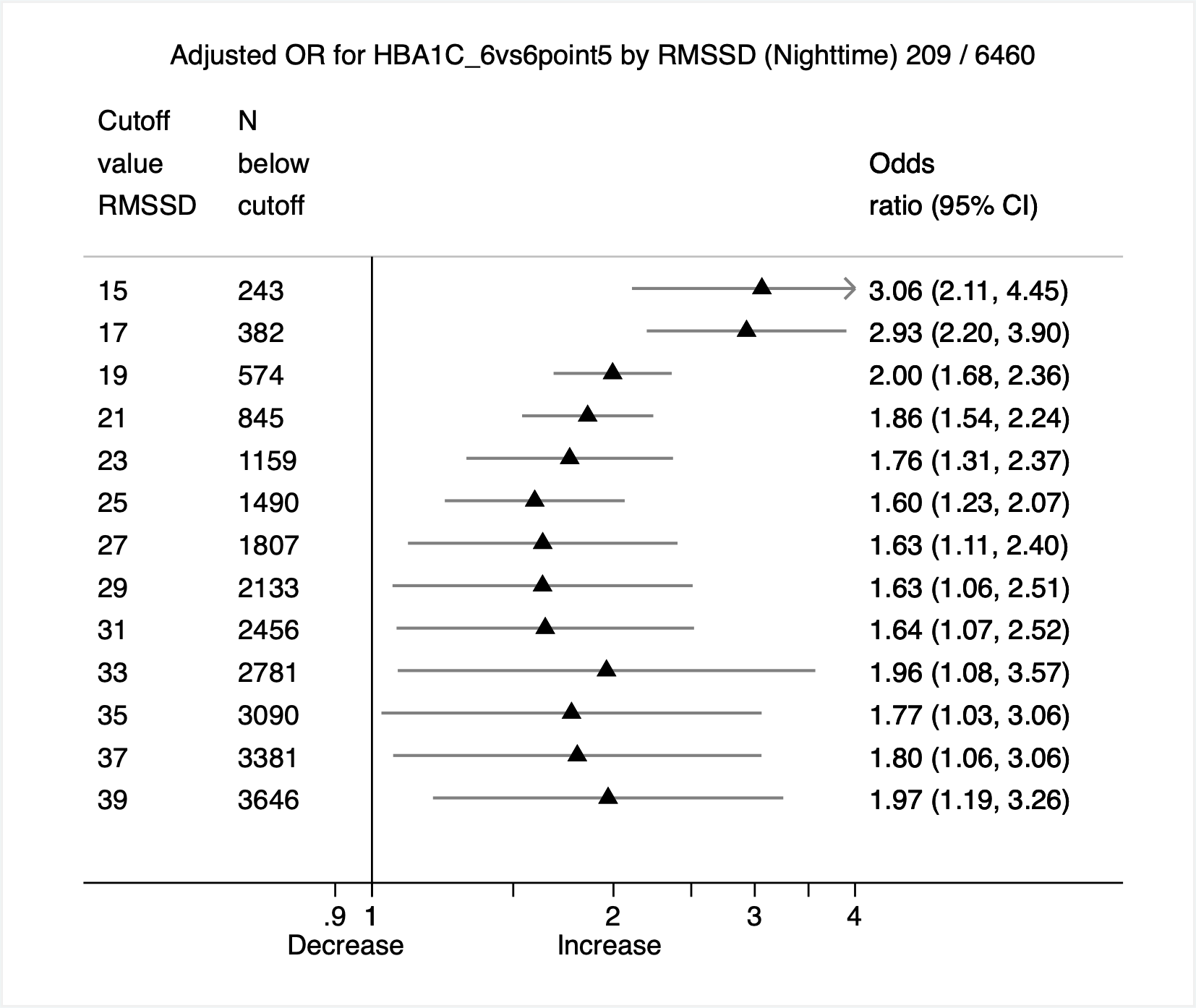

Supplement: Supplementary file 1 [file jcm-08-01940-s001.zip › supplements jcm_617360/HvsC_night_HBA1C_6vs6point5.png]

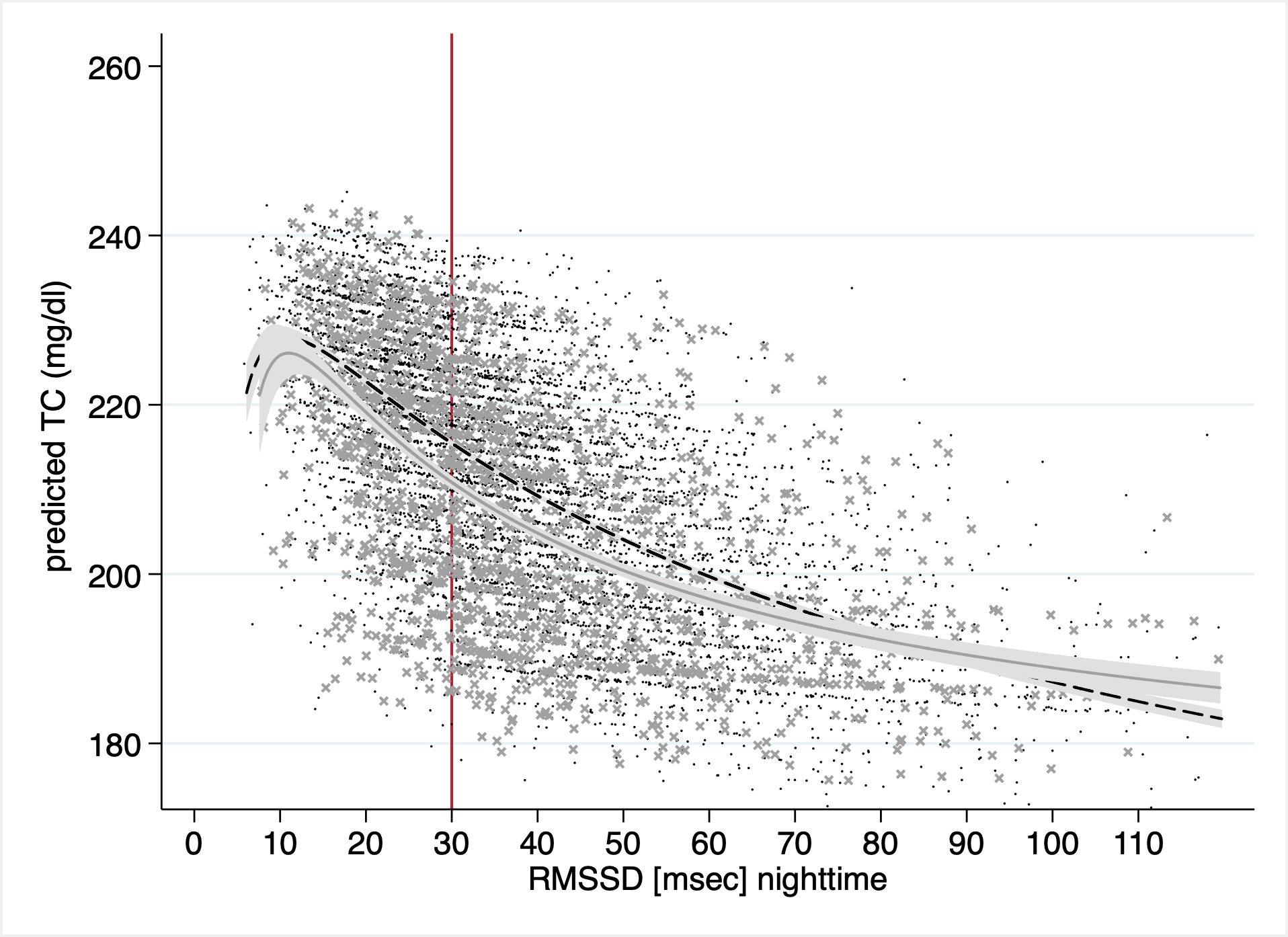

Supplement: Supplementary file 1 [file jcm-08-01940-s001.zip › supplements jcm_617360/chol_predicted_night.png]

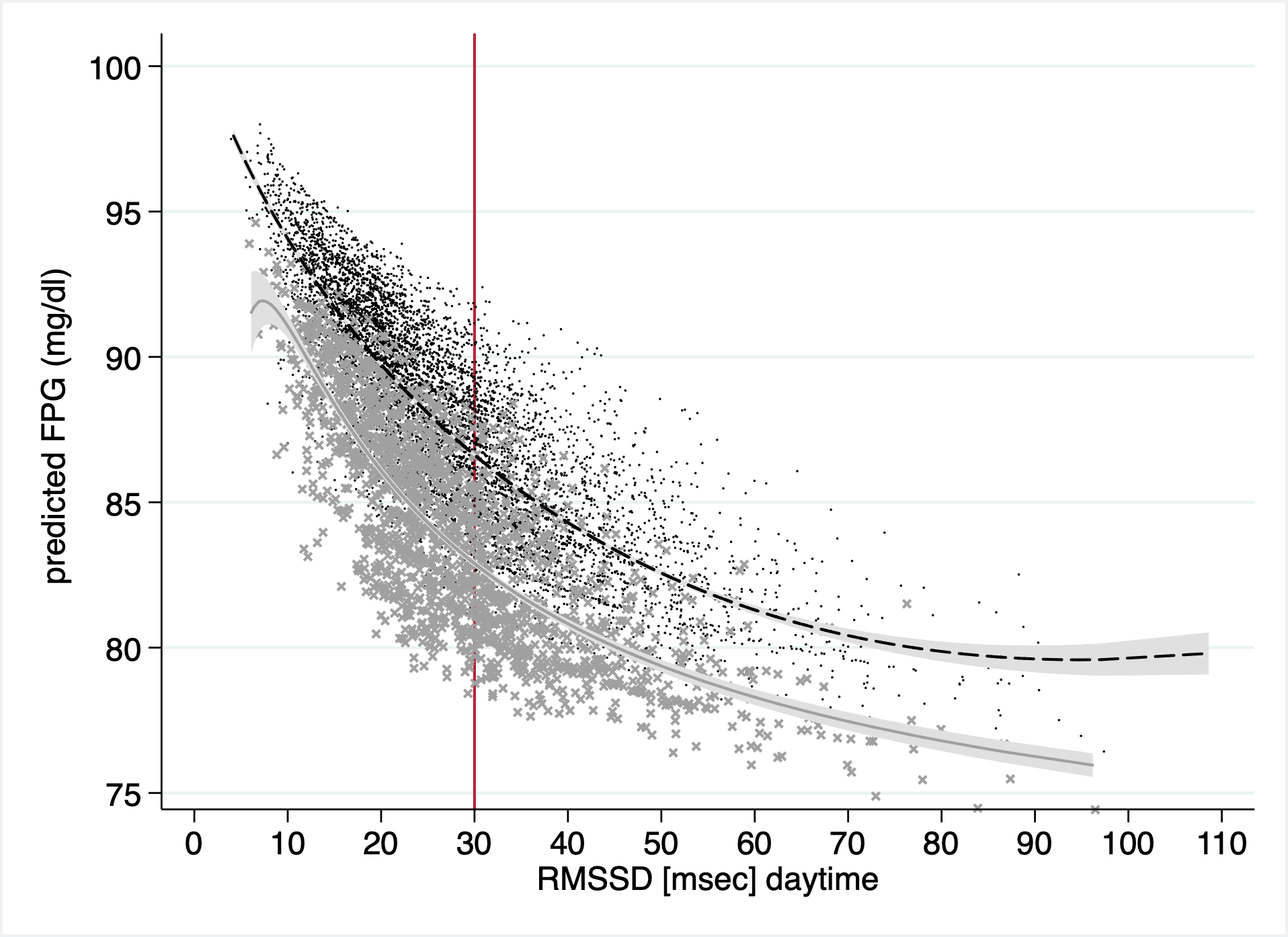

Supplement: Supplementary file 1 [file jcm-08-01940-s001.zip › supplements jcm_617360/glucn_predicted_day.png]

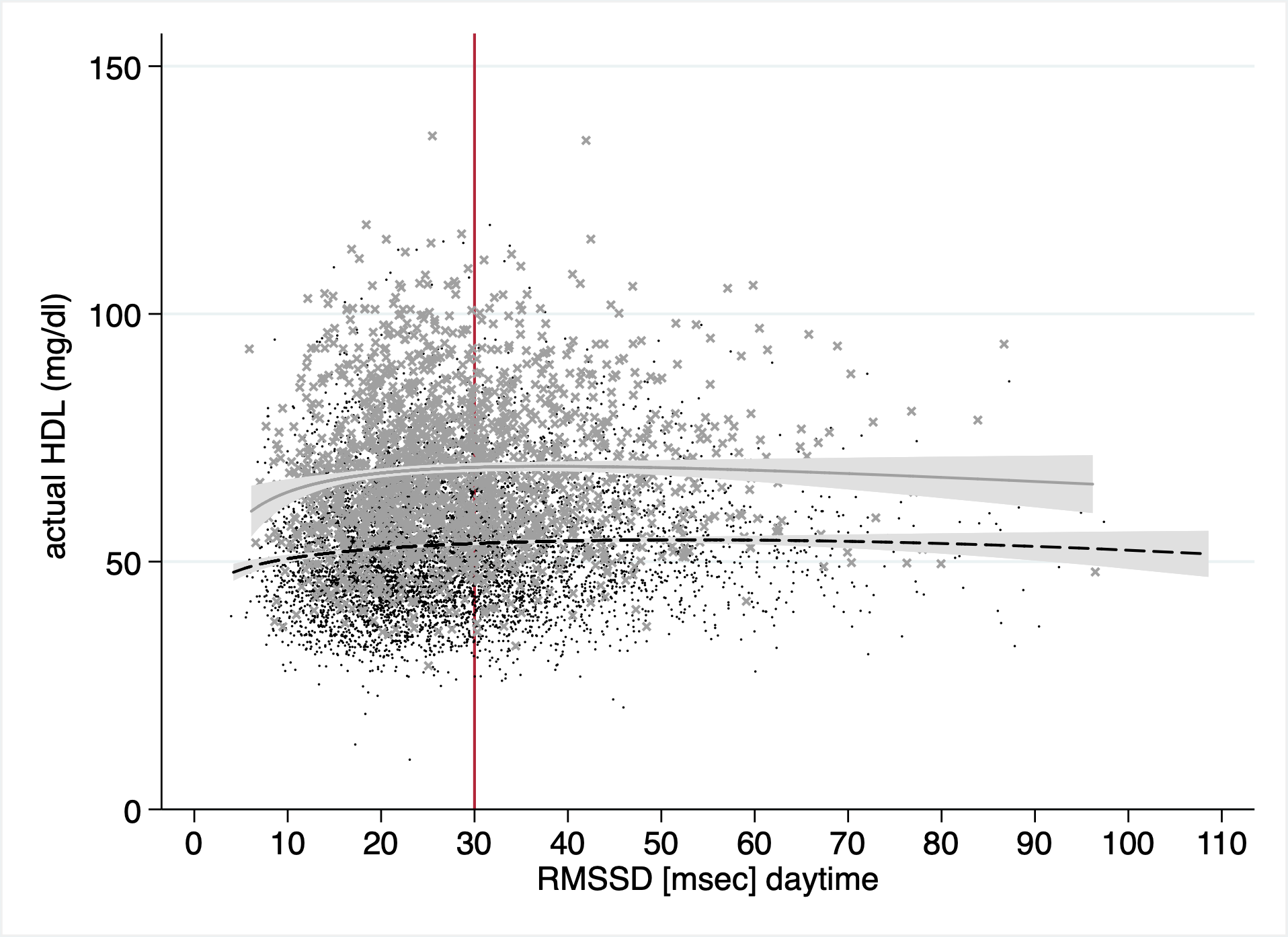

Supplement: Supplementary file 1 [file jcm-08-01940-s001.zip › supplements jcm_617360/hdl_actual_day.png]

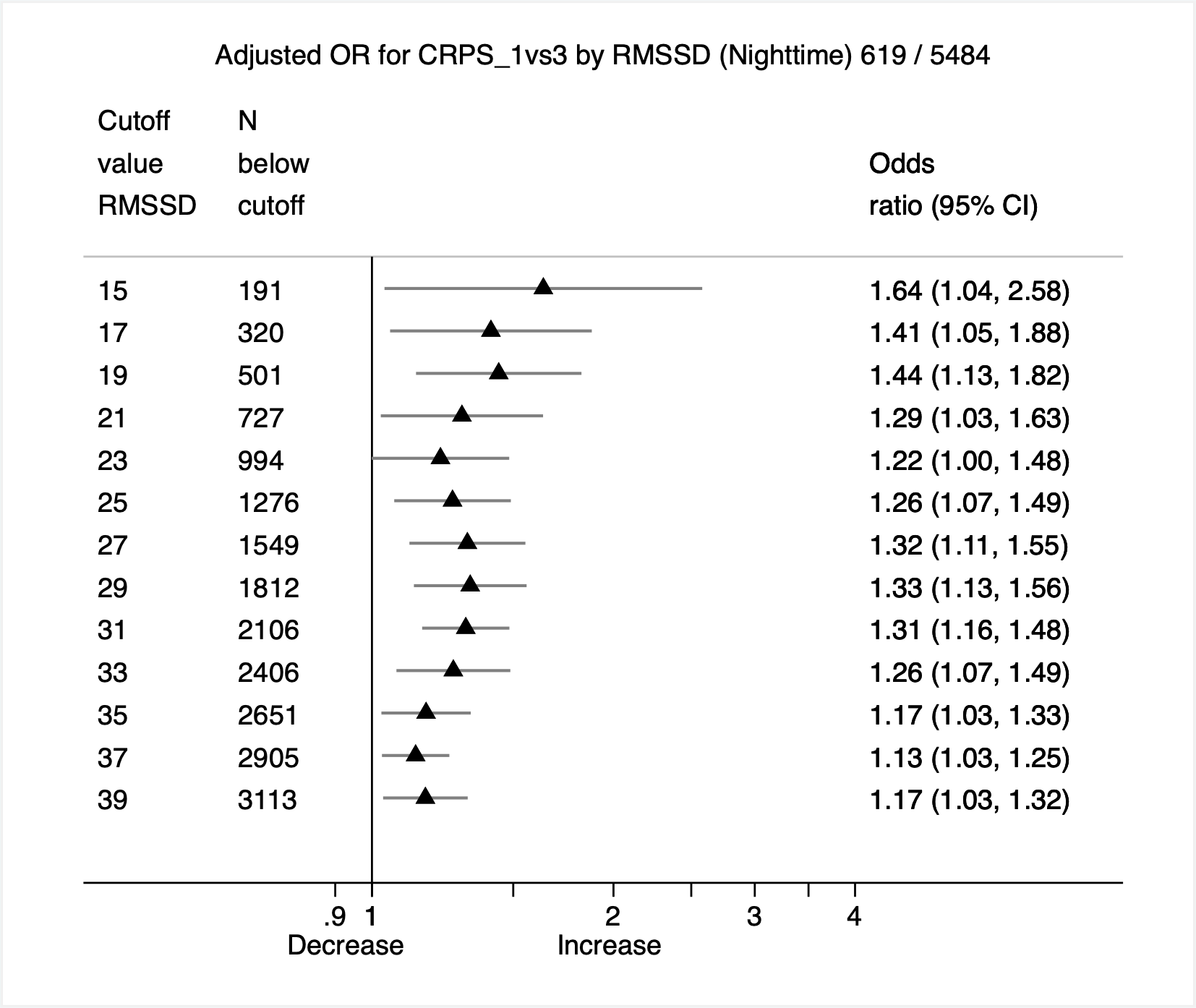

Supplement: Supplementary file 1 [file jcm-08-01940-s001.zip › supplements jcm_617360/HvsC_night_CRPS_1vs3.png]

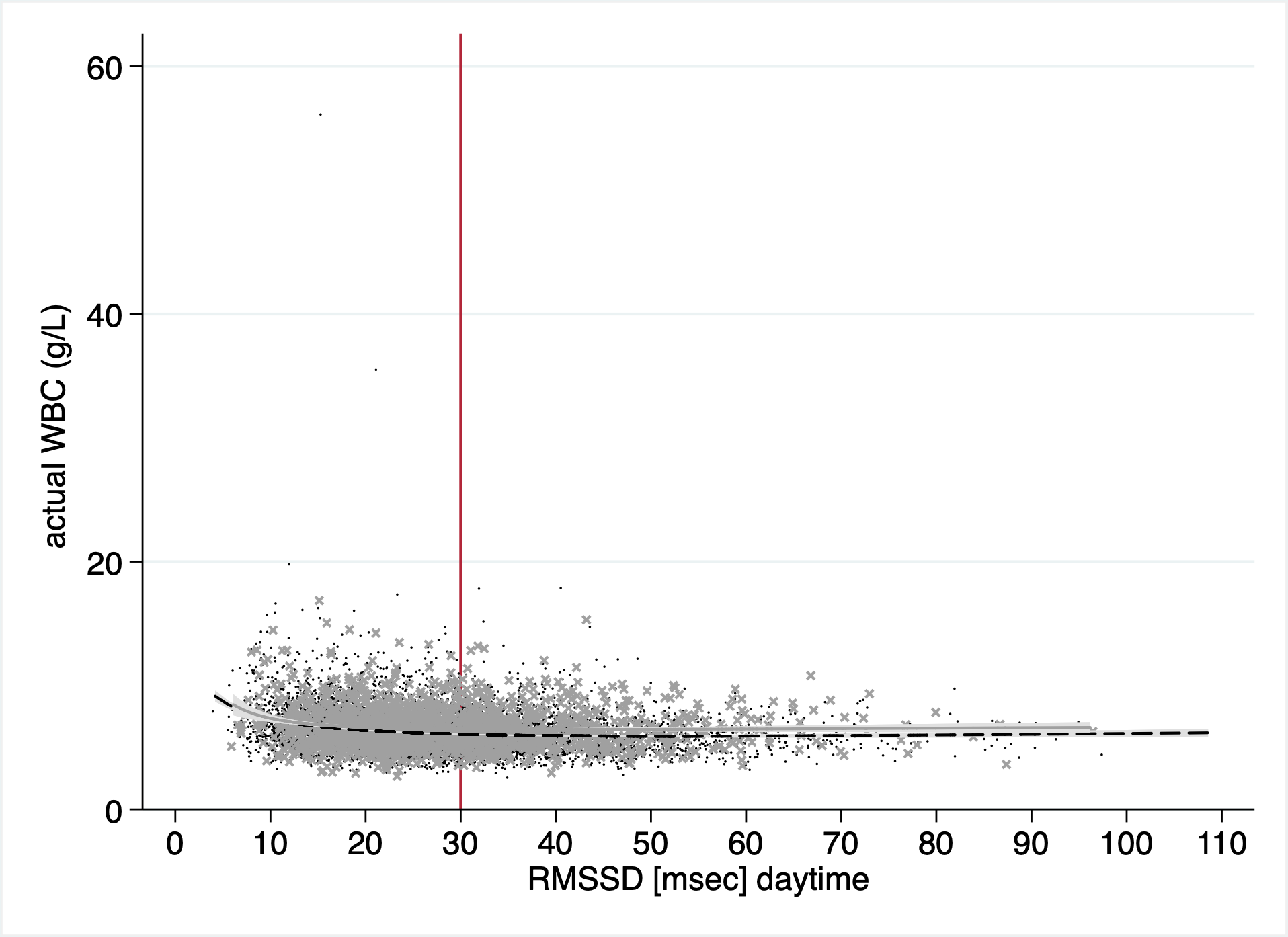

Supplement: Supplementary file 1 [file jcm-08-01940-s001.zip › supplements jcm_617360/leuk_actual_day.png]

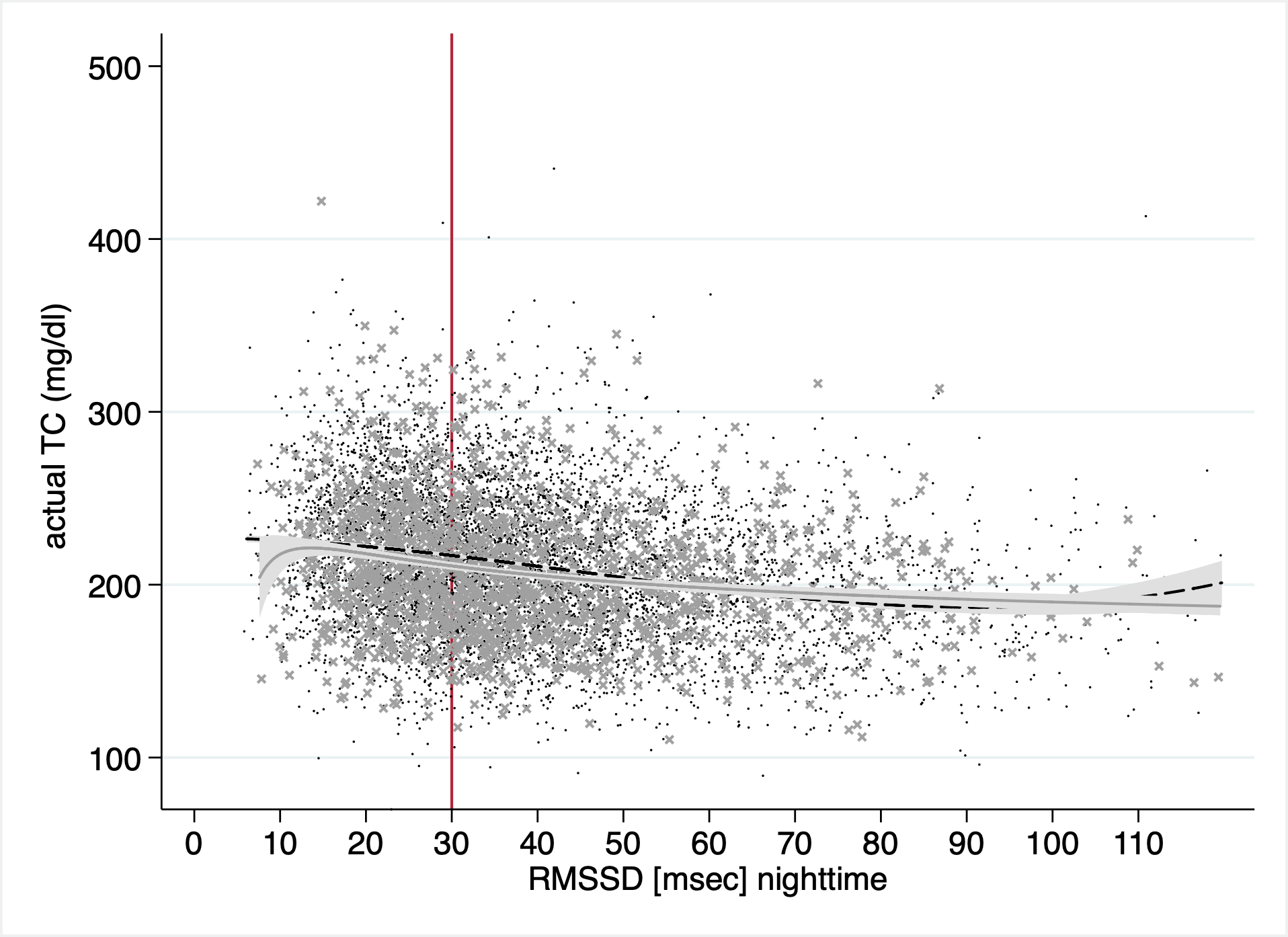

Supplement: Supplementary file 1 [file jcm-08-01940-s001.zip › supplements jcm_617360/chol_actual_night.png]

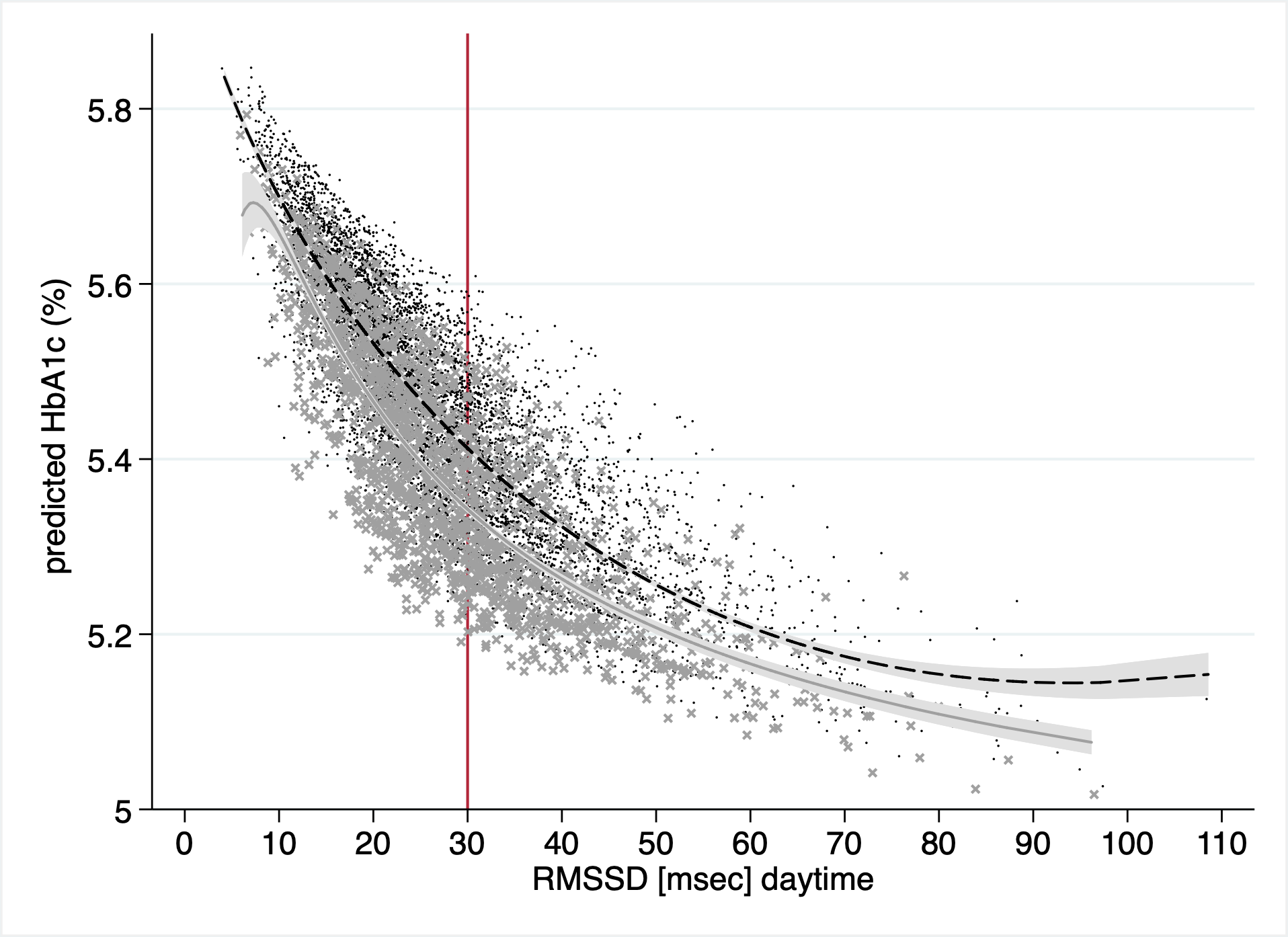

Supplement: Supplementary file 1 [file jcm-08-01940-s001.zip › supplements jcm_617360/hba1c_predicted_day.png]

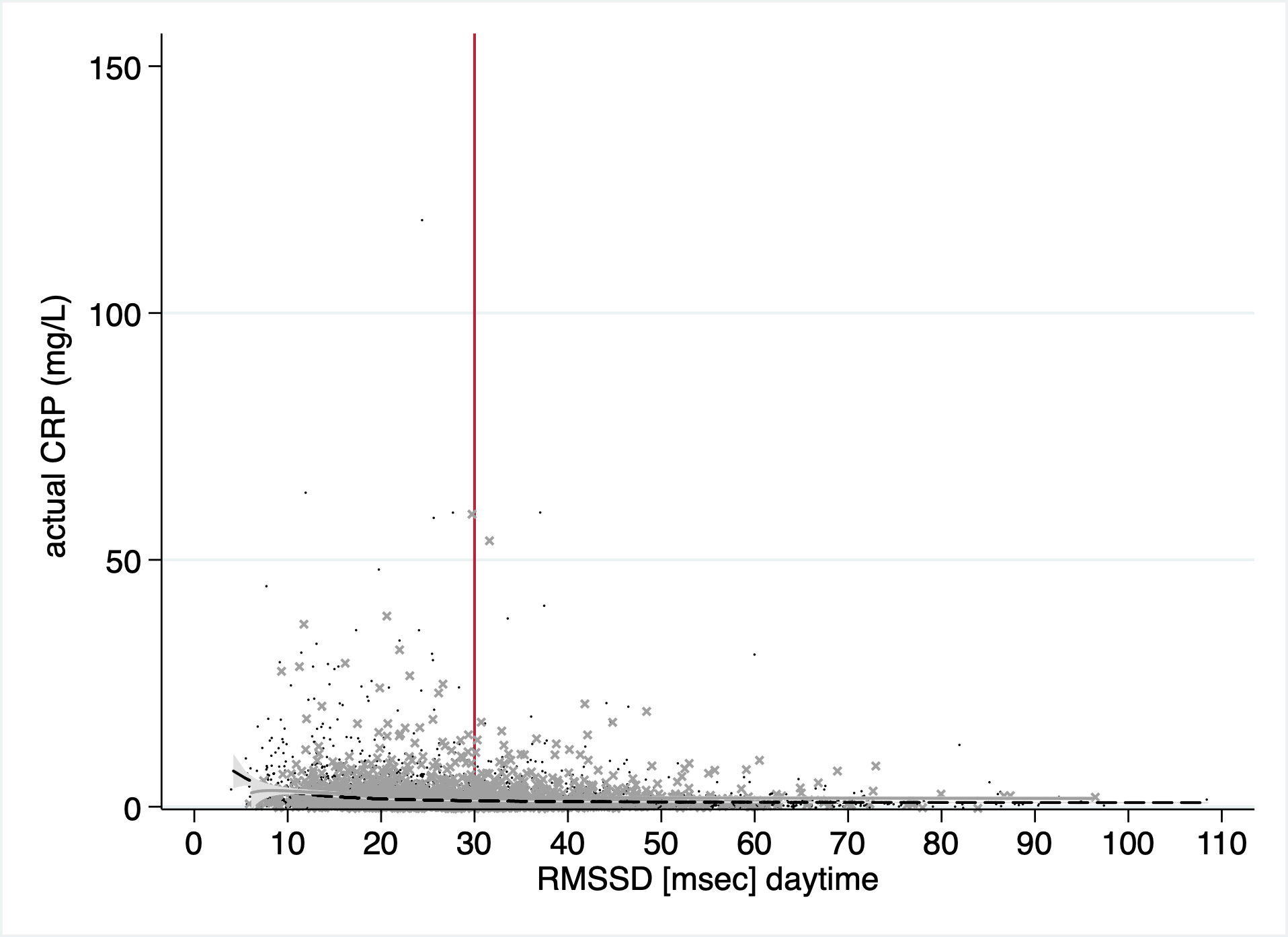

Supplement: Supplementary file 1 [file jcm-08-01940-s001.zip › supplements jcm_617360/crps_actual_day.png]
